# Supplementary material for: Impact of basic network motifs on the collective response to perturbations
Source: Nat Commun. 2022 Sep 8;13:5301. doi: 10.1038/s41467-022-32913-w (PMC9458749; doi:10.1038/s41467-022-32913-w)
Supplement: Supplementary file 1 — Supplementary Information [file 41467_2022_32913_MOESM1_ESM.pdf]

Impact of basic network motifs on collective response to perturbations

Xiaoge Bao[1,2,3], Qitong Hu[1,4], Peng Ji[1,2,3], Wei Lin[2,3,5,6,7], Jürgen Kurths[3,8,9], Jan Nagler[10,11] *

[1]Institute of Science and Technology for Brain-Inspired Intelligence, Fudan University, Shanghai, China

[2]Key Laboratory of Computational Neuroscience and Brain-Inspired Intelligence (Fudan University), Ministry of Education, China

[3]Research Institute of Intelligent Complex Systems and MOE Frontiers Center for Brain Science, Fudan University, Shanghai, China

[4]School of Mathematical Sciences, Shanghai Jiao Tong University, Shanghai, China

[5] School of Mathematical Sciences, SCMS, SCAM, and CCSB, Fudan University, Shanghai, China

[6]State Key Laboratory of Medical Neurobiology, Institutes of Brain Science, Fudan University, Shanghai, China

[7]Shanghai Artificial Intelligence Laboratory, Shanghai, China

[8]Potsdam Institute for Climate Impact Research, Germany

[9]Humboldt University, Berlin, Germany

[10]Deep Dynamics, Frankfurt School of Finance & Management, Frankfurt, Germany

[11]Centre for Human and Machine Intelligence, Frankfurt School of Finance & Management, Frankfurt, Germany

*The first two authors contributed equally. pengji@fudan.edu.cn, jan.nagler@gmail.com

Contents

1 Model Construction **3**

2 Local Propagation Time **3**

2.1 Quantification of $\delta_i(t)$ 3

2.2 Structural Split of $\mathcal{E}_{im}$ 5

2.3 Effect of Topological Characteristics 9

2.3.1 Effect of Intrinsic Dynamics 9

2.3.2 Effect of Independent Edges 11

2.3.3 Effect of Triangles 12

2.3.4 Brief Summary for Independent Edges 13

3 Global Propagation Time **16**

3.1 Quantification of $T(m \to i)$ 16

3.2 Structural Split of $\mathcal{E}_{i_k i_{k-1}}(T(m \to i_{k-1}))$ 17

3.3 Effect of Topological Characteristics 19

4 Dynamic Models **28**

4.1 Regulatory Dynamics 28

4.2 Human Dynamics 34

4.3 Other Dynamic Models 36

4.3.1 Epidemic Spreading Dynamics 36

4.3.2 Mutualistic Dynamics 38

4.3.3 Population Dynamics 40

4.3.4 Biochemical Dynamics 42

4.3.5 Inhibitory Dynamics 44

4.4 Brief Summary of Dynamic Models 46

5 Methods **50**

5.1 Applicability Analysis for Gauss Iteration Method 50

5.2 Quantification of Q_{im} by Iteration Method 53

5.3 Another Way to Quantify Global Propagation Time 55

1 Model Construction

We aim to track signal propagation in complex networks, with the following general dynamical equation

$$\dot{x_i}(t) = F(x_i(t)) + \sum_{j=1}^{N} A_{ij} H_1(x_i(t)) H_2(x_j(t)), \tag{1.1}$$

where $x_i(t), i = 1, ..., N$ represents node i's state, A_{ij} is the connectivity of the edge linking nodes i and j, and nonlinear functions ($F(x)$,$H_1(x)$,$H_2(x)$) uncover most commonly encountered dynamics as below: epidemic spreading, mutualistic, regulatory, inhibitory, population, human, biochemical and so on.

We consider the networked dynamics, governed by Eq. (1.1), is in a stationary stable state $\mathbf{x}^* = [x_1^*, ..., x_N^*]^T$, and through the perturbative approach, we induce a signal by perturbing node m with $x_m(t) = x_m^* + \Delta x_m(t)$, where $\Delta x_m(t)$ denotes perturbation. After perturbation, the signal propagates through the network, and nodes' states are subsequently affected, following

$$x_i(t) = x_i^* + \Delta x_i(t), \tag{1.2}$$

where $\Delta x_i(t)$ denotes i's displacements from the initial state x_i^*. Ultimately, the system reaches the stationary stable state with $x_i(\infty) = x_i^* + \Delta x_i(\infty)$. Tracking the cascade of response, we define the propagation time τ_{im} for the signal from nodes m to i through the response ratio $\delta_i(t)$

$$\delta_i(t) = \frac{\Delta x_i(t)}{\Delta x_i(\infty)}, \tag{1.3}$$

and the propagation time τ_{im} is defined when $\delta_i(t)$ reaches an $\eta-$ fraction, and it can be represented by the inverse function

$$\tau_{im} = \delta_i^{-1}(\eta). \tag{1.4}$$

2 Local Propagation Time

In this section, we formulate a general theoretical framework to quantify propagation time from a signal-induced node to its adjacent nodes. There are two cases we consider through the quantification, according to whether the signal-induced node and its adjacent node form a triangle or not.

2.1 Quantification of $\delta_i(t)$

After perturbations, the propagation time τ_{im} can be captured by the inverse function of relative i's response through Eq. (1.4). Consider a small permanent perturbation Δx_m towards node m, the state of its adjacent node i is slightly affected with $x_i(t) \approx x_i^*$. Linearizing the corresponding equation (1.1) yields

$$\begin{aligned} \Delta \dot{x}_i(t) &= \dot{x}_i(t) - \dot{x}_i^*, \\ &= -\frac{1}{J_i} \Delta x_i(t) + H_1(x_i^*) \sum_{\substack{j=1 \\ j \neq m}}^{N} A_{ij} H_2^{'}(x_j^*) \Delta x_j(t) + A_{im} H_1(x_i^*) H_2^{'}(x_m^*) \Delta x_m, \end{aligned} \tag{2.1}$$

where $H_2^{'}(x)$ represents the derivative dH_2/dx with x at initial states. J_i refers to i's intrinsic dynamics and

$$J_i = -1/\left(F^{'}(x_i^*) + H_1^{'}(x_i^*)\sum_{j=1}^{N} A_{ij}H_2(x_j^*)\right), \tag{2.2}$$

where $H_1^{'}(x)$ and $F^{'}(x)$ denote the derivatives dH_1/dx and dF/dx with x at initial states respectively. J_i can be further simplified by the initial state x_i^*. Thus the sum term can be rewritten as

$$\sum_{j=1}^{N} A_{ij}H_2(x_j^*) = -\frac{F(x_i^*)}{H_1(x_i^*)}. \tag{2.3}$$

Substituting Eq. (2.3) into Eq. (2.2), we derive

$$J_i = -1/\left(H_1(x_i^*)\left[\frac{F(x_i^*)}{H_1(x_i^*)}\right]^{'}\right). \tag{2.4}$$

Here, intrinsic dynamics J_i is determined by $F(x_i^*)$ and $H_1(x_i^*)$, and is independent of interactions from adjacent nodes.

After the system integration, we obtain the node i's displacements $\dot{x}_i(t)$ from the new stationary state $\dot{x}_i(\infty)$ as

$$\Delta\dot{x}_i(t) - \Delta\dot{x}_i(\infty) = -\frac{1}{J_i}\left(1 - \mathcal{E}_{im}(t)\right)\left[\Delta x_i(t) - \Delta x_i(\infty)\right], \tag{2.5}$$

where $\mathcal{E}_{im}(t)$ indicates effects from node i's neighbors, and its corresponding expression is

$$\begin{aligned}\mathcal{E}_{im}(t) &= J_iH_1(x_i^*)\sum_{\substack{j=1\\ j\neq m}}^{N} A_{ij}H_2^{'}(x_j^*)\frac{\Delta x_j(t) - \Delta x_j(\infty)}{\Delta x_i(t) - \Delta x_i(\infty)},\\ &= J_iH_1(x_i^*)\sum_{\substack{j=1\\ j\neq m}}^{N} A_{ij}H_2^{'}(x_j^*)\frac{\delta_j(t) - 1}{\delta_i(t) - 1}\frac{\Delta x_j(\infty)}{\Delta x_i(\infty)}.\end{aligned} \tag{2.6}$$

Take the difference of Eq. (2.5) to the left,

$$\frac{\Delta\dot{x}_i(\infty) - \Delta\dot{x}_i(t)}{\Delta x_i(\infty) - \Delta x_i(t)} = -\frac{1}{J_i}(1 - \mathcal{E}_{im}(t)). \tag{2.7}$$

Integrate both sides and obtain

$$\int_0^t \frac{\Delta\dot{x}_i(\infty) - \Delta\dot{x}_i(\tau)}{\Delta x_i(\infty) - \Delta x_i(\tau)}d\tau = -\frac{1}{J_i}\int_0^t (1 - \mathcal{E}_{im}(\tau))d\tau. \tag{2.8}$$

Then we derive

$$\ln(\Delta x_i(\infty) - \Delta x_i(t)) - \ln(\Delta x_i(\infty)) = -\frac{1}{J_i}\int_0^t (1 - \mathcal{E}_{im}(\tau))d\tau, \tag{2.9}$$

also, i.e.,

$$\ln(1 - \delta_i(t)) = -\frac{1}{J_i}\int_0^t (1 - \mathcal{E}_{im}(\tau))d\tau. \tag{2.10}$$

Therefore, we derive the corresponding representation of $\delta_i(t)$ as

$$\delta_i(t) = 1 - e^{-\frac{1}{J_i}(t - \int_0^t \mathcal{E}_{im}(\tau)d\tau)}. \tag{2.11}$$

The propagation time τ_{im} can be determined when $\delta_i(t)$ reaches the fraction η.

2.2 Structural Split of $\mathcal{E}_{im}$

In the former subsection, we have derived the corresponding equation of the propagation time and found that the propagation time could be characterized by the interplay between target node's adjacent dynamics and its intrinsic dynamics. In what follows, we further investigate the expression of $\mathcal{E}_{im}$ in terms of motifs.

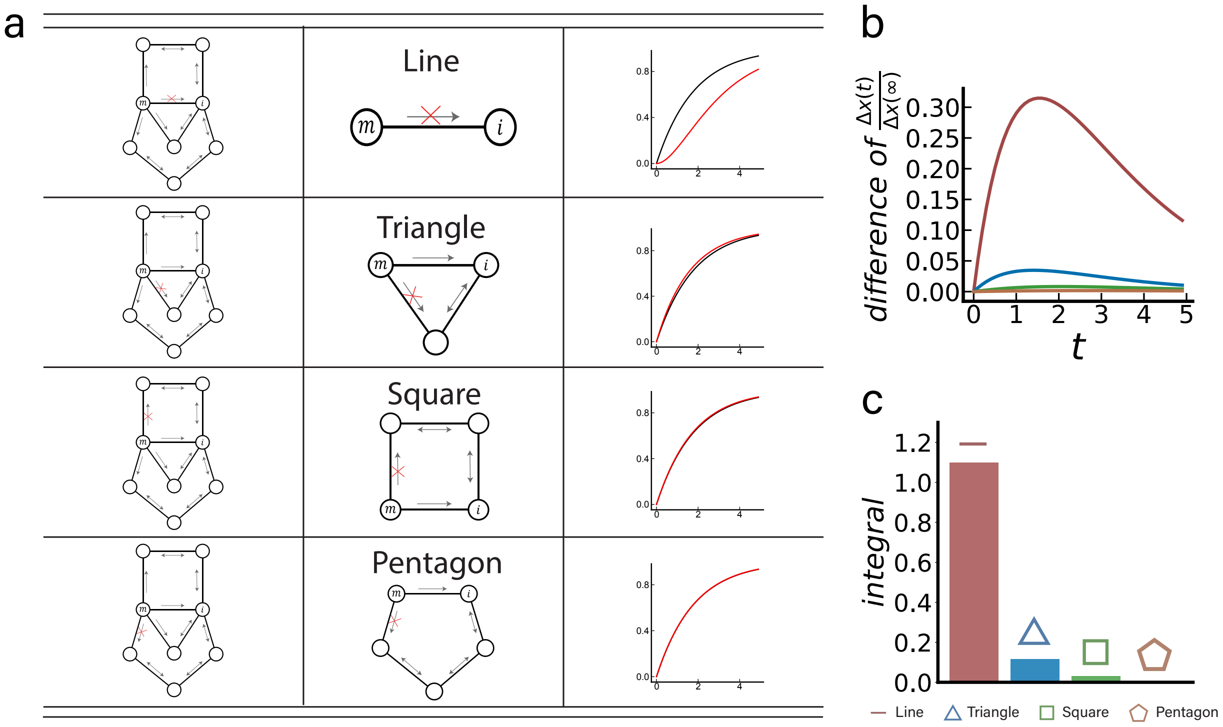

Table S1: (a) Schematic table of a signal penetrating through different motifs from a source node towards target. Source m receives perturbation $\Delta x_m = 0.3$ and source's adjacent node i is taken as a target. The arrows represent the direction of the signal flow. To quantify the importance of each motif, we isolate the signal flow spreading though each motif connected with m respectively, i.e., breaking line, triangle, square, and pentagon respectively as shown in the second column. The evolution of i's relative response $\frac{\Delta x_i(t)}{\Delta x_i(\infty)}$ under regulatory dynamics ($a = 0.8, b = 0.5, \alpha = 1.0, B = 1.0$) in the third column indicates that the effect of signal flow crossing the line is the most significant, and then triangles, and so on. (b) Absolute difference of the evolution of i's relative response $\frac{\Delta x_i(t)}{\Delta x_i(\infty)}$ in (a) before and after the isolation of signal flow for edge (red), triangle (blue), square (green), and pentagon (brown). (c) Integral of absolute difference of the evolution of i's relative response $\frac{\Delta x_i(t)}{\Delta x_i(\infty)}$ in (b) for edge (red), triangle (blue), square (green), and pentagon (brown). Both in (b) and (c), edges and triangles occupy the vital roles in signal spreading.

As illustrated in Table S1, edge and triangle play important roles in signal spreading according to comparison before and after the isolation of signal flow, while polygon with more than 3 edges has marginal effects. Also in Table S2, the difference between double triangles and single tetrahedron is limited,

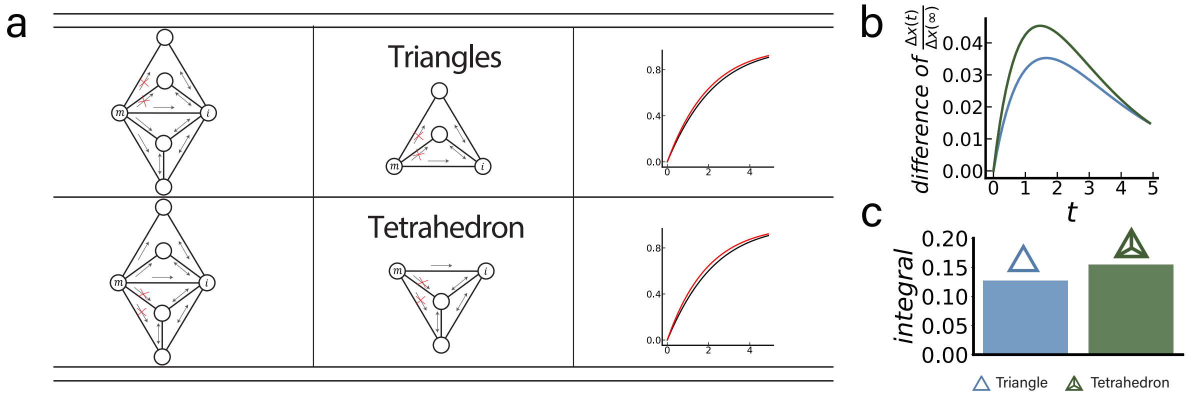

Table S2: (a) Schematic table of a signal penetrating through double triangles and single tetrahedron from a source node towards target. Source m receives perturbation $\Delta x_m = 0.3$ and source's adjacent node i is taken as a target. The arrows represent the direction of the signal flow. To quantify the importance of triangles and tetrahedrons, we isolate the signal flow spreading though triangles and tetrahedrons connected with m respectively, shown in the second column. The evolution of i's relative response $\frac{\Delta x_i(t)}{\Delta x_i(\infty)}$ under regulatory dynamics ($a = 0.8, b = 0.5, \alpha = 1.0, B = 1.0$) in the third column indicates that the effect of tetrahedrons is slightly more obvious than triangles. (b) Absolute difference of the evolution of i's relative response $\frac{\Delta x_i(t)}{\Delta x_i(\infty)}$ in (a) before and after the isolation of signal flow for triangle (blue) and tetrahedron (dark green). (c) Integral of absolute difference of the evolution of i's relative response $\frac{\Delta x_i(t)}{\Delta x_i(\infty)}$ in (b) for triangle (blue) and tetrahedron (dark green). Both in (b) and (c), tetrahedrons influence signal flow more compared to triangles, but their differences are not so large.

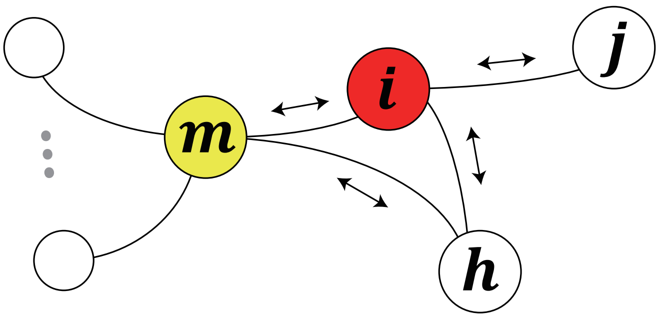

Figure S1: Schematic diagram of signal propagation flowing from a source towards its adjacent target through different local topological structures. Yellow source node m is perturbed to induce signal. We focus on the response of the red target node i. Node j is adjacent to target i but not to source m, and is taken a contribution to an independent edge. While source m, target i and i's adjacency h form a triangle. The directional arrow represents direction of the signal flow.

inspiring that the analysis of motifs (like tetrahedrons here) can be spitted into multiple simpler motifs (like triangles here), which simplify the analysis procedure. Therefore, we evaluate the contribution of the propagation time τ_{im} from i's adjacent nodes' local structures. Concretely, we portray the diagrammatic as shown in Fig. S1: take K_i as the set of i's neighbors, and then divide $K_i \backslash \{m\}$ into two subsets of Δ_{im} and L_{im}. Δ_{im} is the set comprising common neighbors of nodes i and m, and a node h in Δ_{im} together with nodes i and m form *triangle.* The set L_{im} consists of nodes which are linked with node i but not with node m, and a node j in L_{im} with i forming a line defined as *independent edge.* To evaluate the contributions of these two subsets to the signal propagation, we separate the integral $\int_0^{\tau_{im}} \mathcal{E}_{im}(\tau)d\tau$ into their corresponding parts as

$$\int_0^{\tau_{im}} \mathcal{E}_{im}(\tau)d\tau = \int_0^{\tau_{im}} \mathcal{E}_{L_{im}}(\tau)d\tau + \int_0^{\tau_{im}} \mathcal{E}_{\Delta_{im}}(\tau)d\tau. \tag{2.12}$$

For the first contribution of the integral $\int_0^{\tau_{im}} \mathcal{E}_{im}(\tau)d\tau$ from the nodes in L_{im}, the corresponding definite integral is written as

$$\int_0^{\tau_{im}} \mathcal{E}_{L_{im}}(\tau)d\tau = J_i H_1(x_i^*) \sum_{j\in L_{im}} A_{ij} H_2^{'}(x_j^*) \frac{\Delta x_j(\infty)}{\Delta x_i(\infty)} \int_0^{\tau_{im}} \frac{\delta_j(\tau)-1}{\delta_i(\tau)-1} d\tau. \tag{2.13}$$

As node j constitutes an independent edge, its state varies slightly within a short time period, and $\delta_j(t)$ could be set as zero during $[0, \tau_{im}]$. The expression becomes

$$\int_0^{\tau_{im}} \mathcal{E}_{L_{im}}(\tau)d\tau = J_i H_1(x_i^*) \sum_{j\in L_{im}} A_{ij} H_2^{'}(x_j^*) \frac{\Delta x_j(\infty)}{\Delta x_i(\infty)} \int_0^{\tau_{im}} e^{\frac{1}{J_i}(\tau - \int_0^{\tau} \mathcal{E}_{im}(u)du)} d\tau. \tag{2.14}$$

By using the mean value theorem of integrals, we obtain

$$\begin{aligned} \int_0^{\tau_{im}} \mathcal{E}_{L_{im}}(\tau)d\tau &= J_i H_1\left(x_i^*\right) \sum_{j\in L_{im}} A_{ij} H_2'\left(x_j^*\right) \frac{\Delta x_j(\infty)}{\Delta x_i(\infty)} \int_0^{\tau_{im}} e^{\frac{1}{J_i}(1-\mathcal{E}_{im}(\xi_{L_{im}}))\tau} d\tau, \\ &= J_i H_1\left(x_i^*\right) \sum_{j\in L_{im}} A_{ij} H_2'\left(x_j^*\right) \frac{\Delta x_j(\infty)}{\Delta x_i(\infty)} \frac{e^{\frac{1}{J_i}(1-\mathcal{E}_{im}(\xi_{L_{im}}))\tau_{im}} - 1}{\frac{1}{J_i}\left(1-\mathcal{E}_{im}\left(\xi_{L_{im}}\right)\right)}, \end{aligned} \tag{2.15}$$

where $\xi_{L_{im}} \in [0, \tau_{im}]$. When $\delta_i(t)$ reaches η, Eq. (2.11) is transformed into

$$\begin{aligned} 1-\eta &= e^{-\frac{1}{J_i}(\tau_{im} - \int_0^{\tau_{im}} \mathcal{E}_{im}(u)du)}, \\ &= e^{-\frac{1}{J_i}(1-\mathcal{E}_{im}(\zeta_{L_{im}}))\tau_{im}}, \end{aligned} \tag{2.16}$$

and mean effects $\mathcal{E}_{im}(\zeta_{L_{im}}) \approx \mathcal{E}_{im}(\xi_{L_{im}})$, thus the approximation holds

$$\ln(1-\eta) = -\frac{1}{J_i}(1-\mathcal{E}_{im}(\xi_{L_{im}}))\tau_{im}. \tag{2.17}$$

Substituting Eq. (2.17) into Eq. (2.15), we obtain

$$\int_0^{\tau_{im}} \mathcal{E}_{L_{im}}(\tau)d\tau = \frac{1-e^{-\ln(1-\eta)}}{\ln(1-\eta)} \tau_{im} \mathcal{E}_{L_{im}}(0), \tag{2.18}$$

where $\mathcal{E}_{L_{im}}(0) = J_i H_1\left(x_i^*\right) \sum_{j\in L_{im}} A_{ij} H_2'\left(x_j^*\right) \frac{\Delta x_j(\infty)}{\Delta x_i(\infty)}$. Node j in group L_{im} is mainly affected by the node i, and the governing equation of $\Delta x_j(t)$ follows

$$\Delta \dot{x}_j(t) = -\frac{1}{J_j}\Delta x_j(t) + H_1(x_j^*) \sum_{\substack{k=1 \\ k\neq i}}^{N} A_{jk} H_2^{'}(x_k^*) \Delta x_k(t) + A_{ji} H_1(x_j^*) H_2^{'}(x_i^*) \Delta x_i(t). \tag{2.19}$$

When $t \to \infty$, the division of $\Delta x_j(\infty)$ and $\Delta x_i(\infty)$ becomes

$$\frac{\Delta x_j(\infty)}{\Delta x_i(\infty)} = \frac{A_{ji} J_j H_1(x_j^*) H_2^{'}(x_i^*)}{1 - \mathcal{E}_{ji}(0)}, \tag{2.20}$$

where $\mathcal{E}_{ji}(0)$ is defined as $J_j H_1(x_j^*) \sum_{\substack{k=1 \\ k\neq i}}^{N} A_{jk} H_2^{'}(x_k^*) \Delta x_k(\infty) = \mathcal{E}_{ji}(0) \Delta x_j(\infty)$.

Therefore, after substituting Eq. (2.20) into Eq. (2.18) and neglecting the marginal effect $\mathcal{E}_{ij}(0)$ (In Section Methods, we consider the influence of $\mathcal{E}_{ji}(0)$ and here take the initial iterative term for simplification), we derive the first contribution of the integral $\int_0^{\tau_{im}} \mathcal{E}_{im}(\tau) d\tau$ from L_{im} as

$$\int_0^{\tau_{im}} \mathcal{E}_{L_{im}}(\tau) d\tau = -\frac{1}{\ln(1-\eta)} \frac{\eta}{1-\eta} Q_i \sum_{j \in L_{im}} A_{ij}^2 Q_j \tau_{im}, \tag{2.21}$$

where $Q_j = J_j H_1\left(x_j^*\right) H_2'\left(x_j^*\right)$.

For the second contribution of the integral $\int_0^{\tau_{im}} \mathcal{E}_{im}(\tau) d\tau$ from of nodes in Δ_{im}. Node j in Δ_{im} is mainly affected by the source node m and its state's variation could be expressed similarly with i's, i.e., $\delta_j(t) = 1 - e^{-\frac{1}{J_j}(t - \int_0^t \mathcal{E}_{jm}(\tau) d\tau)}$. Substituting expressions of $\delta_i(t)$ and $\delta_j(t)$ into the representation of $\mathcal{E}_{\Delta_{im}}(\tau)$ yields

$$\int_0^{\tau_{im}} \mathcal{E}_{\Delta_{im}}(\tau) d\tau = J_i H_1(x_i^*) \sum_{j \in \Delta_{im}} A_{ij} H_2^{'}(x_j^*) \frac{\Delta x_j(\infty)}{\Delta x_i(\infty)} \int_0^{\tau_{im}} \frac{e^{-\frac{1}{J_j}(\tau - \int_0^{\tau} \mathcal{E}_{jm}(u) du)}}{e^{-\frac{1}{J_i}(\tau - \int_0^{\tau} \mathcal{E}_{im}(u) du)}} d\tau. \tag{2.22}$$

Consider node i with relatively large degree compared with degrees of i's neighbors, effect of m towards j denoted as $\mathcal{E}_{jm}$ can be omitted, and the corresponding equation after employing mean value theorem can be simplified as

$$\int_0^{\tau_{im}} \mathcal{E}_{\Delta_{im}}(\tau) d\tau = J_i H_1(x_i^*) \sum_{j \in \Delta_{im}} A_{ij} H_2^{'}(x_j^*) \frac{\Delta x_j(\infty)}{\Delta x_i(\infty)} \frac{e^{\left(-\frac{1}{J_j} + \frac{1}{J_i}(1 - \mathcal{E}_{im}(\xi_{\Delta_{im}}))\right)\tau_{im}} - 1}{-\frac{1}{J_j} + \frac{1}{J_i}(1 - \mathcal{E}_{im}(\xi_{\Delta_{im}}))}, \tag{2.23}$$

where $\xi_{\Delta_{im}} \in [0, \tau_{im}]$. When $\delta_i(t)$ reaches η, Eq. (2.11) will be transformed into an approximation

$$\ln(1-\eta) \approx -\frac{1}{J_i}(1 - \mathcal{E}_{im}(\xi_{\Delta_{im}}))\tau_{im}. \tag{2.24}$$

As node j in Δ_{im} is mainly affected by the source node m, the division of $\Delta x_j(\infty)$ and $\Delta x_i(\infty)$ can be formulized by Δx_m,

$$\frac{\Delta x_j(\infty)}{\Delta x_i(\infty)} = \frac{\Delta x_j(\infty)}{\Delta x_m} \frac{\Delta x_m}{\Delta x_i(\infty)}. \tag{2.25}$$

Through final states of the system, the left side of Eqs. (2.1) and (2.19) vanishes. Then we represent scales as

$$\begin{cases} \frac{\Delta x_i(\infty)}{\Delta x_m} = \frac{A_{im} J_i H_1(x_i^*) H_2^{'}(x_m^*)}{1 - \mathcal{E}_{im}(0)}, \\ \frac{\Delta x_j(\infty)}{\Delta x_m} = \frac{A_{jm} J_j H_1(x_j^*) H_2^{'}(x_m^*)}{1 - \mathcal{E}_{jm}(0)}, \end{cases} \tag{2.26}$$

where $\mathcal{E}_{im}(0) = J_i H_1\left(x_i^*\right) \sum_{\substack{j=1 \\ j\neq m}} A_{ij} H_2'\left(x_j^*\right) \frac{\Delta x_j(\infty)}{\Delta x_i(\infty)}$. By Eq. (2.26), we simplify Eq. (2.25) as

$$\frac{\Delta x_j(\infty)}{\Delta x_i(\infty)} = \frac{A_{jm} J_j}{A_{im} J_i} \frac{H_1(x_j^*)}{H_1(x_i^*)} \frac{1 - \mathcal{E}_{im}(0)}{1 - \mathcal{E}_{jm}(0)}. \tag{2.27}$$

Substituting Eqs. (2.24) and (2.27) into Eq. (2.23) and neglecting the marginal effect $\mathcal{E}_{jm}(0)$, we derive the contribution of the integral $\int_0^{\tau_{im}} \mathcal{E}_{im}(\tau)d\tau$ from Δ_{im} as

$$\begin{aligned}\int_0^{\tau_{im}} \mathcal{E}_{\Delta_{im}}(\tau)d\tau &= (1-\mathcal{E}_{im}(0))\,\tau_{im}\sum_{j\in\Delta_{im}}\frac{A_{ij}A_{jm}}{A_{im}}Q_j f(\tau_{im},J_j),\\ &\approx (1-\mathcal{E}_{im}(0))\,\tau_{im}\overline{f(\tau_{im})}\sum_{j\in\Delta_{im}}\frac{A_{ij}A_{jm}}{A_{im}}Q_j\end{aligned} \tag{2.28}$$

where

$$f(\tau_{im},J_j)=\begin{cases}-\frac{\eta}{\ln(1-\eta)(1-\eta)},\ \frac{\tau_{im}}{J_j}\to 0,\\ 1,\ \frac{\tau_{im}}{J_j}\to -\ln(1-\eta),\\ 0,\ \frac{\tau_{im}}{J_j}\to +\infty,\end{cases} \tag{2.29}$$

and considering all possible cases for triangles, we denote $\overline{f(\tau_{im})}$ as the average value of $f(\tau_{im}, J_j)$. Therefore, we combine the contributions of these two subsets Eqs. (2.21) and (2.28) to the signal propagation, we derive

$$\int_0^{\tau_{im}} \mathcal{E}_{im}(\tau)d\tau = \left(-\frac{1}{\ln(1-\eta)}\frac{\eta}{1-\eta}\mathcal{I}_{L_{im}}+\frac{1-\mathcal{I}_{L_{im}}}{1+\mathcal{I}_{\Delta_{im}}}\mathcal{I}_{\Delta_{im}}\overline{f(\tau_{im})}\right)\tau_{im}, \tag{2.30}$$

where

$$\begin{cases}J_i=-1/\left(H_1(x_i^*)\left[\frac{F(x_i^*)}{H_1(x_i^*)}\right]'\right),\\ \mathcal{I}_{\Delta_{im}}=\sum\limits_{j\in\Delta_{im}}\frac{A_{ij}A_{jm}}{A_{im}}Q_j,\\ \mathcal{I}_{L_{im}}=Q_i\sum\limits_{j\in L_{im}}A_{ij}^2Q_j.\end{cases} \tag{2.31}$$

Substituting the representation Eq. (2.30) into Eq. (2.11), we summarize that

$$\ln(1-\eta)J_i+\tau_{im}=\left(-\frac{1}{\ln(1-\eta)}\frac{\eta}{1-\eta}\mathcal{I}_{L_{im}}+\frac{1-\mathcal{I}_{L_{im}}}{1+\mathcal{I}_{\Delta_{im}}}\mathcal{I}_{\Delta_{im}}\overline{f(\tau_{im})}\right)\tau_{im}. \tag{2.32}$$

If we assumed that the states of adjacent nodes jump instantly to their new stationary state, $\Delta x_j(\tau_{im}) \approx \Delta x_j(\infty)$, the contribution $\mathcal{E}_{im}(t)$ would vanish, $\mathcal{E}_{im}(t) \to 0$. In this case, via eliminating the effect of the right side of Eq. (2.32), we reproduce the corresponding equation in [1], which hypothesized three distinct spatiotemporal scaling regimes.

2.3 Effect of Topological Characteristics

The quantification of $\mathcal{E}_{im}$ provides dynamical insights through embodying topological elements. The expression of Eq. (2.32) unravels that the propagation time is affected by three factors: the nodal intrinsic dynamics J_i, the effect of independent edges $\mathcal{I}_{L_{im}}$ and the effect of triangles $\mathcal{I}_{\Delta_{im}}$.

2.3.1 Effect of Intrinsic Dynamics

Firstly, we consider the effects of nodal intrinsic dynamics J_i, and derive the expression via Eq. (1.1) to describe x_i in the initial stationary state,

$$F(x_i^*)=-H_1(x_i^*)\sum_{j=1}^{N}A_{ij}H_2(x_j^*). \tag{2.33}$$

To simplify i's coupling terms and induce function of nodal degree, mean−field theory was exploited [1], and the sum function is transformed into a mean value, i.e., $\mathcal{H}_i = \frac{1}{d_i}\sum_{j=1}^{N} A_{ij} H_2\big(x_j^*\big)$. We consider that probabilities of connectivity between two arbitrary nodes are the same, and then $\{\mathcal{H}_i\}$ are the same for all nodes. Thus we set the mean function $\overline{\mathcal{H}}$, which is independent of nodes, as

$$\overline{\mathcal{H}} = \frac{1}{N}\sum_{i=1}^{N}\frac{1}{d_i}\sum_{j=1}^{N} A_{ij} H_2(x_j^*). \tag{2.34}$$

The relationship between the degree and the node's state under dynamic mechanisms follows

$$\frac{F(x_i^*)}{H_1(x_i^*)} = -d_i\overline{\mathcal{H}}. \tag{2.35}$$

We denote $R(x) = -\frac{F(x)}{H_1(x)}$, and the initial state x_i^* can be calculated by the inverse function

$$x_i^* = R^{-1}\big(d_i\overline{\mathcal{H}}\big). \tag{2.36}$$

Therefore, i's final state is induced by the combination of self−degree, degree distribution of the network and intrinsic dynamics. Given a large−scale network with a suitable dynamic model, the local property could determine the node's final state. For further demonstrating degree's influence towards J_i intuitively, we focus on two specific extreme cases which are $d_i \to 1$ and $d_i \to \infty$ respectively.

For the small degree, we have the Taylor expansion of J_i at $d_0 = 1$ as

$$J_i = -1/\left(H_1(x_i^*)\left[\frac{F(x_i^*)}{H_1(x_i^*)}\right]'\right)\Big|_{x_i^*=R^{-1}\left(d_i\overline{\mathcal{H}}\right)} = \sum_{k=0}^{\infty} U_k\,(d_i-1)^k\,. \tag{2.37}$$

The corresponding derivative is determined by the leading term U_1,

$$\lim_{d_i\to 1}\,[J_i]' \sim U_1, \tag{2.38}$$

and the correlation relation between J_i and d_i is confirmed through the sign of U_1.

For the large degree, we employ the generalization of Taylor expansion to deal with the relation between J_i and d_i referring to infinity degree d_i, Hahn expansion [2]

$$J_i = -1/\left(H_1(x_i^*)\left[\frac{F(x_i^*)}{H_1(x_i^*)}\right]'\right)\Big|_{x_i^*=R^{-1}\left(d_i\overline{\mathcal{H}}\right)} = \sum_{n=0}^{\infty} U_n d_i^{\Pi_J^{(n)}}, \tag{2.39}$$

where real powers satisfy $\Pi_J^{(n+1)} > \Pi_J^{(n)}$ and d_0 is chosen as 0. In this situation, the expansion of J_i is mastered by the maximal power set as $\Pi_J^{(\infty)}$.

$$\lim_{d_i\to\infty} J_i \sim U_\infty d_i^{\Pi_J^{(\infty)}}, \tag{2.40}$$

and J_i is determined by U_∞'s signature. In most dynamic models, J_i is positive and U_∞ therein is positive. The derivative of J_i becomes

$$\lim_{d_i\to\infty}\,[J_i]' \sim \Pi_J(\infty) U_\infty d_i^{\Pi_J(\infty)-1}. \tag{2.41}$$

Hence, $\Pi_J(\infty)$'s signature decides the trends for J_i following the variation of d_i, and we define the scaling coefficient θ_J as $\theta_J = \Pi_J(\infty)$.

2.3.2 Effect of Independent Edges

Secondly, we consider the effect of independent edges $\mathcal{I}_{L_{im}}$. Number of i's independent edges is set as s_{im}, triangles' number is t_{im}, and $s_{im} + t_{im} + 1 = d_i$. The effect of independent edges shown in the former context is

$$\mathcal{I}_{L_{im}} = Q_i \sum_{j \in L_{im}} A_{ij}^2 Q_j. \tag{2.42}$$

By employing the mean−filed theory, we obtain the mean value $\overline{\mathcal{Q}}_L$ of the coupling term $\sum_{j \in L_{im}} A_{ij}^2 Q_j$ as

$$\overline{\mathcal{Q}}_L = \frac{1}{d_m} \sum_{i \in K_m} \frac{1}{s_{im}} \sum_{j \in L_{im}} A_{ij}^2 Q_j. \tag{2.43}$$

Different from the mean coupling term Eq. (2.34) exploited in [1], $\overline{\mathcal{Q}}_L$ for independent edges here indicates the average interactions across m's neighbors in a local regime rather than the whole network.

Then the effect of independent edges becomes

$$\mathcal{I}_{L_{im}} = s_{im} Q_i \overline{\mathcal{Q}}_L. \tag{2.44}$$

Q_i is the function of d_i so $\mathcal{I}_{L_{im}}$ is d_i's function as well. Similar with analysis of J_i, $\mathcal{I}_{L_{im}}$ can also be expanded in two extreme cases according to i's degree. Take the mean feature $\overline{\mathcal{Q}}_L$ as constant, we first analyze the effect of independent edges when degree is small enough based on Taylor expansion at $d_0 = 1$. At this point, $t_{im} = 0$. $Q_i = \sum_{k=0}^{\infty} V_k (d_i - 1)^k$ and its signature is determined by V_0. Substituting Q_i into Eq. (2.44), we derive

$$\mathcal{I}_{L_{im}} = \sum_{k=0}^{\infty} V_k (d_i - 1)^{k+1} \overline{\mathcal{Q}}_L. \tag{2.45}$$

The derivative is determined by

$$\lim_{d_i \to 1} [\mathcal{I}_{L_{im}}]' \sim V_0 \overline{\mathcal{Q}}_L. \tag{2.46}$$

As the initial iterative term Q_i and the mean value $\overline{\mathcal{Q}}_L$ remains constant, $[\mathcal{I}_{L_{im}}]'$ always maintain positive when degree is small enough.

When d_i approaches infinity, we obtain $\lim_{d_i \to \infty} Q_i \sim V_\infty d_i^{\Pi_Q(\infty)}$ and the signature of Q_i is determined by V_∞. In this case, s_{im} is approximate to d_{im} as the number of triangles is limited while the degree d_i is big enough. Hence, the effect of independent edges becomes

$$\lim_{d_i \to \infty} \mathcal{I}_{L_{im}} \sim V_\infty d_i^{\Pi_Q(\infty)+1} \overline{\mathcal{Q}}_L, \tag{2.47}$$

with the corresponding derivative

$$\lim_{d_i \to \infty} [\mathcal{I}_{L_{im}}]' \sim \theta_Q V_\infty d_i^{\theta_Q - 1} \overline{\mathcal{Q}}_L, \tag{2.48}$$

where $\theta_Q = \Pi_Q(\infty) + 1$. As signatures of Q_i and $\overline{\mathcal{Q}}_L$ are the same, $V_\infty \overline{\mathcal{Q}}_L$ have no influences on the scaling coefficient and signature of the derivative is determined by θ_Q.

To further manifest the effect of independent edges, we consider $s_{im} + 1 = d_i$ and obtain

$$\ln(1-\eta) J_i + \tau_{im} = -\frac{1}{\ln(1-\eta)} \frac{\eta}{1-\eta} \tau_{im} \mathcal{I}_{L_{im}}. \tag{2.49}$$

Hence, τ_{im} could be written as

$$\tau_{im} = \frac{-J_i \ln(1-\eta)}{1 + \frac{1}{\ln(1-\eta)} \frac{\eta}{1-\eta} \mathcal{I}_{L_{im}}}. \tag{2.50}$$

In the situation we obtain the explicit quantification of the propagation time. When $d_i \to \infty$, the propagation time can be further rewritten as

$$\tau_{im} = \frac{-U_\infty d_i^{\theta_J} \ln(1-\eta)}{1 + \frac{1}{\ln(1-\eta)} \frac{\eta}{1-\eta} Q d_i^{\theta_Q}}, \tag{2.51}$$

where $Q = V_\infty \overline{\mathcal{Q}}_L$.

The propagation time becomes independent of m, and if $\theta_Q < 0$,

$$\tau_i \sim d_i^{\theta_J}. \tag{2.52}$$

The contribution of adjacent dynamics $\mathcal{I}_{L_{im}}$ therein would vanish, and scaling exponent τ_i is determined by the system dynamics but independent of the network. In this case, we are able to reproduce the previous work [1], where three highly distinctive dynamic regimes have been predicated. This scaling relationship (2.52) links the response time τ_i to i's degree d_i. This highlights the contribution of both the topological elements and system dynamics on the response time. To reveal universal features, statistical physics in general neglects prefactors and focuses only on scaling exponents for the scaling analyses [3]. For the scaling prediction, results could remain consistent given two different derivation tracks although with different intermediate realizations [4]. While, for the correct predictions of the response time, a suitable prefactor in the scaling should be taken care of [5]. Anyway, the tools of scaling analysis can be particularly powerful but with proper usage and with explicit mathematical definition [6].

If $\theta_Q > 0$, it will be mainly determined by $d_i^{\theta_J - \theta_Q}$ as

$$\tau_i \sim d_i^{\theta_J - \theta_Q}. \tag{2.53}$$

2.3.3 Effect of Triangles

Thirdly, we consider the effect of triangles $\mathcal{I}_{\Delta_{im}}$. By employing the mean−field theory, we calculate the mean coupling term $\sum\limits_{j \in \Delta_{im}} \frac{A_{ij} A_{jm}}{A_{im}} Q_j$ as

$$\overline{\mathcal{Q}}_\Delta = \frac{1}{d_m} \sum_{i \in K_m} \frac{1}{t_{im}} \sum_{j \in \Delta_{im}} \frac{A_{ij} A_{jm}}{A_{im}} Q_j. \tag{2.54}$$

This mean coupling term is different from $\overline{\mathcal{H}}$ in Eq. (2.34) as it denotes the mean interactions of triangles across m's neighbors rather than nodes of the whole network. The effect of triangles $\mathcal{I}_{\Delta_{im}}$ follows

$$\mathcal{I}_{\Delta_{im}} = t_{im} \overline{\mathcal{Q}}_\Delta. \tag{2.55}$$

Whether the impact of triangles is positive or negative follows the sign of $\overline{\mathcal{Q}}_\Delta$ but the mean value $\overline{\mathcal{Q}}_\Delta$ depends on the specific network with specific dynamic models. Generally, the relation between propagation time and number of triangles generates

$$\mathcal{I}_{\Delta_{im}} \sim sign(\overline{\mathcal{Q}}_\Delta) t_{im}. \tag{2.56}$$

To further manifest the effect of triangles, consider $t_{im}+1=d_i$ and the effect of independent edges vanishes, i.e., $\mathcal{I}_{L_{im}}=0$. We obtain the propagation time as

$$\ln(1-\eta)J_i+\tau_{im}=\overline{f(\tau_{im})}\frac{\mathcal{I}_{\Delta_{im}}}{1+\mathcal{I}_{\Delta_{im}}}\tau_{im}, \tag{2.57}$$

where $\overline{f(\tau_{im})}$ is the average value of $f(\tau_{im},J_j)=\frac{1-\frac{e^{-\frac{\tau_{im}}{J_j}}}{1-\eta}}{\frac{\tau_{im}}{J_j}+\ln(1-\eta)}$ across all nodes j being part of triangles. As $\overline{f(\tau_{im})}$ is too complex to be expressed by the series of J_j and τ_{im}, we consider two situations and denote f as $\overline{f(\tau_{im})}$ to classify two situations as follows:

① $\frac{\tau_{im}}{J_j}\to-\ln(1-\eta)$, i.e., $f\to1$. In this case, the value of x_i is similar with x_j, and we can obtain the propagation time as

$$\tau_{im}=-\ln(1-\eta)J_i(1+\mathcal{I}_{\Delta_{im}}). \tag{2.58}$$

If we take $d_i\to\infty$, the propagation time becomes independent of m and will be mainly determined by $d_i^{\theta_J+1}$, i.e.,

$$\tau_i\sim d_i^{\theta_J+1}. \tag{2.59}$$

② $\frac{\tau_{im}}{J_j}\not\to-\ln(1-\eta)$, i.e., $f<1$. This situation represents the value of x_i is much smaller or much larger than x_j, and we can obtain the propagation time as

$$\tau_{im}=-\ln(1-\eta)J_i\frac{1+\mathcal{I}_{\Delta_{im}}}{1+(1-f)\mathcal{I}_{\Delta_{im}}}. \tag{2.60}$$

Note that in the manuscript, we use the notation $\mathcal{C}$ to replace $\mathcal{I}$. If we take $d_i\to\infty$, the propagation time becomes independent of m and will be mainly determined by $d_i^{\theta_J}$, i.e.,

$$\tau_i\sim d_i^{\theta_J}. \tag{2.61}$$

2.3.4 Brief Summary for Independent Edges

To sum up, we integrate impacts from three factors, to predict comprehensive dynamic outcomes and classify distinctive dynamic patterns. We investigate combined action between J_i and the effect of independent edges first, that is, exploring the trend of $J_i/\left(1-c(\eta)\mathcal{I}_{L_{im}}\right)$ with the variation of d_i, where $c(\eta)=-\frac{1}{\ln(1-\eta)}\frac{\eta}{1-\eta}$. For simplification, we solve

$$\frac{\mathrm{d}\ln\left(J_i/\left(1-c(\eta)\mathcal{I}_{L_{im}}\right)\right)}{\mathrm{d}\ln d_i}=-\left[\frac{1}{J_i}\left(1-c(\eta)\mathcal{I}_{L_{im}}\right)\right]'\frac{d_i}{\frac{1}{J_i}\left(1-c(\eta)\mathcal{I}_{L_{im}}\right)}. \tag{2.62}$$

Based on this equation, we consider the case when degree is large enough first. Signature of θ_J decides three cases of J_i's variation, and same as θ_Q, three cases of $\mathcal{I}_{L_{im}}$'s trends are confirmed. Pair−wise combinations generate nine cases totally in Table. S3. There are seven cases labelled (1) easily to be drawn so we mainly focus pairs with label (2). For explaining the results, we take the expressions characterizing leading terms into the combined pairs

$$\frac{1}{J_i}\left(1-c(\eta)\mathcal{I}_{L_{im}}\right)\approx\frac{1}{U_\infty}d_i^{-\theta_J}\left(1-c(\eta)V_\infty d_i^{\theta_Q}\overline{\mathcal{Q}}_L\right). \tag{2.63}$$

Differentiate the equation and get

$$\left[\frac{1}{J_i}\left(1-c(\eta)\mathcal{I}_{L_{im}}\right)\right]' \approx -\theta_J \frac{1}{U_\infty} d_i^{-\theta_J-1} + (\theta_J-\theta_Q)\,\frac{V_\infty}{U_\infty} c(\eta) d_i^{-\theta_J+\theta_Q-1}\overline{\mathcal{Q}}_L. \tag{2.64}$$

Hence, the discriminant is transformed into

$$\frac{\mathrm{d}\ln\left(J_i/\left(1-c(\eta)\mathcal{I}_{L_{im}}\right)\right)}{\mathrm{d}\ln d_i} = \frac{c(\eta)\theta_Q V_\infty d_i^{\theta_Q}\overline{\mathcal{Q}}_L}{1-c(\eta)V_\infty d_i^{\theta_Q}\overline{\mathcal{Q}}_L} + \theta_J. \tag{2.65}$$

Based on this discriminant, we discuss about two cases which are hard to judge.

(a) When J_i is positively associated with d_i, i.e., $\theta_J > 0$ and $1/(1-c(\eta)\mathcal{I}_{L_{im}})$ negatively associated with d_i, i.e., $\theta_Q < 0$, there exists $\lim_{d_i\to\infty} d_i^{\theta_Q} = 0$. At that moment, $\frac{\mathrm{d}\ln(J_i/(1-c(\eta)\mathcal{I}_{L_{im}}))}{\mathrm{d}\ln d_i} = \theta_J$. Thus, the combined pairs exhibit positive correlations.

(b) When J_i is negatively associated with d_i, i.e., $\theta_J < 0$ and $1/(1-c(\eta)\mathcal{I}_{L_{im}})$ positively associated with d_i, i.e., $\theta_Q > 0$, there holds $\frac{\mathrm{d}\ln(J_i/(1-c(\eta)\mathcal{I}_{L_{im}}))}{\mathrm{d}\ln d_i} = \theta_J - \theta_Q$. Thus, the combined pairs present negative trends.

J_i / $1/(1-c(\eta)\mathcal{I}_{L_{im}})$	Positive /	Unrelated —	Negative \
Positive /	Positive$^{(1)}$ /	Positive$^{(1)}$ /	Negative$^{(2)}$ \
Unrelated —	Positive$^{(1)}$ /	Unrelated$^{(1)}$ —	Negative$^{(1)}$ \
Negative \	Positive$^{(2)}$ /	Negative$^{(1)}$ \	Negative$^{(1)}$ \

Table S3: Trends of combined pairs with large degree $d_i \to \infty$.

After clearing out the situation when $d_i \to \infty$, we then investigate the case when $d_i \to 1$. Substitute each Taylor expansion into the combination, and derive

$$\frac{1}{J_i}\left(1-c(\eta)\mathcal{I}_{L_{im}}\right) = \frac{1-c(\eta)\sum_{\ell=0}^{\infty} V_\ell (d_i-1)^{\ell+1}\overline{\mathcal{Q}}_L}{\sum_{k=0}^{\infty} U_k (d_i-1)^k}. \tag{2.66}$$

The corresponding derivative becomes

$$\begin{aligned}\left[\frac{1}{J_i}\left(1-c(\eta)\mathcal{I}_{L_{im}}\right)\right]' = &-\frac{c(\eta)\sum_{k=0}^{\infty} U_k(d_i-1)^k \sum_{\ell=0}^{\infty}(\ell+1)V_\ell(d_i-1)^\ell \overline{\mathcal{Q}}_L}{\left(\sum_{k=0}^{\infty} U_k(d_i-1)^k\right)^2} \\ &-\frac{\left(1-c(\eta)\sum_{\ell=0}^{\infty} V_\ell(d_i-1)^{\ell+1}\overline{\mathcal{Q}}_L\right)\sum_{k=0}^{\infty} kU_k(d_i-1)^{k-1}}{\left(\sum_{k=0}^{\infty} U_k(d_i-1)^k\right)^2}.\end{aligned} \tag{2.67}$$

As $d_i \to 1$, the discriminant is simplified as

$$\lim_{d_i\to 1}\frac{\mathrm{d}\ln\left(J_i/\left(1-c(\eta)\mathcal{I}_{L_{im}}\right)\right)}{\mathrm{d}\ln d_i} = \frac{U_1}{U_0} + c(\eta)V_0\overline{\mathcal{Q}}_L, \tag{2.68}$$

where U_0 is negative while $c(\eta)V_0\overline{\mathcal{Q}}_L$ is positive. Trends with label (1) are obvious but the trends labelled (2) are based on signature of the discriminant Eq. (2.68).

J_i / $1/(1-c(\eta)\mathcal{I}_{L_{im}})$	Positive /	Unrelated —	Negative \	
Positive /	Positive[(1)] /	Positive[(1)] /	Positive[(2)] /	Negative[(2)] \

Table S4: Trends of combined pairs with small degree $d_i \to 1$.

Now we piece together all cases under two extreme cases. For displaying combined results clearly, we select same trends of J_i in both two cases, i.e., increase, decrease and constant as these three trends appear in most models. Then we integrate the trends and draw the result Table.

J_i / $1/(1-c(\eta)\mathcal{I}_{L_{im}})$	Positive /	Unrelated —	Negative \	
Positive & Positive /…/	Positive & Positive /…/	Positive & Positive /…/	Positive & Negative /…\	Negative & Negative \…\
Positive & Unrelated /…—		Positive & Unrelated /…—		
Positive & Negative /…\		Positive & Negative /…\		

Table S5: Trends of combined pairs integrating predictions of Tables S3 and S4.

In the former subsection we have mentioned that effect of triangles is determined by $\overline{\mathcal{Q}}_\Delta$ and this mean value depends on the network under given dynamic model. Therefore, we will illustrate the detailed final dynamic outcomes according to the given dynamic models in the following context.

3 Global Propagation Time

In the former section, we have established the general theoretical framework to quantify the propagation time from source node to its adjacent nodes, revealing effects of topological properties, including triangles and independent edges, from a local perspective. Ulteriorly, we further track the signal flow reaching other distant nodes and quantify propagation time from a global perspective.

3.1 Quantification of $T(m \to i)$

We aim at investigating how signal perturbations propagate to distant nodes. To quantify geographical distances from node m, we set i_k as the node occupying the k−th layer, e.g., i_1 means adjacent node of m, and the propagation time $T(m \to i_1)$ is equivalent to τ_{im} in the local case. By employing amended Gauss iterative method, the quantification for the propagation time arriving at a node in the k−th ($k > 1$) layer becomes

$$T(m \to i_k) = T(m \to i_{k-1}) - \frac{\Delta x_{i_k}\big(T(m \to i_{k-1})\big) - \eta \Delta x_{i_k}(\infty)}{\Delta \dot{x}_{i_k}\big(T(m \to i_{k-1})\big) - \eta \Delta \dot{x}_{i_k}(\infty)}. \tag{3.1}$$

We provide the feasibility of Eq. (3.1) in *Methods* and obtain $\Delta \dot{x}_{i_k}\big(T(m \to i_{k-1})\big)$

$$\begin{aligned}\Delta \dot{x}_{i_k}(T(m \to i_{k-1})) = &-\frac{1}{J_{i_k}} \Delta x_{i_k}(T(m \to i_{k-1})) + H_1(x^*_{i_k}) \sum_{\substack{j=1 \\ j \neq i_{k-1}}}^{N} A_{i_k j} H_2^{'}(x^*_j) \Delta x_j(T(m \to i_{k-1})) \\ &+ A_{i_k i_{k-1}} H_1(x^*_{i_k}) H_2^{'}(x^*_{i_{k-1}}) \eta \Delta x_{i_{k-1}}(\infty).\end{aligned} \tag{3.2}$$

When $t \to \infty$, we have

$$0 = -\frac{1}{J_{i_k}} \Delta x_{i_k}(\infty) + H_1(x^*_{i_k}) \sum_{\substack{j=1 \\ j \neq i_{k-1}}}^{N} A_{i_k j} H_2^{'}(x^*_j) \Delta x_j(\infty) + A_{i_k i_{k-1}} H_1(x^*_{i_k}) H_2^{'}(x^*_{i_{k-1}}) \Delta x_{i_{k-1}}(\infty). \tag{3.3}$$

Through subtraction, the expression of $\Delta \dot{x}_{i_k}\big(T(m \to i_{k-1})\big)$ becomes

$$\Delta \dot{x}_{i_k}(T(m \to i_{k-1})) = \frac{1}{J_{i_k}} \left(-1 + \mathcal{E}_{i_k i_{k-1}}(T(m \to i_{k-1}))\right) \left(\Delta x_{i_k}(T(m \to i_{k-1})) - \eta \Delta x_{i_k}(\infty)\right), \tag{3.4}$$

in which

$$\mathcal{E}_{i_k i_{k-1}}(T(m \to i_{k-1})) = J_{i_k} H_1(x^*_{i_k}) \sum_{\substack{j=1 \\ j \neq i_{k-1}}}^{N} A_{i_k j} H_2^{'}(x^*_j) \delta_{i_k j}(T(m \to i_{k-1})), \tag{3.5}$$

and

$$\delta_{ij}(T(m \to i_{k-1})) = \frac{\Delta x_j(T(m \to i_{k-1})) - \eta \Delta x_j(\infty)}{\Delta x_i(T(m \to i_{k-1})) - \eta \Delta x_i(\infty)}. \tag{3.6}$$

Substituted Eq. (3.4) into Eq. (3.1), the quantification of $T(m \to i_k)$ is

$$T(m \to i_k) = T(m \to i_{k-1}) + J_{i_k} \frac{1}{1 - \mathcal{E}_{i_k i_{k-1}}(T(m \to i_{k-1}))}. \tag{3.7}$$

Similar with the quantification for local case, J_{i_k} refers to i_k's intrinsic dynamics and $\mathcal{E}_{i_k i_{k-1}}(T(m \to i_{k-1}))$ represents effects from i_k's neighbors. The iterative expression characterizing propagation time reaching i_k depends on the time reaching i_{k-1}, and propagation time for the signal penetrating through any layers can be quantified after iterations.

3.2 Structural Split of $\mathcal{E}_{i_k i_{k-1}}(T(m \to i_{k-1}))$

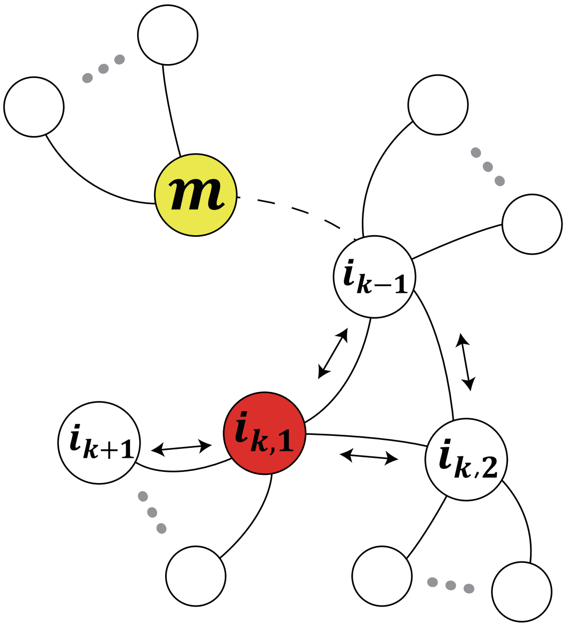

Figure S2: Schematic diagram of signal propagation flowing from a source towards distant nodes in $k-$th layer. Yellow source node m is perturbed. We focus on the response of red target node $i_{k,1}$ in $k-$th layer. Node i_{k+1} is adjacent to target $i_{k,1}$ but not to i_{k-1}, and is taken a contribution to an independent edge. Nodes $i_{k,1}$, i_{k-1} and $i_{k,2}$ form a triangle. The directional arrow expresses direction of the signal propagation.

Similar with the local case, both triangles and independent edges could play an important role along the non-local signal propagation as illustrated in Fig. S2. We firstly simplify $\delta_{ij}(T(m \to i_{k-1}))$ as

$$\delta_{ij}(T(m \to i_{k-1})) = \frac{\frac{\Delta x_j(T(m \to i_{k-1}))}{\Delta x_j(\infty)} - \eta}{\frac{\Delta x_i(T(m \to i_{k-1}))}{\Delta x_i(\infty)} - \eta} \frac{\Delta x_j(\infty)}{\Delta x_i(\infty)}. \tag{3.8}$$

We suppose that the relative response $\frac{\Delta x_j(T(m \to i_{k-1}))}{\Delta x_j(T(m \to j))}$ is equivalent to the division of time $\frac{T(m \to i_{k-1})}{T(m \to j)}$, and $\frac{\Delta x_i(T(m \to i_{k-1}))}{\Delta x_i(T(m \to i))}$ is equivalent to the division of time $\frac{T(m \to i_{k-1})}{T(m \to i)}$. Substitute $\Delta x_j(\infty)$ and $\Delta x_i(\infty)$ into Eq. (3.8) with $\eta \Delta x_j(\infty) = \Delta x_j(T(m \to j))$ and $\eta \Delta x_i(\infty) = \Delta x_i(T(m \to i))$, and obtain

$$\delta_{ij}(T(m \to i_{k-1})) = \frac{\frac{1}{T(m \to i_{k-1})} - \frac{1}{T(m \to j)}}{\frac{1}{T(m \to i_{k-1})} - \frac{1}{T(m \to i)}} \delta_{ij}(0). \tag{3.9}$$

Additionally, we provide another way to simplify the equation based on a different assumption and derive distinct expressions (see *Methods*). Based on that, we quantify the signal propagation in terms of independent edges and triangles

$$\begin{aligned} \mathcal{E}_{i_k i_{k-1}}(T(m \to i_{k-1})) = {} & J_{i_k} H_1(x^*_{i_k}) \sum_{j \in L_{i_k i_{k-1}}} A_{i_k j} H_2'(x^*_j) \frac{\frac{1}{T(m \to i_{k-1})} - \frac{1}{T(m \to j)}}{\frac{1}{T(m \to i_{k-1})} - \frac{1}{T(m \to i_k)}} \delta_{i_k j}(0) \\ & + J_{i_k} H_1(x^*_{i_k}) \sum_{j \in \Delta_{i_k i_{k-1}}} A_{i_k j} H_2'(x^*_j) \frac{\frac{1}{T(m \to i_{k-1})} - \frac{1}{T(m \to j)}}{\frac{1}{T(m \to i_{k-1})} - \frac{1}{T(m \to i_k)}} \delta_{i_k j}(0). \end{aligned} \tag{3.10}$$

If node j together with nodes i_k and i_{k-1} form an independent edge, $\delta_{i_k j}(0)$ holds

$$\delta_{i_k j}(0) \approx \frac{A_{j i_k} J_j H_1(x_j^*) H_2^{'}(x_{i_k}^*)}{1-\mathcal{E}_{j i_k}(T(m \to i_{k-1}))}. \tag{3.11}$$

If node j is included in a triangle structure, $\delta_{i_k j}(0)$ holds

$$\delta_{i_k j}(0) \approx \frac{A_{j i_{k-1}} J_j H_1(x_j^*) H_2^{'}(x_{i_{k-1}}^*)}{1-\mathcal{E}_{j i_{k-1}}(T(m \to i_{k-1}))} \frac{1-\mathcal{E}_{i_k i_{k-1}}(T(m \to i_{k-1}))}{A_{i_k i_{k-1}} J_{i_k} H_1(x_{i_k}^*) H_2^{'}(x_{i_{k-1}}^*)}. \tag{3.12}$$

Combining Eqs. (3.11) and (3.12), we obtain

$$\mathcal{E}_{i_k i_{k-1}}(T(m \to i_{k-1})) = 1 - \frac{1-\widetilde{\mathcal{E}}_{L_{i_k i_{k-1}}}}{1+\widetilde{\mathcal{E}}_{\Delta_{i_k i_{k-1}}}}, \tag{3.13}$$

where effects of triangles $\widetilde{\mathcal{E}}_{\Delta_{i_k i_{k-1}}}$ and independent edges $\widetilde{\mathcal{E}}_{L_{i_k i_{k-1}}}$ in global case are quantified as

$$\begin{cases} \widetilde{\mathcal{E}}_{\Delta_{i_k i_{k-1}}} = \sum_{j \in \Delta_{i_k i_{k-1}}} \frac{A_{i_k j} A_{j i_{k-1}}}{A_{i_k i_{k-1}}} J_j H_1(x_j^*) H_2^{'}(x_j^*) \frac{\frac{1}{T(m\to i_{k-1})} - \frac{1}{T(m \to j)}}{\frac{1}{T(m\to i_{k-1})} - \frac{1}{T(m\to i_k)}} \frac{1}{1-\mathcal{E}_{j i_{k-1}}(T(m \to i_{k-1}))}, \\ \widetilde{\mathcal{E}}_{L_{i_k i_{k-1}}} = J_{i_k} H_1(x_{i_k}^*) H_2^{'}(x_{i_k}^*) \sum_{j \in L_{i_k i_{k-1}}} A_{i_k j}^2 J_j H_1(x_j^*) H_2^{'}(x_j^*) \frac{\frac{1}{T(m\to i_{k-1})} - \frac{1}{T(m \to j)}}{\frac{1}{T(m\to i_{k-1})} - \frac{1}{T(m\to i_k)}} \frac{1}{1-\mathcal{E}_{j i_k}(T(m \to i_{k-1}))}. \end{cases} \tag{3.14}$$

Therefore, we obtain the iterative propagation time for k−th layer as

$$T(m \to i_k) = T(m \to i_{k-1}) + J_{i_k} \frac{1+\widetilde{\mathcal{E}}_{\Delta_{i_k i_{k-1}}}}{1-\widetilde{\mathcal{E}}_{L_{i_k i_{k-1}}}}. \tag{3.15}$$

From a global perspective, we find that the propagation time is determined by four factors: The first factor is intrinsic dynamics, i.e., J_{i_k}; the second is effect of independent edges, i.e., $\widetilde{\mathcal{E}}_{L_{i_k i_{k-1}}}$; the third is effect of triangles, i.e., $\widetilde{\mathcal{E}}_{\Delta_{i_k i_{k-1}}}$; the fourth is the length of chain, i.e., k. If we neglect interactions with the target node, i.e., the effects of independent edges and triangles, it yields

$$T(m \to i_k) = T(m \to i_{k-1}) + J_{i_k}, \tag{3.16}$$

and this matches the propagation time in local case.

In the case of independent edges, j is regarded as i_{k+1}, and then the form of this equation will be

$$\frac{\frac{1}{T(m\to i_{k-1})} - \frac{1}{T(m\to j)}}{\frac{1}{T(m\to i_{k-1})} - \frac{1}{T(m\to i_k)}} = \frac{1-\frac{T(m\to i_{k-1})}{T(m\to i_{k+1})}}{1-\frac{T(m\to i_{k-1})}{T(m\to i_k)}} = \frac{1+\frac{T(m\to i_{k+1})-T(m\to i_k)}{T(m\to i_k)-T(m\to i_{k-1})}}{1+\frac{T(m\to i_{k+1})-T(m\to i_k)}{T(m\to i_k)}}. \tag{3.17}$$

Substituting Eq. (3.16) into this equation approximates

$$\frac{1+\frac{T(m\to i_{k+1})-T(m\to i_k)}{T(m\to i_k)-T(m\to i_{k-1})}}{1+\frac{T(m\to i_{k+1})-T(m\to i_k)}{T(m\to i_k)}} \approx 1 + \frac{J_{i_{k+1}}}{J_{i_k}}. \tag{3.18}$$

In the case of triangles, j is regarded as $i_{k,2}$ and i_k is seen as $i_{k,1}$ for distinction. Then the form of this equation is split as

$$\frac{\frac{1}{T(m\to i_{k-1})} - \frac{1}{T(m\to j)}}{\frac{1}{T(m\to i_{k-1})} - \frac{1}{T(m\to i_k)}} = \frac{1-\frac{T(m\to i_{k-1})}{T(m\to i_{k,2})}}{1-\frac{T(m\to i_{k-1})}{T(m\to i_{k,1})}} = \frac{T(m \to i_{k,2}) - T(m \to i_{k-1})}{T(m \to i_{k,1}) - T(m \to i_{k-1})} \frac{1+\frac{T(m\to i_{k,1})-T(m\to i_{k-1})}{T(m\to i_{k-1})}}{1+\frac{T(m\to i_{k,2})-T(m\to i_{k-1})}{T(m\to i_{k-1})}}. \tag{3.19}$$

Substituting Eq. (3.16) into this equation approximates

$$\frac{T(m\to i_{k,2})-T(m\to i_{k-1})}{T(m\to i_{k,1})-T(m\to i_{k-1})}\frac{1+\frac{T(m\to i_{k,1})-T(m\to i_{k-1})}{T(m\to i_{k-1})}}{1+\frac{T(m\to i_{k,2})-T(m\to i_{k-1})}{T(m\to i_{k-1})}}\approx\frac{J_{i_{k,2}}}{J_{i_{k,1}}}. \tag{3.20}$$

After neglecting marginal effects $\mathcal{E}_{ji_{k-1}}(T(m\to i_{k-1}))$ and $\mathcal{E}_{ji_k}(T(m\to i_{k-1}))$, and take $Q_i^{(0)}=J_iH_1(x_i^*)H_2^{'}(x_i^*)$ in the local case, we obtain

$$\begin{cases}\widetilde{\mathcal{E}}_{L_{i_ki_{k-1}}}=Q_{i_k}^{(0)}\sum_{j\in L_{i_ki_{k-1}}}A_{i_kj}^2Q_j^{(0)}\frac{\frac{1}{T(m\to i_{k-1})}-\frac{1}{T(m\to j)}}{\frac{1}{T(m\to i_{k-1})}-\frac{1}{T(m\to i_k)}},\\ \widetilde{\mathcal{E}}_{\Delta_{i_ki_{k-1}}}=\sum_{j\in\Delta_{i_ki_{k-1}}}\frac{A_{i_kj}A_{ji_{k-1}}}{A_{i_ki_{k-1}}}Q_j^{(0)}\frac{\frac{1}{T(m\to i_{k-1})}-\frac{1}{T(m\to j)}}{\frac{1}{T(m\to i_{k-1})}-\frac{1}{T(m\to i_k)}}.\end{cases} \tag{3.21}$$

Substituting this equation into Eq. (3.15), we derive

$$T(m\to i_k)-T(m\to i_{k-1})=\frac{\mathcal{F}_{L_{i_ki_{k-1}}}+J_{i_k}(1+\widetilde{\mathcal{E}}_{\Delta_{i_ki_{k-1}}})}{1-\mathcal{G}_{L_{i_ki_{k-1}}}}. \tag{3.22}$$

where

$$\begin{cases}\mathcal{F}_{L_{i_ki_{k-1}}}=Q_{i_k}^{(0)}\sum_{j\in L_{i_ki_{k-1}}}A_{i_kj}^2Q_j^{(0)}(T(m\to j)-T(m\to i_k))\frac{T(m\to i_k)}{T(m\to j)},\\ \mathcal{G}_{L_{i_ki_{k-1}}}=Q_{i_k}^{(0)}\sum_{j\in L_{i_ki_{k-1}}}A_{i_kj}^2Q_j^{(0)}\frac{T(m\to i_k)}{T(m\to j)}.\end{cases} \tag{3.23}$$

Through Eq. (3.23), the relation holds

$$\mathcal{F}_{L_{i_ki_{k-1}}}=T(m\to i_k)(\mathcal{E}_{L_{i_ki_{k-1}}}-\mathcal{G}_{L_{i_ki_{k-1}}}), \tag{3.24}$$

where $\mathcal{E}_{L_{i_ki_{k-1}}}=Q_{i_k}^{(0)}\sum_{j\in L_{i_ki_{k-1}}}A_{i_kj}^2Q_j^{(0)}$, similar to the corresponding term in the local case. Substituting Eq. (3.24) into Eq. (3.22), we obtain the iterative propagation time for k−th layer as

$$T(m\to i_k)=T(m\to i_{k-1})\frac{1-\mathcal{G}_{L_{i_ki_{k-1}}}}{1-\mathcal{E}_{L_{i_ki_{k-1}}}}+\frac{J_{i_k}(1+\widetilde{\mathcal{E}}_{\Delta_{i_ki_{k-1}}})}{1-\mathcal{E}_{L_{i_ki_{k-1}}}}. \tag{3.25}$$

Therefore, the propagation time, from the former layer i_{k-1} to its next one i_k, is a linear relation. $\frac{1-\mathcal{G}_{L_{i_ki_{k-1}}}}{1-\mathcal{E}_{L_{i_ki_{k-1}}}}$ is a linear coefficient and its value is larger than one. With the increase of layers, the linear coefficient $\frac{1-\mathcal{G}_{L_{i_ki_{k-1}}}}{1-\mathcal{E}_{L_{i_ki_{k-1}}}}$ varies, indicating the speed variation of signal spreading.

3.3 Effect of Topological Characteristics

From a global perspective, there are four factors, including intrinsic dynamics, effect of independent edges, effect of triangles and length of chain, contributing to the distant signal propagation. The relationship between node's final state x_i^* and its degree d_i in distant signal propagation remains the same as that in adjacent propagation. We will therein mainly discuss the effects of triangles and degree configurations in this subsection.

For the effect of triangles on each layer, we have

$$\widetilde{\mathcal{E}}_{\Delta_{i_ki_{k-1}}}=\sum_{j\in\Delta_{i_ki_{k-1}}}\frac{A_{i_kj}A_{ji_{k-1}}}{A_{i_ki_{k-1}}}\frac{J_j}{J_{i_k}}Q_j^{(0)}, \tag{3.26}$$

and obtain the mean effects across different layers as follows

$$\mathcal{R}_{\Delta} = \frac{1}{d_{i_{k-1}}} \sum_{i_k \in K_{i_{k-1}}} \frac{1}{t_{i_k i_{k-1}}} \sum_{j \in \Delta_{i_k i_{k-1}}} \frac{A_{i_k j} A_{j i_{k-1}}}{A_{i_k i_{k-1}}} \frac{J_j}{J_{i_k}} Q_j^{(0)}. \tag{3.27}$$

This yields

$$\widetilde{\mathcal{E}}_{\Delta_{i_k i_{k-1}}}(T(m \to i_{k-1})) = t_{i_k i_{k-1}} \mathcal{R}_{\Delta}, \tag{3.28}$$

and trends of $\widetilde{\mathcal{E}}_{\Delta_{i_k i_{k-1}}}(T(m \to i_{k-1}))$ are proportional to the number of triangles $t_{i_k i_{k-1}}$. Consider there are more than one shortest path between nodes, we obtain the distant signal propagation time Γ_{im} from node m to distant node i as

$$\Gamma_{im} = \min_{\Pi(m \to i)} \left\{ \sum_{\substack{q \in \Pi(m \to i) \\ q \neq m}} \left(J_q + J_q t_{i_k i_{k-1}} \mathcal{R}_{\Delta} \right) \right\}, \tag{3.29}$$

where $\Pi(m \to i)$ is the set of the shortest paths between nodes m and i.

To further understand how degree configuration conducts propagation time from a global perspective, we investigate the sequence of nodes' degree along the main chain from source m to target i_k. For simplification, consider a chain without triangles, Eq. (3.25) becomes

$$T(m \to i_k) = T(m \to i_{k-1}) \frac{1 - \mathcal{G}_{L_{i_k i_{k-1}}}}{1 - \mathcal{E}_{L_{i_k i_{k-1}}}} + \frac{J_{i_k}}{1 - \mathcal{E}_{L_{i_k i_{k-1}}}}. \tag{3.30}$$

For layer k equal to 1, this quantification characterizes the local case as

$$T(m \to i_1) = -\ln(1-\eta) J_{i_1} \frac{1}{1 - c(\eta) \mathcal{E}_{L_{i_1 m}}}, \tag{3.31}$$

where $c(\eta) = -\frac{1}{\ln(1-\eta)} \frac{\eta}{1-\eta}$. Next, for k equal to 2, substituting Eq. (3.31) into the quantification (3.30) yields

$$\begin{aligned} T(m \to i_2) &= T(m \to i_1) \frac{1 - \mathcal{G}_{L_{i_2 i_1}}}{1 - \mathcal{E}_{L_{i_2 i_1}}} + \frac{J_{i_2}}{1 - \mathcal{E}_{L_{i_2 i_1}}} \\ &= -\ln(1-\eta) J_{i_1} \frac{1}{1 - c(\eta) \mathcal{E}_{L_{i_1 m}}} \cdot \frac{1 - \mathcal{G}_{L_{i_2 i_1}}}{1 - \mathcal{E}_{L_{i_2 i_1}}} + \frac{J_{i_2}}{1 - \mathcal{E}_{L_{i_2 i_1}}}. \end{aligned} \tag{3.32}$$

From this recursion, we conclude that

$$T(m \to i_k) = \sum_{h=1}^{k} \frac{f(h) J_{i_h}}{1 - k(h) \mathcal{E}_{L_{i_h i_{h-1}}}} \prod_{j=h+1}^{k} \left(\frac{1 - \mathcal{G}_{L_{i_j i_{j-1}}}}{1 - \mathcal{E}_{L_{i_j i_{j-1}}}} \right), \tag{3.33}$$

where we set $\prod_{j=k+1}^{k} \left(1 - \mathcal{G}_{L_{i_j i_{j-1}}}\right) = 1$ for simplification,

$$f(h) = \begin{cases} -\ln(1-\eta), h = 1, \\ 1, h > 1, \end{cases} \tag{3.34}$$

and

$$k(h) = \begin{cases} c(\eta), h = 1, \\ 1, h > 1. \end{cases} \tag{3.35}$$

The effect of independent edges characterizing adjacencies of nodes in each layers follows (see Eq. (2.47))

$$\mathcal{E}_{L_{i_k i_{k-1}}} = Q_{i_k}^{(0)} \sum_{j \in L_{i_k i_{k-1}}} A_{i_k j}^2 Q_j^{(0)} \approx V_\infty d_{i_k}^{\theta_Q} \overline{\mathcal{Q}}_L, \tag{3.36}$$

and intrinsic dynamics is represented as (see Eq. (2.40))

$$J_{i_k} = -1/\left(H_1(x_{i_k}^*) \left[\frac{F(x_{i_k}^*)}{H_1(x_{i_k}^*)} \right]' \right) \approx U_\infty d_{i_k}^{\theta_J}. \tag{3.37}$$

The term $\mathcal{G}_{L_{i_k i_{k-1}}}$ describing the effect of independent edges in the global case is

$$\mathcal{G}_{L_{i_k i_{k-1}}} = Q_{i_k}^{(0)} \sum_{j \in L_{i_k i_{k-1}}} A_{i_k j}^2 Q_j^{(0)} \frac{T\,(m \to i_k)}{T(m \to j)}. \tag{3.38}$$

Substituting Eqs. (3.36) and (3.37) into Eq. (3.33) yields

$$T\,(m \to i_k) = \sum_{h=1}^{k} \frac{f(h) g(h) C_1 d_{i_h}^{\theta_J}}{1 - k(h) C_2 d_{i_h}^{\theta_Q}}, \tag{3.39}$$

where

$$g(h) = \prod_{j=h+1}^{k} \left(\frac{1 - \mathcal{G}_{L_{i_j i_{j-1}}}}{1 - \mathcal{E}_{L_{i_j i_{j-1}}}} \right). \tag{3.40}$$

$g(h)$ is a monotonic decreasing function which is greater than 1, and it tends to be 1 when h increases. We can further simplify the corresponding adjacent dynamics on each layer and obtain

$$g(h) \sim \prod_{j=h+1}^{k} \left(\frac{1}{1 - \mathcal{E}_{L_{i_j i_{j-1}}}} \right). \tag{3.41}$$

Additionally, when $h = k$, $g(h)$ is set as 1. Two positive terms are

$$C_1 = U_\infty, C_2 = V_\infty \overline{\mathcal{Q}}_L. \tag{3.42}$$

Given a dynamic model but with different configurations of degree, the quantification of propagation time arriving at i_k can be treated as a multivariate function whose parameters are class of degrees of each layer. We obtain the propagation time (3.39) along a chain as

$$T(m \to i_k) = F\left(d_{i_1} \cdots d_{i_k}\right) = \left(f(1)g(1)C_1, ..., f(k)g(k)C_1\right) \begin{pmatrix} \frac{d_{i_1}^{\theta_J}}{1 - c(\eta) C_2 d_{i_1}^{\theta_Q}} \\ \vdots \\ \frac{d_{i_k}^{\theta_J}}{1 - C_2 d_{i_k}^{\theta_Q}} \end{pmatrix}. \tag{3.43}$$

The time function is determined by the multiplication of two vectors.

$$F\left(d_{i_1} \cdots d_{i_k}\right) \sim \left(g(1), g(2), ..., g(k-1), g(k)\right) \begin{pmatrix} -\ln(1-\eta) \frac{d_{i_1}^{\theta_J}}{1 - c(\eta) C_2 d_{i_1}^{\theta_Q}} \\ \frac{d_{i_2}^{\theta_J}}{1 - C_2 d_{i_2}^{\theta_Q}} \\ \vdots \\ \frac{d_{i_{k-1}}^{\theta_J}}{1 - C_2 d_{i_{k-1}}^{\theta_Q}} \\ \frac{d_{i_k}^{\theta_J}}{1 - C_2 d_{i_k}^{\theta_Q}} \end{pmatrix}, \tag{3.44}$$

which can be simplified as

$$F\left(d_{i_1}\cdots d_{i_k}\right)\sim \mathbf{g}^T\mathbf{D}, \tag{3.45}$$

i.e., $\mathbf{g}^T=(g(1),g(2),...,g(k-1),g(k))$ and

$$\mathbf{D}=\mathbf{D}\left(d_{i_1}\cdots d_{i_k}\right)=\begin{pmatrix} -\ln(1-\eta)\frac{d_{i_1}^{\theta_J}}{1-c(\eta)C_2d_{i_1}^{\theta_Q}} \\ \frac{d_{i_2}^{\theta_J}}{1-C_2d_{i_2}^{\theta_Q}} \\ \vdots \\ \frac{d_{i_{k-1}}^{\theta_J}}{1-C_2d_{i_{k-1}}^{\theta_Q}} \\ \frac{d_{i_k}^{\theta_J}}{1-C_2d_{i_k}^{\theta_Q}} \end{pmatrix}. \tag{3.46}$$

Numerically, we suppose that the vector $\mathbf{g}$ is independent with the degree sequence $(d_{i_1}\cdots d_{i_k})$ but the elements of $\mathbf{g}$ depend on their corresponding layers (Fig. S3). In this situation, we also numerically validate the linear correspondence between the actual value of $g(h)$ (3.40) and the simplified one (3.41). Thus, the vector $\mathbf{g}$ conducts the sequence of degree for nodes occupying the chain, as $g(h)$ can be regarded as the weight of $h-$th term of $\mathbf{D}$, consequently affecting the summation of propagation time penetrating through layers. The vector $\mathbf{g}$ indicates that even if collections of degree are the same, the sequence of degree along the chain might affect the global propagation time $T(m\to i_k)$, which is different from the universal dynamic metric for signal propagation [1], where the global propagation time is only relevant to collections of degree rather than sequences of degree.

As the decreasing vector $\mathbf{g}$ manages the sequence of node's degree, finally generating different spreading time, we aim to derive extremum of time function through artificial adjustments. Consider it as an integer programming problem, and solve the extremum in the real domain first for simplification. Because of the continuity of the objective function, the corresponding degree set in the integer domain of the optimal value is close to that in the real domain or is exactly that under suitable conditions. We compute partial differential of the quantification Eq. (3.39)

$$\frac{\partial F}{\partial d_{i_h}}=\frac{f(h)g(h)C_1\left(C_2k(h)(\theta_Q-\theta_J)d_{i_h}^{\theta_J+\theta_Q-1}+\theta_Jd_{i_h}^{\theta_J-1}\right)}{(1-k(h)C_2d_{i_h}^{\theta_Q})^2}, \tag{3.47}$$

and solve the equation

$$\frac{\partial F}{\partial d_{i_h}}=0,\ h=1,...,k. \tag{3.48}$$

When class of degrees all satisfy

$$d_{i_1}=d_{i_2}=\cdots=d_{i_k}=\left(\frac{\theta_J}{C_2k(h)(\theta_J-\theta_Q)}\right)^{\frac{1}{\theta_Q}}, \tag{3.49}$$

the time function reaches extremum.

To testify the quantification Eq. (3.43), we do simulated experiments which comprise 100 realizations of different degree sets varying with poisson distribution whose parameter (i.e., mean degree) λ is set as 9. In Fig. S4, simulated global propagation time and its re-scaling Eq. (3.43) fit well, presenting

positive correlation under regulatory dynamics for three different groups of parameters. Further, we aim to verify the evaluation of extremum still under regulatory dynamical model for the same selections of parameters. Shown in Fig. S5, the degree set varies with poisson distribution whose parameter λ is set as 9, and each dot represents each realization for the different degree set. Series of dots present rough negative relation, and the dot whose standard variance equals to zero represents the set generated by all same degrees, basically reaching the extremum under all three different selections of parameters.

As the configuration of degree along the chain affects the global propagation time, generating negative relation between time and standard variation of degree set when fixed the mean degree of the set, we further investigate the time variations under different mean values (Fig. S6). After 300 realizations following poisson distribution for each mean value, there generate probability density distributions for $\lambda = 9$ (blue) and $\lambda = 12$ (orange) respectively. Overlaps for distributions indicate that even though the mean value of degrees along the chain is distinctive, the global propagation time could still be the same under proper adjustments of configuration of degrees.

Therefore, both mathematical expectation and standard variation of the degree set affect the global propagation time. Shown in Fig. S7(a-c), we further explore the effects of these two factors at the same time. Still under the regulatory dynamical model with three groups of parameters, realizations of simulated time for different mean degree and standard variances are averaged, shown by shade of red (Fig. S7(a-c)) after normalization. For further visualizing the relation, we select three different standard variances in Fig. S7(a-c), and exhibit the correlations between normalized time and mean degree in Fig. S7(d-f) respectively. For the given dynamical model in Fig. S7(d) and (e), curves for standard variances equalling to 3 (blue), 5 (orange), 7 (green) respectively present positive tendencies roughly, while in (f), trends for three curves are basically negative. In addition, for the same mean degrees, normalized propagation time for different standard variances is different.

Based on these two factors, we evaluate that

$$T(m \to i_k) \sim d_{avg}^{\theta} \tag{3.50}$$

for a chain without considering triangles. d_{avg} refers to the mean degree of the degree set according to nodes along the chain; θ is an exponent, which conducts patterns of global signal spreading. As for Fig. S7(d-f), θ relates with the standard variance of degree set, conducting the correlation relation between time and mean degree.

Further, we investigate degree sequences with large standard variances, i.e, heterogeneous case for degree distribution. According to the universal metric in [1], predicted scaling for a global signal spreading is a summation of accumulated lag time along each path. While a standard variance of a degree sequence is large enough, the leading scaling is $\max(d_i^{\theta_J})$ when $\theta_J > 0$, and is $\min(d_i^{\theta_J})$ when $\theta_J < 0$, because other terms play marginal effect. To verify that, we select regulatory model with two different class of parameters, which correspond to positive θ and negative θ respectively. From Fig. S8, both two cases present roughly linear relations, indicating that the leading scaling captures actual consequences.

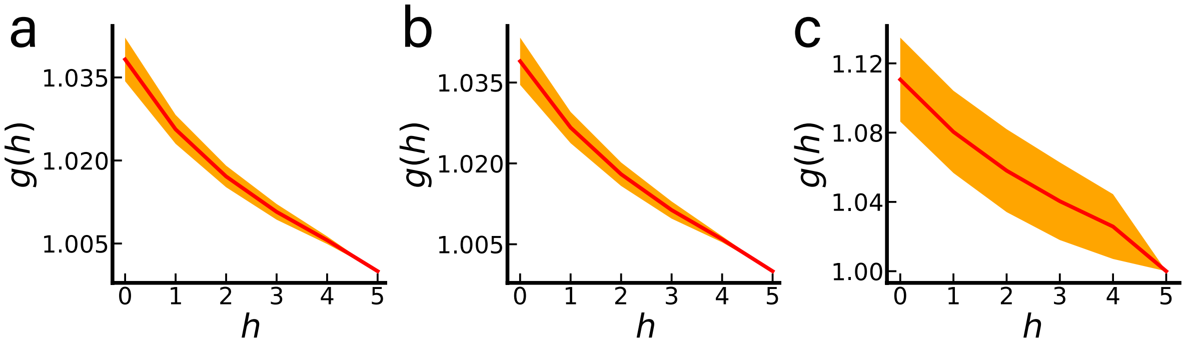

Figure S3: Element $g(h)$ of the vector $\mathbf{g}$ v.s. layer h. The first node in the main chain is set as a source with $\Delta x_m = 0.3$. The length of main chain is set 8. The degree set of other 7 nodes varies according to Poisson distribution with parameter λ equal to 9. The dynamics are regulatory dynamics with (a) $a = 0.8,\ b = 0.5$, (b) $a = 1.0,\ b = 0.5$, (c) $a = 1.2,\ b = 2.0$ respectively, $B = 1$, and $\eta = 0.3$. In all panels, $g(h)$ presents decreasing tendencies and approaches 1 as theory describes. Orange refers 100 numerical realizations, and the red curve refers to the mean value across these simulations. For different degree configurations under the same average degrees with small variations.

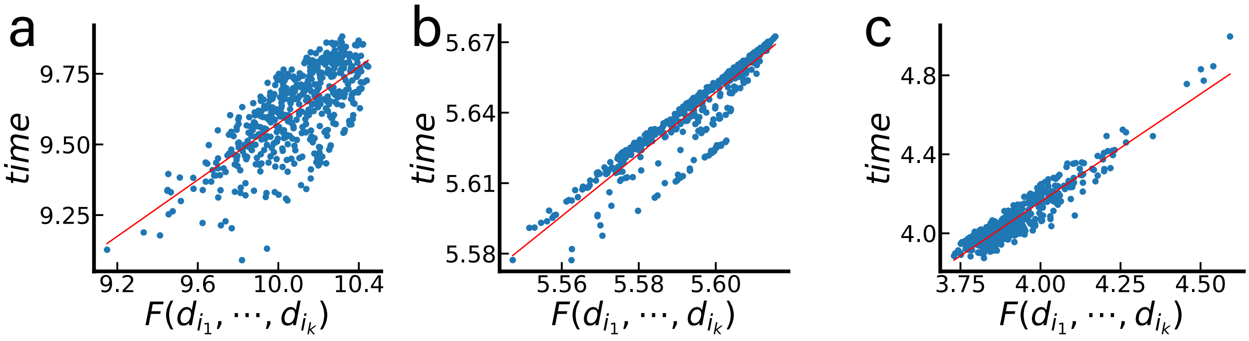

Figure S4: Simulated time v.s. $F(d_{i_1} \cdots d_{i_k})$. The first node in the main chain is set as a source which receives perturbation $\Delta x_m = 0.3$. Keep the first source node constant. The degree set of other 7 nodes varies according to Poisson distribution whose parameter λ is set as 9. Number of simulation experiments is 100, and each dot represents one simulated result. The dynamics are regulatory dynamics whose parameters are set as (a) $a = 0.8,\ b = 0.5$, (b) $a = 1.0,\ b = 0.5$, and (c) $a = 1.2,\ b = 2.0$ respectively. Other parameters are $B = 1,\ \eta = 0.3$. The red curves refer to linear fitting of corresponding dots, and all present increasing tendencies in all three cases.

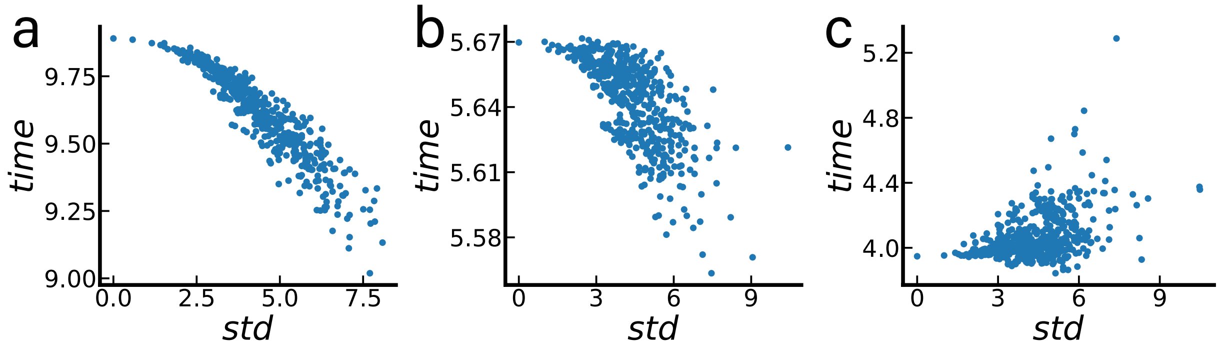

Figure S5: Simulated time v.s. standard deviation of degree set. The first node in the main chain is set as a source which receives perturbation $\Delta x_m = 0.3$. Keep the first source node constant. The degree set of other 7 nodes varies according to Poisson distribution whose parameter λ is set as 9. Number of simulation experiments is 100, and each dot represents one simulated result. The dynamics are regulatory dynamics whose parameters are set as (a) $a = 0.8,\ b = 0.5$, (b) $a = 1.0,\ b = 0.5$, and (c) $a = 1.2,\ b = 2.0$ respectively. Other parameters are $B = 1,\ \eta = 0.3$. Trends of simulated time and standard deviation of degree set present negative correlation in panels (a) and (b), while present roughly positive correlation in (c). When standard deviation of degree set equals to 0, each node has the same degree, and the time reaches an extremum.

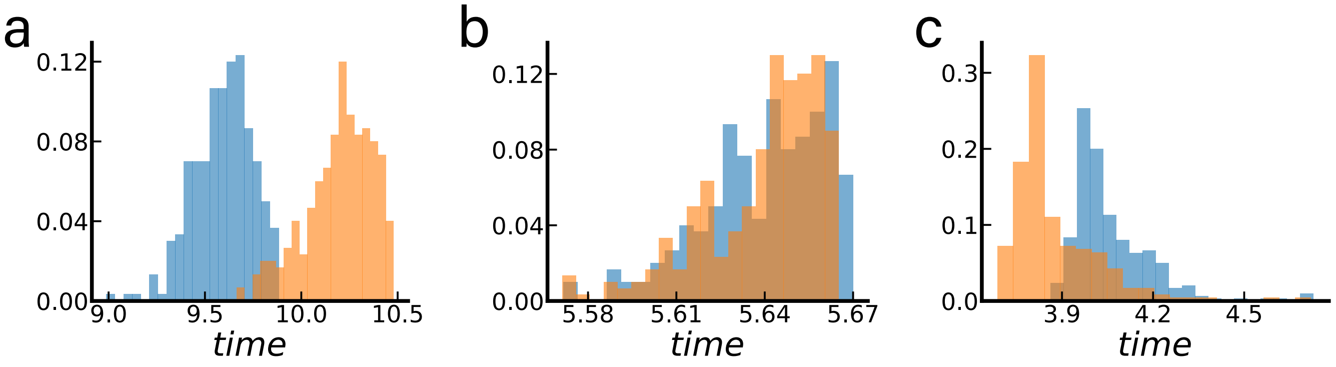

Figure S6: Frequency distribution histogram for degree set according to Poisson distribution with λ equal to 9 (blue) and 12 (orange). Numbers of simulation experiments are set 300, and we count the distribution of simulated time. There are overlaps of two cases, indicating that even with different mean degrees, global propagation time could be the same under proper adjustments of degree configurations.

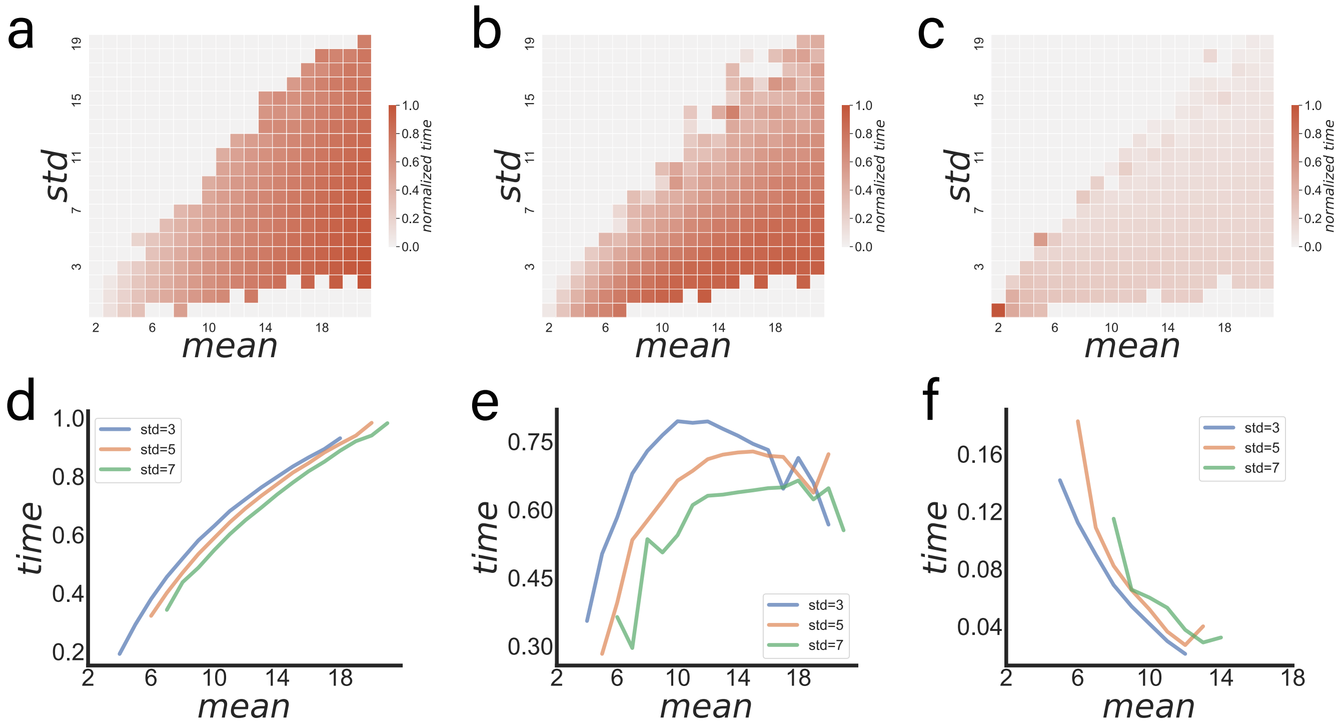

Figure S7: (a-c) Relation between mean degree of degree set, standard deviation of degree set and normalized global propagation time. The degree set varies according to Poisson distribution with λ varying from 2 to 20. For each realization under a given mean degree and a standard deviation, we calculate the corresponding mean normalized propagation time. The shade of red represents the mean value of propagation time after normalization across 500 realizations. (d-f) Normalized propagation time v.s. mean degree under three standard deviations 3 (blue), 5 (orange), and 7 (green). Relation between normalized time and mean degree in panels (d) presents positive correlation, and the curve in (e) increases first and decreases later, while the trend in panel (f) is negative. The dynamics are regulatory dynamics whose parameters are set as (a,d) $a = 0.8,\ b = 0.5$, (b,e) $a = 1.0,\ b = 0.5$, and (c,f) $a = 1.2,\ b = 2.0$ respectively. Other parameters are $B = 1,\ \eta = 0.3$.

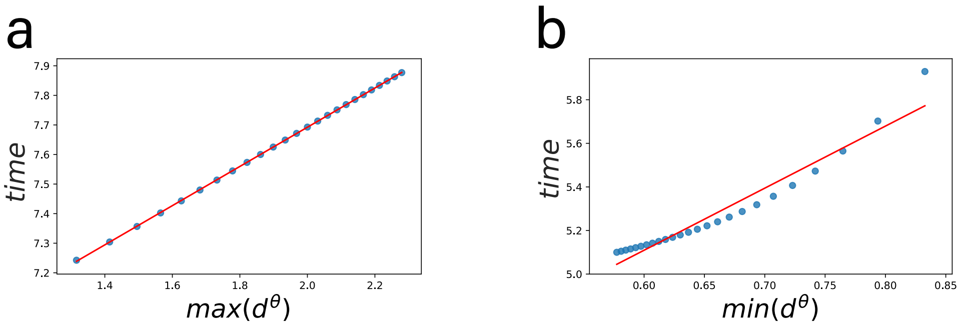

Figure S8: (a) Simulated time v.s. $\max(d^\theta)$ for heterogeneous degree sequences. The dynamics are regulatory dynamics with $a = 0.8$, $b = 0.5$, and we obtain the scaling exponent θ as 0.25. In this case, $\max(d^\theta)$ is k^θ. (b) Simulated time v.s. $\min(d^\theta)$ for heterogeneous degree sequences. The dynamics are regulatory dynamics with $a = 1.2$, $b = 2.0$, and we obtain the scaling exponent θ as -0.17. In this case, $\min(d^\theta)$ is k^θ. The length of main chain is 8. Nodes' degree sequence is $(2, 2, 2, k, 2, 2, 2)$ with respect to layers, and variable k varies from 5 to 30. The linear relationship (red fitting line) for both two cases indicate that the predicted scaling captures the simulated results.

4 Dynamic Models

We have systematically constructed a theoretical framework through quantifying the effects of signal propagation from concrete topological elements, independent edges and triangles, both from local and global perspectives. Combining with dynamical interactions, we condense different dynamical behaviors into detailed distinctive patterns with respect to local nodes' degree. In this section, we implement this framework into commonly−encountered dynamic models, to investigate distinctive effects generated by various topological characteristics under small and large degree, and meanwhile corroborate the rationality of our predictions.

4.1 Regulatory Dynamics

Regulatory network accounts for the gather of molecular species and their interactions, controls gene product abundance, and is crucial for cell differentiation, metabolism, and so on. Investigating the networked dynamics deepers the understanding of the mechanisms of, e.g., diseases with cellular processes dysregulated [7–9]. For further investigation, we take regulatory dynamics, denoted by $\mathbb{R}$, the Michaelis−Menten equation with the following goverying dynamics [8, 10]

$$\dot{x}_i(t) = -Bx_i^a(t) + \alpha \sum_{j=1}^{N} A_{ij} \frac{x_j^b(t)}{1 + x_j^b(t)}, \tag{4.1}$$

where a refers to degradation ($a = 1$) or dimerization ($a = 2$). The specific form of Hill function $\frac{x_j^b(t)}{1+x_j^b(t)}$ describes the activation of x_i by x_j, where b is the Hill coefficient characterizing the level of cooperation in gene regulation [8, 10].

Consider the case of independent edges and based on the derivation in the last section, we obtain key terms as

$$\begin{cases} J_i = \frac{1}{aB}(x_i^*)^{1-a}, \\ Q_i = \frac{\alpha b}{aB} \frac{(x_i^*)^{b-a}}{\left((x_i^*)^b+1\right)^2}, \\ \overline{\mathcal{Q}}_L = \frac{\alpha b}{aB} \frac{1}{d_m} \sum_{i \in K_m} \frac{1}{s_{im}} \sum_{j \in L_{im}} \frac{A_{ij}^2 (x_i^*)^{b-a}}{\left((x_i^*)^b+1\right)^2}, \end{cases} \tag{4.2}$$

and obtain the corresponding propagation time as

$$\tau_i = -\ln(1-\eta) \frac{\frac{1}{aB} \left(x_i^*\right)^{1-a}}{1 + \frac{1}{ln(1-\eta)} \frac{\eta}{1-\eta} \frac{\alpha b}{aB} \frac{(x_i^*)^{b-a}}{\left((x_i^*)^b+1\right)^2} d_i \overline{\mathcal{Q}}_L}. \tag{4.3}$$

Initially, the system is located in the stationary stable regime

$$\begin{cases} x_i^* = \left(\frac{d_i \overline{\mathcal{H}}}{B}\right)^{\frac{1}{a}}, \\ \overline{\mathcal{H}} = \frac{\alpha}{N} \sum_{i=1}^{N} \frac{1}{d_i} \sum_{j=1}^{N} A_{ij} \frac{(x_j^*)^b}{1+(x_j^*)^b}. \end{cases} \tag{4.4}$$

In this case, the key terms can be rewritten based on the degree d_i as

$$\begin{cases} J_i = \frac{1}{aB}\left(\frac{d_i\overline{\mathcal{H}}}{B}\right)^{\frac{1}{a}-1}, \\ Q_i = \frac{\alpha b}{aB}\frac{\left(\frac{d_i\overline{\mathcal{H}}}{B}\right)^{\frac{b-a}{a}}}{\left(\left(\frac{d_i\overline{\mathcal{H}}}{B}\right)^{\frac{b}{a}}+1\right)^2}, \\ \overline{\mathcal{Q}}_L = \frac{b\alpha}{aB}\frac{1}{d_m}\sum_{i\in K_m}\frac{1}{s_{im}}\sum_{j\in L_{im}}\frac{A_{ij}^2\left(\frac{d_j\overline{\mathcal{H}}}{B}\right)^{\frac{b-a}{a}}}{\left(\left(\frac{d_j\overline{\mathcal{H}}}{B}\right)^{\frac{b}{a}}+1\right)^2}. \end{cases} \tag{4.5}$$

When $d_i \to \infty$, we obtain two scaling coefficients $\theta_J = \frac{1}{a} - 1$, $\theta_Q = -\frac{b}{a}$ through Eq. (4.5). a is often set to be 1 and 2 referring to degradation and dimerization respectively. As the Hill coefficient b is always positive, such as the transcriptional regulatory network of Saccharomyces cerevisiae with $B = 1, a = 1$ and $b = 2$ [11], in most cases it holds $\theta_Q < 0$. This yields the propagation time

$$\tau_{im} \sim d_i^{\frac{1}{a}-1}. \tag{4.6}$$

When $f \ll 1$ or the target node i's adjacency dynamics is sufficient large, the influence of triangles on the scaling coefficient can be neglected. When $f \approx 1$, the influence of triangle is apparent, and the scaling will be plus one. Therefore, the propagation time approaches

$$\tau_{im} \sim \begin{cases} d_i^{\frac{1}{a}-1}, f \ll 1, \\ d_i^{\frac{1}{a}}, f \approx 1. \end{cases} \tag{4.7}$$

Additionally, we consider one complicated but normal situation considering both independent edges and triangles coexisting. In particular for each realization, we randomly divide nodes' edges into triangles or independent edges, and evaluate the corresponding response time. As shown in Fig. S10, green dots indicate the evaluated time of various realizations, and these dots are located between two boundaries between all independent edges and all triangles.

For the global case, we consider a protein−protein interaction (PPI) network [12] to investigate the effect of triangles in global case. We show the signal propagation snapshots of PPI network in Fig. S12(a) with $a = 0.8$ and $b = 0.5$. Central node is the source node inducing a perturbation. Nodes in the same layer (same radial distance) have same shortest path length to the source. Yellow nodes indicate the arrival of the perturbation in (an arbitrarily) fixed time with $\eta = 0.3$. Average propagation time as a function of number of triangles and layers in (a) is shown in Fig. S12(b). In this setting, for a given layer, triangles slow propagation. Average propagation time increases with both number of triangles and layers. (c) Signal propagation in rewired networks with varying the average clustering coefficient across nodes by edge rewiring. For all three networks, triangles slow the spreading of the perturbation across layers. Additionally, we investigate the other case with $a = 1.2$ and $b = 2.0$. We show the corresponding signal propagation snapshots of PPI network in Fig. S13(a) and average propagation time as a function of number of triangles and layers in Fig. S13(b). Average propagation time decreases with number of triangles but increases with layers. We show that signal propagation in rewired networks with varying

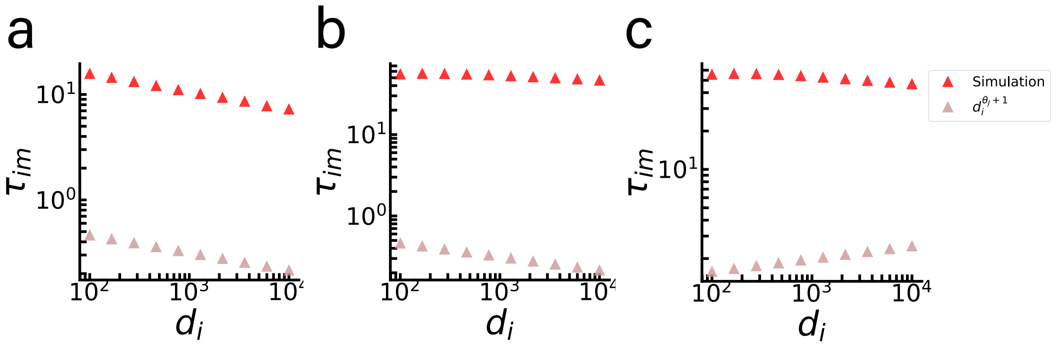

Figure S9: (a) Propagation time τ_{im} v.s. degree d_i comparing simulation with theory under the situation of independent edges with large degree. The scale between the propagation time τ_{im} and the degree d_i is depicted, and the scaling exponent is θ_J. Theoretical propagation time is based on Eq. (4.6). (b-c) Propagation time τ_{im} v.s. degree d_i under the situation of triangles with large degree but in different parameter regime. The scale between the propagation time τ_{im} and the number of triangles (also degree here) d_i is depicted, and the scaling exponent is θ_J in (b), $\theta_J + 1$ in (c). Theoretical propagation time is provided by Eq. (4.7) in (b) and by Eq. (4.7) in (c). Corresponding dynamics are regulatory dynamics, in which $B = 0.01, \alpha = 0.01$ $a = 1.2$, $b = 0.5$ in (a) and (b), and $a = 10.0$, $b = 2.0$ in (c). Fraction η is set as 0.3.

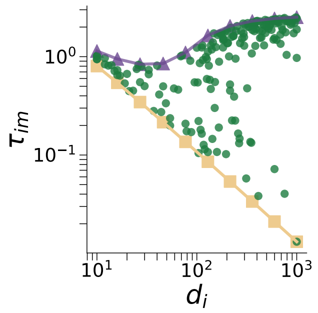

Figure S10: Propagation time τ_{im} v.s. degree d_i considering the co-existing of both independent edges and triangles. There are two boundaries: the upper one corresponding to the case with all triangles (in purple), and the lower one corresponding to the case with all independent edges (in yellow). Green dots represent realizations for both independent edges and triangles coexisting with random partition for fixed degree, ranging from 10 to 1000. There are in total 150 realizations. Corresponding dynamics are regulatory dynamics, in which $B = 1, \alpha = 0.1, a = 10$, and $b = 2$. Fraction η is set as 0.3.

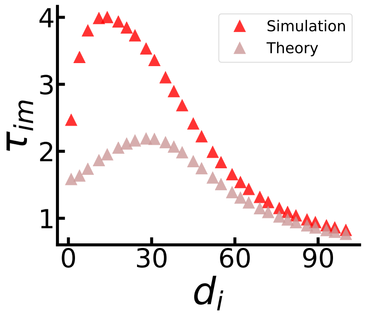

Figure S11: Propagation time τ_{im} v.s. degree d_i comparing simulation with theory under the situation of independent edges with small degree. The relation between the propagation time τ_{im} and the degree d_i is depicted, and the tendency to increase first and then decrease follows the corresponding prediction in Table S5. Theoretical propagation time is based on Eq. (4.3). Corresponding dynamics are regulatory dynamics, in which $B = 1, \alpha = 0.1, a = 1.1, b = 1$. Fraction η is set as 0.3.

the average clustering coefficient across nodes in Fig. S13(c) and results show that in this case triangles promote the spreading of the perturbations.

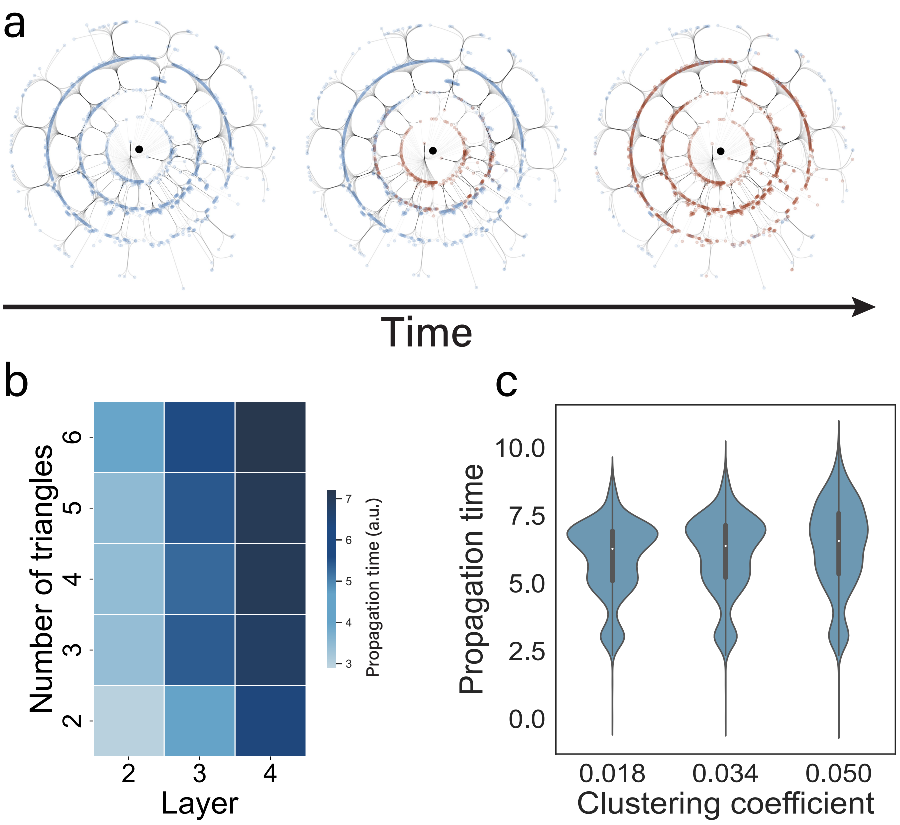

Figure S12: Impact of triangles on global propagation in PPI Networks. (a) Signal propagation snapshots of Protein-Protein Networks. Central node is the source node inducing a perturbation. Nodes in the same layer (same radial distance) have same shortest path length to the source. Brown nodes indicate the arrival of the perturbation in (an arbitrarily) fixed time. (b) Average propagation time as a function of number of triangles and layers in (a). For a given layer, triangles slow propagation. Average propagation time increases with both number of triangles and layers. (c) Signal propagation in rewired networks with varying the clustering coefficient by edge rewiring. For all three networks, triangles slow the spreading of the perturbation across layers. Panels for regulatory dynamics, $\dot{x_i}(t) = -B{x_i}^a + \alpha \sum_{j=1}^{N} A_{ij} \frac{{x_j}^b}{1+{x_j}^b}$, with $B = \alpha = 1$, $a = 0.8$, $b = 0.5$, and $N = 2035$. Fraction η is set as 0.3.

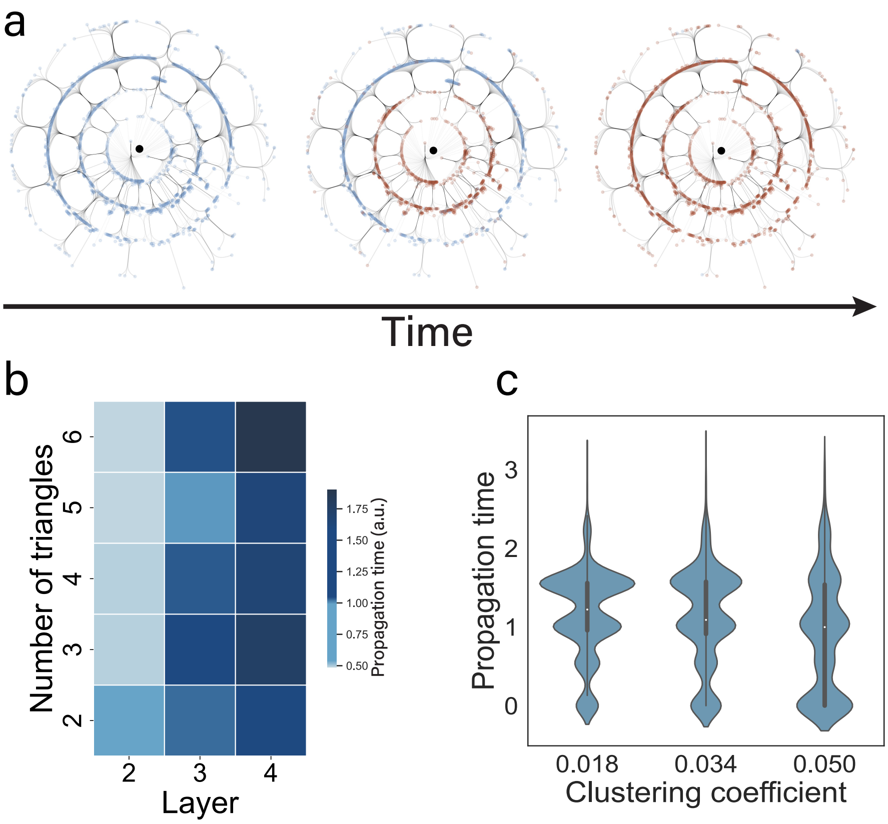

Figure S13: Impact of triangles on global propagation in PPI Networks. (a) Signal propagation snapshots of PPI Networks. Central node is the source node inducing a perturbation. Nodes in the same layer (same radial distance) have same shortest path length to the source. Brown nodes indicate the arrival of the perturbation in (an arbitrarily) fixed time. (b) Average propagation time as a function of number of triangles and layers in (a). For a given layer, triangles promote propagation. Average propagation time decreases with number of triangles but increases with layers. (c) Signal propagation in rewired networks with varying the clustering coefficient by edge rewiring. For all three networks, triangles promote the spreading of the perturbation across layers. Panels for regulatory dynamics, $\dot{x_i}(t) = -B{x_i}^a + \alpha \sum_{j=1}^{N} A_{ij} \frac{{x_j}^b}{1+{x_j}^b}$, with $B = \alpha = 1$, $a = 1.2$, $b = 2.0$, and $N = 2035$. Fraction η is set as 0.3.

4.2 Human Dynamics

Human dynamics on a macro scale captures human behaviors under different environments, like queuing or communicating on the Internet [13]. We consider the following simplified form describing the information communication between linked individuals,

$$\dot{x_i}(t) = -Bx_i^{a+b}(t) + \alpha x_i^b(t)\sum_{j=1}^{N} A_{ij}\left(y_0 - x_j^{-c}(t)\right), \tag{4.8}$$

where x_i denotes i's activity, b is an arbitrary exponent, a and c are determined by empirical data.

For this model and based on the theoretical framework, we obtain the following key terms as

$$\begin{cases} J_i = \frac{1}{aB}(x_i^*)^{1-a-b}, \\ Q_i = -\frac{\alpha c}{aB}\left(x_i^*\right)^{-a-c}, \\ \overline{Q}_L = -\frac{c\alpha}{aB}\frac{1}{d_m}\sum_{i\in K_m}\frac{1}{s_{im}}\sum_{j\in L_{im}} A_{ij}^2\left(x_i^*\right)^{-a-c}, \end{cases} \tag{4.9}$$

and derive the propagation time as

$$\tau_i = -\ln(1-\eta)\frac{\frac{1}{aB}\left(x_i^*\right)^{1-a-b}}{1-\frac{1}{\ln(1-\eta)}\frac{\eta}{1-\eta}\frac{\alpha c}{aB}\left(x_i^*\right)^{-a-c} d_i \overline{Q}_L^{(0)}}. \tag{4.10}$$

Consider that initially this system is located in the stationary stable regime

$$\begin{cases} x_i^* = \left(\frac{d_i\overline{\mathcal{H}}}{B}\right)^{\frac{1}{a+b}}, \\ \overline{\mathcal{H}} = \frac{\alpha}{N}\sum_{i=1}^{N}\frac{1}{d_i}\sum_{j=1}^{N} A_{ij}\left(y_0 - (x_j^*)^{-c}\right), \end{cases} \tag{4.11}$$

and the key terms can be rewritten accordingly in term of the degree d_i,

$$\begin{cases} J_i == \frac{1}{aB}\left(\frac{d_i\overline{\mathcal{H}}}{B}\right)^{\frac{1-b}{a}-1}, \\ Q_i = -\frac{\alpha c}{aB}\left(\frac{d_i\overline{\mathcal{H}}}{B}\right)^{-\frac{c}{a}-1}, \\ \overline{Q}_L = -\frac{c\alpha}{aB}\frac{1}{d_m}\sum_{i\in K_m}\frac{1}{s_{im}}\sum_{j\in L_{im}} A_{ij}^2\left(\frac{d_i\overline{\mathcal{H}}}{B}\right)^{-\frac{c}{a}-1}. \end{cases} \tag{4.12}$$

When $d_i \to \infty$, we calculate the two scaling coefficients $\theta_J = \frac{1-b}{a} - 1$ and $\theta_Q = -\frac{c}{a} < 0$, and the propagation time therein approaches

$$\tau_{im} \sim d_i^{\frac{1-b}{a}-1}. \tag{4.13}$$

When $b = 1$ and $f \approx 1$, the influence of triangles on the propagation time remains with $\frac{\tau_{im}}{J_j} \to -\ln(1-\eta)$. While in other conditions, triangles will not effect propagation time. Therefore, the propagation time follows

$$\tau_{im} \sim \begin{cases} d_i^{\frac{1-b}{a}-1}, b \neq 1, \\ d_i^{\frac{1-b}{a}}, b = 1. \end{cases} \tag{4.14}$$

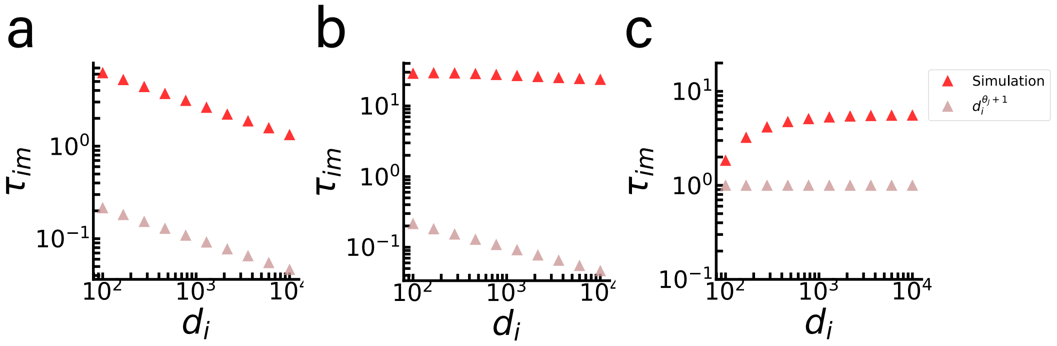

Figure S14: (a) Propagation time τ_{im} v.s. degree d_i comparing simulation with theory under the situation of independent edges with large degree. The scale between the propagation time τ_{im} and the degree d_i is depicted. Theoretical propagation time is provided by Eq. (4.13). (b-c) Propagation time τ_{im} v.s. degree d_i under the situation of triangles with large degree but in different parameter regimes. The scale between the propagation time τ_{im} and the number of triangles (also degree here) d_i is depicted. Theoretical propagation time is provided by Eq. (4.14) where $b \neq 1$ in (b). Compared to panel (b), the scaling exponent could be plus one, and theoretical propagation time is provided by Eq. (4.14) where $b = 1$ in (c). Corresponding dynamics are human dynamics, in which $B = 0.01, \alpha = 0.01, a = 1.2, c = 0.3, y_0 = 2$, $b = 0.2$ in (a) and (b), and $b = 1$ in (c). Fraction η is set as 0.3.

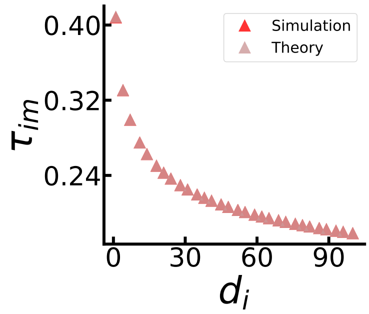

Figure S15: Propagation time τ_{im} v.s. degree d_i comparing simulation with theory under the situation of independent edges with small degree. The relation between the propagation time τ_{im} and the degree d_i is depicted, and the tendency to decrease follows the corresponding prediction in Table S5. Theoretical propagation time is provided by Eq. (4.10). Corresponding dynamics are human dynamics, in which $B = 1, \alpha = 0.1, a = 1, b = 0.2, c = 0.5, y_0 = 4$. Fraction η is set as 0.3.

4.3 Other Dynamic Models

4.3.1 Epidemic Spreading Dynamics

Epidemic spreading dynamics simulates the process of epidemic transmission among subjects. We classify subjects, for simplification, into two distinctive states, susceptible and infected, and take the probability of infected, $x_i(t)(0 < x_i(t) < 1)$. The governing dynamics of $x_i(t)$ follows [14]

$$\dot{x_i}(t) = -Bx_i(t) + \alpha\left(1 - x_i(t)\right)\sum_{j=1}^{N} A_{ij}x_j(t), \tag{4.15}$$

where B determines the speed of recovery, α determines the speed of infection, and the sum term combines the node i' susceptible probability $(\alpha(1 - x_i(t))$ and its adjacent nodes' infected probability $(x_j(t))$.

Based on the theoretical framework, we derive the key terms as

$$\begin{cases} J_i = \frac{1}{B}(1 - x_i^*), \\ Q_i = \frac{\alpha}{B}(1 - x_i^*)^2, \\ \overline{Q}_L = \frac{\alpha}{B}\sum_{i\in K_m}\frac{1}{s_{im}}\sum_{j\in L_{im}} A_{ij}^2(1 - x_i^*)^2, \end{cases} \tag{4.16}$$

and obtain the propagation time as

$$\tau_{im} = -\ln(1-\eta)\frac{\frac{1}{B}(1 - x_i^*)}{1 + \frac{1}{\ln(1-\eta)}\frac{\eta}{1-\eta}\frac{\alpha}{B}(1 - x_i^*)^2 d_i\overline{Q}_L}. \tag{4.17}$$

Based on the dynamical equation Eq. (4.15), the final state is represented as

$$\begin{cases} x_i^* = \frac{d_i\overline{\mathcal{H}}}{d_i\overline{\mathcal{H}}+B}, \\ \overline{\mathcal{H}} = \frac{\alpha}{N}\sum_{i=1}^{N}\frac{1}{d_i}\sum_{j=1}^{N} A_{ij}x_j^*. \end{cases} \tag{4.18}$$

The key terms can be rewritten as

$$\begin{cases} J_i = \frac{1}{d_i\overline{\mathcal{H}}+B}, \\ Q_i = \frac{B\alpha}{(d_i\overline{\mathcal{H}}+B)^2}, \\ \overline{Q}_L = \frac{\alpha}{B}\sum_{i\in K_m}\frac{1}{s_{im}}\sum_{j\in L_{im}} A_{ij}^2\frac{1}{(d_i\overline{\mathcal{H}}+B)^2}. \end{cases} \tag{4.19}$$

When $d_i \to \infty$, there holds $\theta_J = -1$ and $\theta_Q = -1 < 0$, and the propagation time becomes

$$\tau_{im} \sim d_i^{-1}. \tag{4.20}$$

Consider that $f \approx 1$ and target i's state x_i is comparable with its adjacency j's state x_j. The influence of triangle remains and the propagation time approaches

$$\tau_{im} \sim d_i^0. \tag{4.21}$$

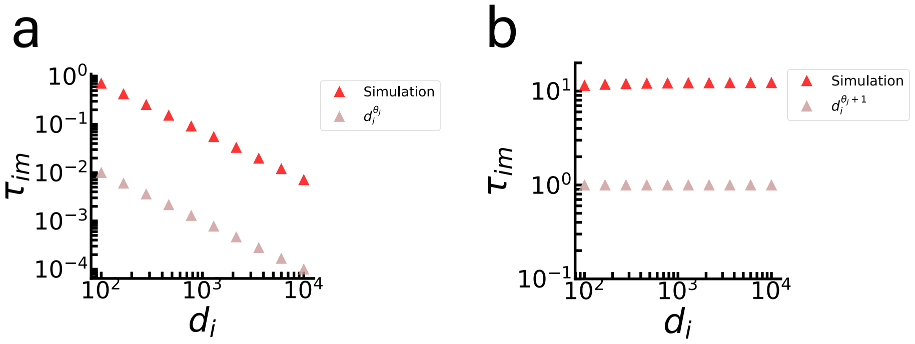

Figure S16: (a) Propagation time τ_{im} v.s. degree d_i comparing simulation with theory under the situation of independent edges with large degree. The scale between the propagation time τ_{im} and the degree d_i is depicted. Theoretical propagation time is provided by Eq. (4.20). (b) Propagation time τ_{im} v.s. degree d_i comparing simulation with theory under the situation of triangles with large degree. The scale between the propagation time τ_{im} and the number of triangles (also degree here) d_i is depicted, and the scaling exponent is plus one compared to panel (a). Theoretical propagation time is provided by Eq. (4.21). Corresponding dynamics are epidemic dynamics, in which $B = 0.01, \alpha = 0.01$. Fraction η is set as 0.3.

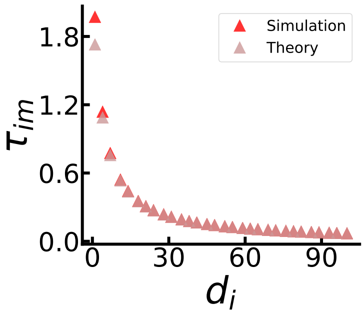

Figure S17: Propagation time τ_{im} v.s. degree d_i comparing simulation with theory under the situation of independent edges with small degree. The relation between the propagation time τ_{im} and the degree d_i is depicted, and the tendency to decrease follows the corresponding prediction in Table S5. Theoretical propagation time is provided by Eq. (4.17). Corresponding dynamics are epidemic dynamics, in which $B = 0.2, \alpha = 0.1$. Fraction η is set as 0.3.

4.3.2 Mutualistic Dynamics

In an eco−system, species often depend on each other, such as plant−pollinator networks. We take the following dynamics to describe the interdependence [15]

$$\dot{x_i}(t) = Bx_i(t)\left(1 - \frac{x_i^a(t)}{C}\right) + \alpha x_i(t)\sum_{j=1}^{N} A_{ij}\frac{x_j(t)}{1+x_j(t)}, \tag{4.22}$$

where B is the coefficient during reproducing process, and C acts the opposite effect inhibiting development of population due to limited resources.

Based on the theoretical framework, we derive the key terms as

$$\begin{cases} J_i = \frac{C}{Ba}(x_i^*)^{-a}, \\ Q_i = \frac{\alpha C}{Ba}\frac{(x_i^*)^{1-a}}{(1+x_i^*)^2}, \\ \overline{\mathcal{Q}}_L = \frac{\alpha C}{Ba}\sum_{i\in K_m}\frac{1}{s_{im}}\sum_{j\in L_{im}} A_{ij}^2 \frac{(x_i^*)^{1-a}}{(1+x_i^*)^2}, \end{cases} \tag{4.23}$$

and obtain the propagation time as

$$\tau_i = -\ln(1-\eta)\frac{\frac{C}{Ba}(x_i^*)^{-a}}{1+\frac{1}{\ln(1-\eta)}\frac{\eta}{1-\eta}\frac{\alpha C}{Ba}(x_i^*)^{1-a}d_i\overline{\mathcal{Q}}_L}. \tag{4.24}$$

Consider that initially this system is located in the stationary stable regime

$$\begin{cases} x_i^* = \left(\frac{d_i\overline{\mathcal{H}}C}{B} + C\right)^{\frac{1}{a}}, \\ \overline{\mathcal{H}} = \frac{\alpha}{N}\sum_{i=1}^{N}\frac{1}{d_i}\sum_{j=1}^{N} A_{ij}x_j^*, \end{cases} \tag{4.25}$$

and the key terms can be rewritten accordingly in term of the degree d_i, i.e.,

$$\begin{cases} J_i = \frac{1}{a(d_i\overline{\mathcal{H}}+B)}, \\ Q_i = \frac{\alpha}{a}\left(\frac{C}{B}\right)^{\frac{1}{a}}\frac{\left(d_i\overline{\mathcal{H}}+B\right)^{\frac{1}{a}-1}}{\left(1+\left(\frac{d_i\overline{\mathcal{H}}C}{B}+C\right)^{\frac{1}{a}}\right)^2}, \\ \overline{\mathcal{Q}}_L = \frac{\alpha}{a}\left(\frac{C}{B}\right)^{\frac{1}{a}}\sum_{i\in K_m}\frac{1}{s_{im}}\sum_{j\in L_{im}} A_{ij}^2(d_i\overline{\mathcal{H}}+B)^{\frac{1}{a}-1}. \end{cases} \tag{4.26}$$

When $d_i \to \infty$, we have $\theta_J = -1$ and $\theta_Q = -\frac{1}{a} < 0$, and the propagation time approaches

$$\tau_{im} \sim d_i^{-1}. \tag{4.27}$$

Consider that $f \approx 1$ and target i's state x_i is comparable with its adjacency j's state x_j. The influence of triangle remains and the propagation time approaches

$$\tau_{im} \sim d_i^0. \tag{4.28}$$

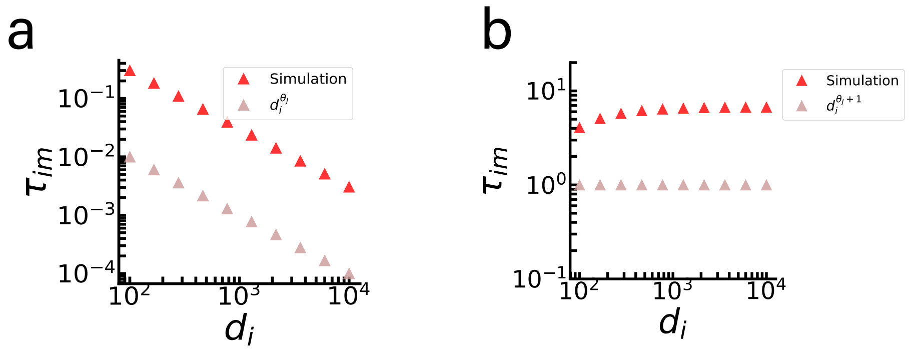

Figure S18: (a) Propagation time τ_{im} v.s. degree d_i comparing simulation with theory under the situation of independent edges with large degree. The scale between the propagation time τ_{im} and the degree d_i is depicted. Theoretical propagation time is provided by Eq. (4.27). (b) Propagation time τ_{im} v.s. degree d_i comparing simulation with theory under the situation of triangles with large degree. The scale between the propagation time τ_{im} and the number of triangles (also degree here) d_i is depicted, and the scaling exponent is plus one compared to panel (a). Theoretical propagation time is provided by Eq. (4.28). Corresponding dynamics are mutualistic dynamics, in which $B = 0.01, C = 1, \alpha = 0.01, a = 2$. Fraction η is set as 0.3.

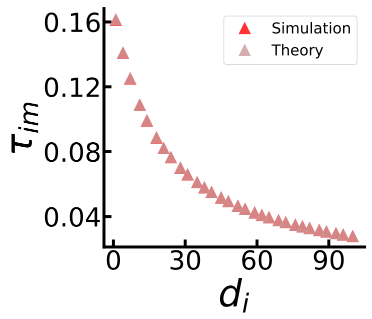

Figure S19: Propagation time τ_{im} v.s. degree d_i comparing simulation with theory under the situation of independent edges with small degree. The relation between the propagation time τ_{im} and the degree d_i is depicted, and the tendency to decrease follows the corresponding prediction in Table S5. Theoretical propagation time is provided by Eq. (4.24). Corresponding dynamics are mutualistic dynamics, in which $B = C = 1, \alpha = 0.1, a = 2$. Fraction η is set as 0.3.

4.3.3 Population Dynamics

We take the following dynamics to quantify population flow between neighbors as

$$\dot{x_i}(t) = -Bx_i^a(t) + \alpha \sum_{j=1}^{N} A_{ij} x_j^b(t), \tag{4.29}$$

where $x_i(t)$ denotes the population of site i, the interacting term depicts the flow from people j to people i as defined in [1], and b conducts the rate of population flow.

Based on the theoretical framework, we derive the key terms as

$$\begin{cases} J_i = \frac{1}{Ba}(x_i^*)^{1-a}, \\ Q_i = \frac{\alpha b}{aB}(x_i^*)^{b-a}, \\ \overline{\mathcal{Q}}_L = \frac{\alpha b}{aB}\sum_{i \in K_m} \frac{1}{s_{im}} \sum_{j \in L_{im}} A_{ij}^2 (x_i^*)^{b-a}, \end{cases} \tag{4.30}$$

and obtain the propagation time as

$$\tau_{im} = -\ln(1-\eta) \frac{\frac{1}{Ba}(x_i^*)^{1-a}}{1 + \frac{1}{\ln(1-\eta)} \frac{\eta}{1-\eta} \frac{\alpha b}{aB} (x_i^*)^{b-a} d_i \overline{\mathcal{Q}}_L}. \tag{4.31}$$

Consider that initially this system is located in the stationary stable regime

$$\begin{cases} x_i^* = \left(\frac{d_i \overline{\mathcal{H}}}{B}\right)^{\frac{1}{a}}, \\ \overline{\mathcal{H}} = \frac{\alpha}{N} \sum_{i=1}^{N} \frac{1}{d_i} \sum_{j=1}^{N} A_{ij} (x_j^*)^b, \end{cases} \tag{4.32}$$

and rewrite the key terms as

$$\begin{cases} J_i = \frac{1}{aB} \left(\frac{d_i \overline{\mathcal{H}}}{B}\right)^{\frac{1}{a}-1}, \\ Q_i = \frac{\alpha b}{aB} \left(\frac{d_i \overline{\mathcal{H}}}{B}\right)^{\frac{b}{a}-1}, \\ \overline{\mathcal{Q}}_L = \frac{\alpha b}{aB} \sum_{i \in K_m} \frac{1}{s_{im}} \sum_{j \in L_{im}} A_{ij}^2 \left(\frac{d_i \overline{\mathcal{H}}}{B}\right)^{\frac{b}{a}-1}. \end{cases} \tag{4.33}$$

When $d_i \to \infty$, we have $\theta_J = \frac{1}{a} - 1$ and $\theta_Q = \frac{b}{a} > 0$, and the propagation time approaches

$$\tau_{im} \sim d_i^{\frac{1-b}{a}-1}. \tag{4.34}$$

In this model, we have $f \ll 1$ and target i's state x_i is different from its adjacency j's state x_j. Therefore, the influence of triangle can be omitted and he propagation time approaches

$$\tau_{im} \sim d_i^{\frac{1}{a}-1}. \tag{4.35}$$

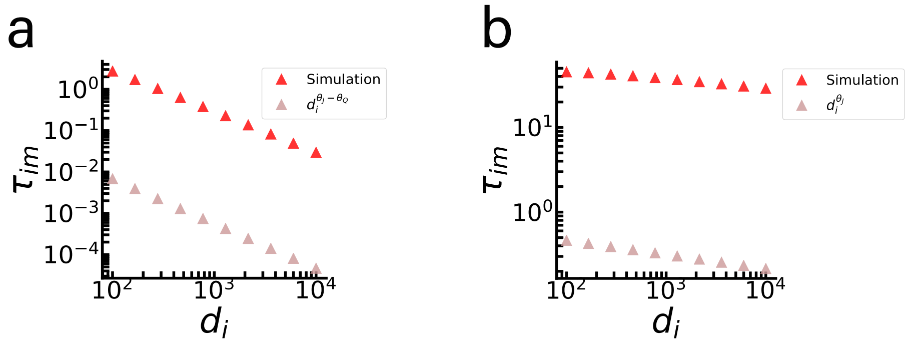

Figure S20: (a) Propagation time τ_{im} v.s. degree d_i comparing simulation with theory under the situation of independent edges with large degree. The scale between the propagation time τ_{im} and the degree d_i is depicted, and the scaling exponent is subtracted by θ_Q compared to panel (b). Theoretical propagation time is provided by Eq. (4.34). (b) Propagation time τ_{im} v.s. degree d_i comparing simulation with theory under the situation of triangles with large degree. The scale between the propagation time τ_{im} and the number of triangles (also degree here) d_i is depicted. Theoretical propagation time is provided by Eq. (4.35). Corresponding dynamics are population dynamics, in which $B = 0.01, \alpha = 0.01, a = 1.2, b = 0.5$. Fraction η is set as 0.3.

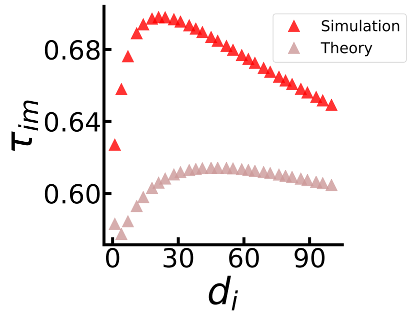

Figure S21: Propagation time τ_{im} v.s. degree d_i comparing simulation with theory under the situation of independent edges with small degree. The relation between the propagation time τ_{im} and the degree d_i is depicted, and the tendency to increase first and then decrease follows the corresponding prediction in Table S5. Theoretical propagation time is provided by Eq. (4.31). Corresponding dynamics are population dynamics, in which $B = 1, \alpha = 0.1, a = 1.1, b = 0.8$. Fraction η is set as 0.3.

4.3.4 Biochemical Dynamics

The biochemical process, characterizing the concentration of protein X_i under the consideration that hetero−dimer X_iX_j is at quazy−steady state, can be simplified as [16–18]

$$\dot{x_i}(t) = B - Cx_i(t) - \alpha x_i(t)\sum_{j=1}^{N} A_{ij}x_j(t), \tag{4.36}$$

where B determines the rate describing the influx of proteins, C determines rate in correlation with protein degradation, and A_{ij} accounts for the interaction between X_i and X_j.

Based on the theoretical framework, we derive the key terms as

$$\begin{cases} J_i = \frac{x_i^*}{B}, \\ Q_i = -\alpha(x_i^*)^2, \\ \overline{\mathcal{Q}}_L = -\alpha\sum_{i\in K_m}\frac{1}{s_{im}}\sum_{j\in L_{im}}A_{ij}^2(x_i^*)^2, \end{cases} \tag{4.37}$$

and obtain the propagation time as

$$\tau_i = -\ln(1-\eta)\frac{\frac{x_i^*}{B}}{1-\frac{1}{\ln(1-\eta)}\frac{\eta}{1-\eta}\alpha(x_i^*)^2 d_i\overline{\mathcal{Q}}_L}. \tag{4.38}$$

Consider that initially this system is located in the stationary stable regime

$$\begin{cases} x_i^* = \frac{B}{d_i\overline{\mathcal{H}}+C}, \\ \overline{\mathcal{H}} = \frac{\alpha}{N}\sum_{i=1}^{N}\frac{1}{d_i}\sum_{j=1}^{N}A_{ij}x_j^*. \end{cases} \tag{4.39}$$

The key parameters can be rewritten with respect to the degree d_i, i.e.,

$$\begin{cases} J_i = \frac{1}{d_i\overline{\mathcal{H}}+C}, \\ Q_i = -\frac{B\alpha}{(d_i\overline{\mathcal{H}}+C)^2}, \\ \overline{\mathcal{Q}}_L = -B\alpha\sum_{i\in K_m}\frac{1}{s_{im}}\sum_{j\in L_{im}}A_{ij}^2\frac{1}{(d_i\overline{\mathcal{H}}+C)^2}. \end{cases} \tag{4.40}$$

When $d_i \to \infty$, we have the scaling coefficients $\theta_J = -1$ and $\theta_Q = -1 < 0$, and obtain that the propagation time approaches

$$\tau_{im} \sim d_i^{-1} \tag{4.41}$$

Consider that $f \approx 1$ and target i's state x_i is comparable with its adjacency j's state x_j. The influence of triangle remains and the propagation time approaches

$$\tau_{im} \sim d_i^0 \tag{4.42}$$

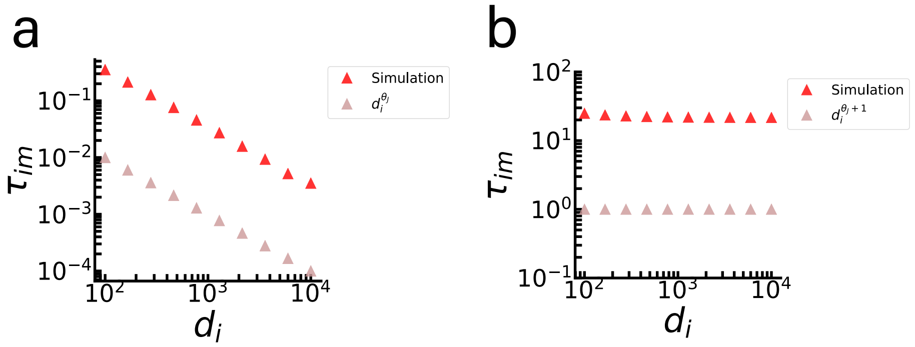

Figure S22: (a) Propagation time τ_{im} v.s. degree d_i comparing simulation with theory under the situation of independent edges with large degree. The scale between the propagation time τ_{im} and the degree d_i is depicted. Theoretical propagation time is provided by Eq. (4.41). (b) Propagation time τ_{im} v.s. degree d_i comparing simulation with theory under the situation of triangles with large degree. The scale between the propagation time τ_{im} and the number of triangles (also degree here) d_i is depicted, and the scaling exponent is plus one compared to panel (a). Theoretical propagation time is provided by Eq. (4.42). Corresponding dynamics are biochemical dynamics, in which $B = C = 0.01, \alpha = 0.01$. Fraction η is set as 0.3.

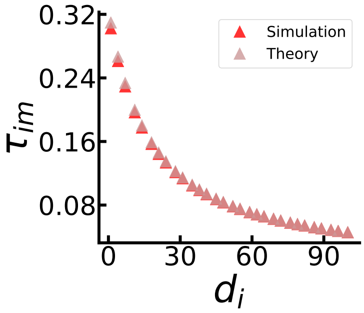

Figure S23: Propagation time τ_{im} v.s. degree d_i comparing simulation with theory under the situation of independent edges with small degree. The relation between the propagation time τ_{im} and the degree d_i is depicted, and the tendency to decrease follows the corresponding prediction in Table S5. Theoretical propagation time is provided by Eq. (4.38). Corresponding dynamics are biochemical dynamics, in which $B = C = 1, \alpha = 0.1$. Fraction η is set as 0.3.

4.3.5 Inhibitory Dynamics

Similar to mutualistic dynamics, where species depend on other species, we consider the following dynamics with linear interdependence [19]

$$\dot{x_i}(t) = -Bx_i(t)\left(1 - \frac{x_i(t)}{C}\right)^2 + \alpha x_i(t)\sum_{j=1}^{N} A_{ij}x_j(t), \tag{4.43}$$

where B is the coefficient during reproducing process, C acts the opposite effect inhibiting development of population due to limited resources, and the interdependence between species j and i following the linear function $\alpha x_j(t)$. Based on the theoretical framework, we derive the key terms as

$$\begin{cases} J_i = \frac{C}{2B}\frac{1}{x_i^*\left(\frac{x_i^*}{C}-1\right)}, \\ Q_i = \frac{\alpha C}{2B}\frac{1}{\frac{x_i^*}{C}-1}, \\ \overline{\mathcal{Q}}_L = \frac{\alpha C}{2B}\sum_{i\in K_m}\frac{1}{s_{im}}\sum_{j\in L_{im}} A_{ij}^2 \frac{1}{\frac{x_i^*}{C}-1}, \end{cases} \tag{4.44}$$

and obtain the propagation time as

$$\tau_i = -\ln(1-\eta)\frac{\frac{C}{2B}\frac{1}{x_i^*\left(\frac{x_i^*}{C}-1\right)}}{1 - \frac{1}{\ln(1-\eta)}\frac{\eta}{1-\eta}\frac{\alpha C}{2B}\frac{1}{\frac{x_i^*}{C}-1}d_i\overline{\mathcal{Q}}_L}. \tag{4.45}$$

Consider that initially this system is located in the stationary stable regime

$$\begin{cases} x_i^* = C\left(\left(\frac{d_i\overline{\mathcal{H}}}{B}\right)^{\frac{1}{2}} + 1\right), \\ \overline{\mathcal{H}} = \frac{\alpha}{N}\sum_{i=1}^{N}\frac{1}{d_i}\sum_{j=1}^{N} A_{ij}x_j^*, \end{cases} \tag{4.46}$$

and the key terms can be rewritten accordingly in term of the degree d_i, i.e.,

$$\begin{cases} J_i = \frac{1}{2B^{\frac{1}{2}}}\frac{1}{\left(\left(\frac{d_i\overline{\mathcal{H}}}{B}\right)^{\frac{1}{2}}+1\right)\left(d_i\overline{\mathcal{H}}\right)^{\frac{1}{2}}}, \\ Q_i = \frac{\alpha C}{2B^{\frac{1}{2}}}\frac{1}{\left(d_i\overline{\mathcal{H}}\right)^{\frac{1}{2}}}, \\ \overline{\mathcal{Q}}_L = \frac{\alpha C}{2B^{\frac{1}{2}}}\sum_{i\in K_m}\frac{1}{s_{im}}\sum_{j\in L_{im}} A_{ij}^2\frac{1}{\left(d_i\overline{\mathcal{H}}\right)^{\frac{1}{2}}}. \end{cases} \tag{4.47}$$

When $d_i \to \infty$, we have $\theta_J = -1$ and $\theta_Q = \frac{1}{2} > 0$, and obtain that the propagation time approaches

$$\tau_{im} \sim d_i^{-\frac{3}{2}}. \tag{4.48}$$

In this model, we have $f \ll 1$ and target i's state x_i is different from its adjacency j's state x_j. Therefore, the influence of triangle can be omitted, and the propagation time approaches

$$\tau_{im} \sim d_i^{-1} \tag{4.49}$$

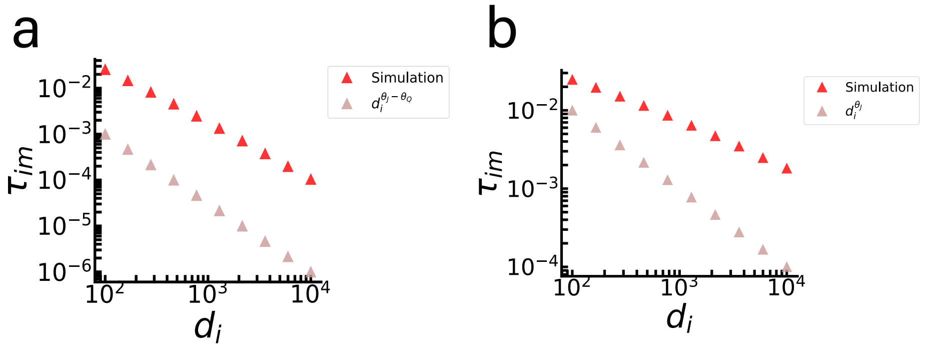

Figure S24: (a) Propagation time τ_{im} v.s. degree d_i comparing simulation with theory under the situation of independent edges with large degree. The scale between the propagation time τ_{im} and the degree d_i is depicted, and the scaling exponent is subtracted by θ_Q compared to panel (b). Theoretical propagation time is provided by Eq. (4.48). (b) Propagation time τ_{im} v.s. degree d_i comparing simulation with theory under the situation of triangles with large degree. The scale between the propagation time τ_{im} and the degree d_i is depicted. Theoretical propagation time is provided by Eq. (4.49). Corresponding dynamics are inhibitory dynamics, in which $B = 0.01, \alpha = 0.01, C = 1$. Fraction η is set as 0.3.

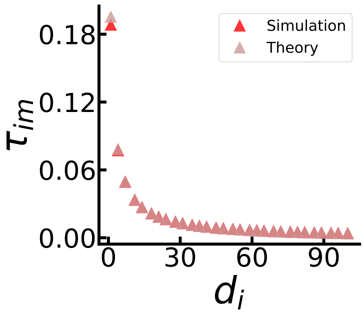

Figure S25: Propagation time τ_{im} v.s. degree d_i comparing simulation with theory under the situation of independent edges with small degree. The relation between the propagation time τ_{im} and the degree d_i is depicted, and the tendency to decrease follows the corresponding prediction in Table S5. Theoretical propagation time is provided by Eq. (4.45), almost covering on the simulated curve (the red trianglular curve). Corresponding dynamics are inhibitory dynamics, in which $B = 0.2, \alpha = 0.1, C = 1$. Fraction η is set as 0.3.

4.4 Brief Summary of Dynamic Models

Dynamic Models	Dynamic Equation	J_i	θ_J	$Q_{im}^{(0)}$	θ_Q
Regulatory ($\mathbb{R}$)	$\dot{x}_i(t) = -Bx_i^a(t) + \alpha \sum_{j=1}^{N} A_{ij} \frac{x_j^b(t)}{1+x_j^b(t)}$	$\frac{1}{aB}\left(\frac{d_i\overline{\mathcal{H}}}{B}\right)^{\frac{1}{a}-1}$	$\frac{1}{a}-1$	$\frac{\alpha b}{aB}\frac{\left(\frac{d_i\overline{\mathcal{H}}}{B}\right)^{\frac{b-a}{a}}}{\left(\left(\frac{d_i\overline{\mathcal{H}}}{B}\right)^{\frac{b}{a}}+1\right)^2}$	$-\frac{b}{a}$
Human ($\mathbb{H}$)	$\dot{x}_i(t) = -Bx_i^{a+b}(t) + \alpha x_i^b(t) \sum_{j=1}^{N} A_{ij}\left(y_0 - x_j^{-c}(t)\right)$	$\frac{1}{aB}\left(\frac{d_i\overline{\mathcal{H}}}{B}\right)^{\frac{1-b}{a}-1}$	$\frac{1-b}{a}-1$	$\frac{\alpha c}{aB}\left(\frac{d_i\overline{\mathcal{H}}}{B}\right)^{-\frac{c}{a}-1}$	$-\frac{c}{a}$
Epidemics ($\mathbb{E}$)	$\dot{x}_i(t) = -Bx_i(t) + \alpha\left(1 - x_i(t)\right) \sum_{j=1}^{N} A_{ij} x_j(t)$	$\frac{1}{d_i\overline{\mathcal{H}}+B}$	-1	$\frac{\alpha B}{\left(d_i\overline{\mathcal{H}}+B\right)^2}$	-1
Mutualistic ($\mathbb{M}$)	$\dot{x}_i(t) = Bx_i(t)\left(1 - \frac{x_i^a(t)}{C}\right) + \alpha x_i(t) \sum_{j=1}^{N} A_{ij} x_j(t)$	$\frac{1}{a(d_i\overline{\mathcal{H}}+B)}$	-1	$\frac{\alpha}{a}\frac{(\frac{C}{B})^{\frac{1}{a}}\left(d_i\overline{\mathcal{H}}+B\right)^{\frac{1}{a}-1}}{\left(1+\left(\frac{d_i\overline{\mathcal{H}}C}{B}+C\right)^{\frac{1}{a}}\right)^2}$	$-\frac{1}{a}$
Population ($\mathbb{P}$)	$\dot{x}_i(t) = -Bx_i^a(t) + \alpha \sum_{j=1}^{N} A_{ij} x_j^b(t)$	$\frac{1}{aB}\left(\frac{d_i\overline{\mathcal{H}}}{B}\right)^{\frac{1}{a}-1}$	$\frac{1}{a}-1$	$\frac{\alpha b}{aB}\left(\frac{d_i\overline{\mathcal{H}}}{B}\right)^{\frac{b}{a}-1}$	$\frac{b}{a}$
Biochemical ($\mathbb{B}$)	$\dot{x}_i(t) = B - Cx_i(t) - \alpha x_i(t) \sum_{j=1}^{N} A_{ij} x_j(t)$	$\frac{1}{d_i\overline{\mathcal{H}}+C}$	-1	$-\frac{B}{\left(d_i\overline{\mathcal{H}}+C\right)^2}$	-1
Inhibitory ($\mathbb{I}$)	$\dot{x}_i(t) = -Bx_i(t)\left(1 - \frac{x_i(t)}{C}\right)^2 + \alpha x_i(t) \sum_{j=1}^{N} A_{ij} x_j(t)$	$\frac{1}{2B^{\frac{1}{2}}}\frac{\left(d_i\overline{\mathcal{H}}\right)^{-\frac{1}{2}}}{\left(\left(\frac{d_i\overline{\mathcal{H}}}{B}\right)^{\frac{1}{2}}+1\right)}$	-1	$\frac{\alpha C}{2B^{\frac{1}{2}}}\frac{1}{\left(d_i\overline{\mathcal{H}}\right)^{\frac{1}{2}}}$	$\frac{1}{2}$

Table S6: Scaling coefficients and key quantities under different dynamic models in the effect of independent edges in the local case.

Dynamic Models	Key Parameters	$\theta_J > 0$	$\theta_J = 0$	$\theta_J < 0$	
Regulatory	$\theta_Q < 0$ $B = 1$	$a = 0.8, b = 0.5$ 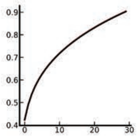	$a = 1.0, b = 0.5$ 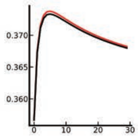	$a = 1.2, b = 0.5$ 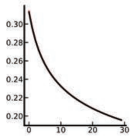	$a = 1.2, b = 2.0$ 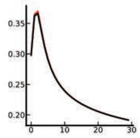
Population	$\theta_Q > 0$ $B = 1$	$a = 0.8, b = 0.2$ 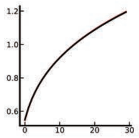	$a = 1.0, b = 0.2$ 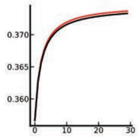	$a = 1.2, b = 0.2$ 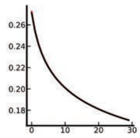	$a = 1.2, b = 0.6$ 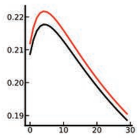
Epidemics	$\theta_Q < 0$ $B = 1$			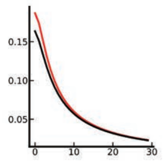	
Mutualistic	$\theta_Q < 0$ $B = 1$ $C = 1$			$a = 2.0$ 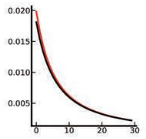	
Human	$\theta_Q < 0$ $B = 1$ $b = 0.2$	$a = 0.6, c = 0.2$ 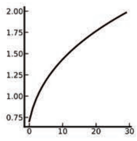	$a = 0.8, c = 0.2$ 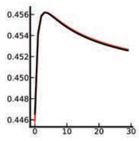	$a = 1.0, c = 0.2$ 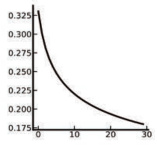	
Biochemical	$\theta_Q < 0$ $B = 1$ $C = 1$			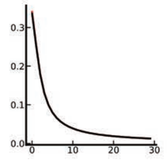	

Table S7: Simulations (in black) and theory (in red) to show the relation between propagation time and degree under different dynamic models in different parameter spaces. Theory is based on the analytical formula Eq. (2.51). Degree ranges from 1 to 30.

Dynamic Models	Key Parameters	$\theta_J > 0$	$\theta_J = 0$	$\theta_J < 0$	
Regulatory	$\theta_Q < 0$<br>$B = 1$	$a = 0.8, b = 0.5$<br>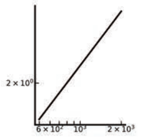	$a = 1.0, b = 0.5$<br>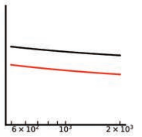	$a = 1.2, b = 0.5$<br>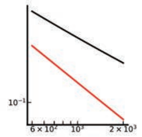	$a = 1.2, b = 2.0$<br>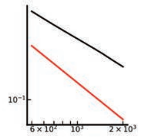
Population	$\theta_Q > 0$<br>$B = 1$	$a = 0.8, b = 0.2$<br>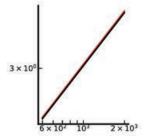	$a = 1.0, b = 0.2$<br>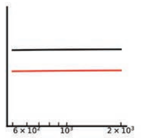	$a = 1.2, b = 0.2$<br>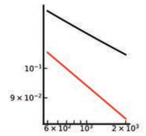	$a = 1.2, b = 0.6$<br>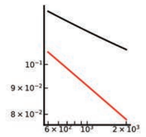
Epidemics	$\theta_Q < 0$<br>$B = 1$			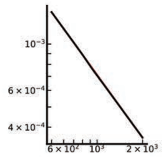	
Mutualistic	$\theta_Q < 0$<br>$B = 1$<br>$C = 1$			$a = 2.0$<br>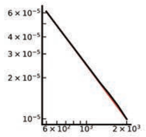	
Human	$\theta_Q < 0$<br>$B = 1$<br>$b = 0.2$	$a = 0.6, c = 0.2$<br>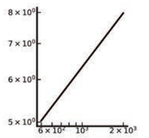	$a = 0.8, c = 0.2$<br>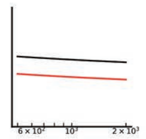	$a = 1.0, c = 0.2$<br>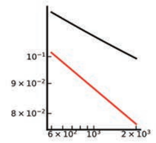	
Biochemical	$\theta_Q < 0$<br>$B = 1$<br>$C = 1$			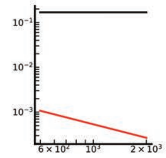	

Table S8: Simulations (in black) and theory (in red) to show the relation between propagation time and degree under different dynamic models in different parameter spaces. Theory is based on the analytical formula Eq. (2.51). Degree ranges from 500 to 2000, i.e., considering the relation on the scale of degree from 10^2 to 10^3.

Dynamic Models	Key Parameters	$\theta_J > 0$	$\theta_J = 0$	$\theta_J < 0$	
Regulatory	$\theta_Q < 0$ $B = 1$	$a = 0.8, b = 0.5$ 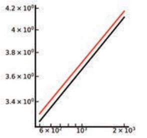	$a = 1.0, b = 0.5$ 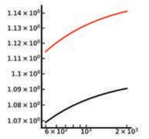	$a = 1.2, b = 0.5$ 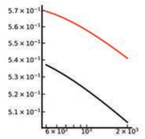	$a = 1.2, b = 2.0$ 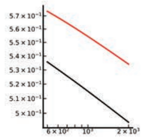
Population	$\theta_Q > 0$ $B = 1$	$a = 0.8, b = 0.2$ 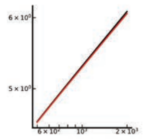	$a = 1.0, b = 0.2$ 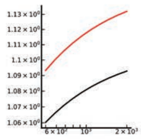	$a = 1.2, b = 0.2$ 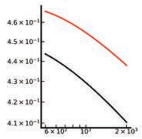	$a = 1.2, b = 0.6$ 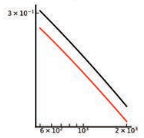
Epidemics	$\theta_Q < 0$ $B = 1$			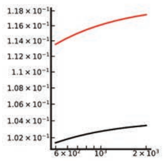	
Mutualistic	$\theta_Q < 0$ $B = 1$ $C = 1$			$a = 2.0$ 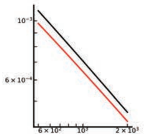	
Human	$\theta_Q < 0$ $B = 1$ $b = 0.2$	$a = 0.6, c = 0.2$ 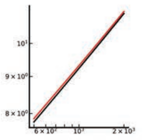	$a = 0.8, c = 0.2$ 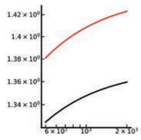	$a = 1.0, c = 0.2$ 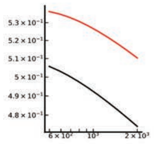	
Biochemical	$\theta_Q < 0$ $B = 1$ $C = 1$			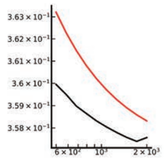	

Table S9: Simulations (in black) and theory (in red) to show the relation between propagation time and number of triangles under different dynamic models. Theory is based on the analytical formula Eq. (2.57). Degree ranges from 500 to 2000.

5 Methods

5.1 Applicability Analysis for Gauss Iteration Method

For the global case, we employ Gauss iteration method and the iterative formula is derived by Taylor expansion shown as

$$\begin{aligned}\Delta x_{i_k}(t) - \eta\Delta x_{i_k}(\infty) = {} & \Delta x_{i_k}(T(m \to i_{k-1})) - \eta\Delta x_{i_k}(\infty) \\ & + \frac{d\left[\Delta x_{i_k}(t) - \eta\Delta x_{i_k}(\infty)\right]}{dt}\Big|_{t=T(m\to i_{k-1})}\big(t - T(m \to i_{k-1})\big) \\ & + \frac{d^2\left[\Delta x_{i_k}(t) - \eta\Delta x_{i_k}(\infty)\right]}{dt^2}\Big|_{t=T(m\to i_{k-1})}\frac{\big(t - T(m \to i_{k-1})\big)^2}{2!} + \cdots .\end{aligned} \tag{5.1}$$

Set $t = T(m \to i_k)$ and the left side of the equation is 0. Through Taylor expansion, the equation holds

$$\begin{aligned}T(m \to i_k) = {} & T(m \to i_{k-1}) - \left(\left(\Delta x_{i_k}(t) - \eta\Delta x_{i_k}(\infty)\right)\left(\frac{d\left[\Delta x_{i_k}(t) - \eta\Delta x_{i_k}(\infty)\right]}{dt}\right)^{-1}\right)\Big|_{t=T(m\to i_{k-1})} \\ & - \frac{\big(T(m \to i_k) - T(m \to i_{k-1})\big)^2}{2!}\left(\frac{d^2\left[\Delta x_{i_k}(t) - \eta\Delta x_{i_k}(\infty)\right]}{dt^2}\left(\frac{d\left[\Delta x_{i_k}(t) - \eta\Delta x_{i_k}(\infty)\right]}{dt}\right)^{-1}\right)\Big|_{t=T(m\to i_{k-1})} \\ & - \frac{\big(T(m \to i_k) - T(m \to i_{k-1})\big)^3}{3!}\left(\frac{d^3\left[\Delta x_{i_k}(t) - \eta\Delta x_{i_k}(\infty)\right]}{dt^3}\left(\frac{d\left[\Delta x_{i_k}(t) - \eta\Delta x_{i_k}(\infty)\right]}{dt}\right)^{-1}\right)\Big|_{t=T(m\to i_{k-1})} \\ & - \cdots .\end{aligned} \tag{5.2}$$

If we omit terms of second order and above, the equation holds

$$T(m \to i_k) = T(m \to i_{k-1}) - \left(\left(\Delta x_{i_k}(t) - \eta\Delta x_{i_k}(\infty)\right)\left(\frac{d\left[\Delta x_{i_k}(t) - \eta\Delta x_{i_k}(\infty)\right]}{dt}\right)^{-1}\right)\Big|_{t=T(m\to i_{k-1})}. \tag{5.3}$$

The latter terms of Eq. (5.3) derived by Newton method and the exact solution Eq.(5.2) are different, and these terms are hard to present. Newton iterative method makes the calculation process easier and the latter terms become

$$\begin{aligned}& \frac{1}{2!}\left(\left(\Delta x_{i_k}(t) - \eta\Delta x_{i_k}(\infty)\right)\frac{d^2\left[\Delta x_{i_k}(t) - \eta\Delta x_{i_k}(\infty)\right]}{dt^2}\left(\frac{d\left[\Delta x_{i_k}(t) - \eta\Delta x_{i_k}(\infty)\right]}{dt}\right)^{-2}\right)\Big|_{t=T(m\to i_{k-1})} \\ & + \frac{1}{3!}\left(\left(\Delta x_{i_k}(t) - \eta\Delta x_{i_k}(\infty)\right)^2\frac{d^3\left[\Delta x_{i_k}(t) - \eta\Delta x_{i_k}(\infty)\right]}{dt^3}\left(\frac{d\left[\Delta x_{i_k}(t) - \eta\Delta x_{i_k}(\infty)\right]}{dt}\right)^{-3}\right)\Big|_{t=T(m\to i_{k-1})}.\end{aligned} \tag{5.4}$$

We consider the case in the k-th layer. The corresponding value is expressed as

$$\begin{aligned}\frac{d\left[\Delta x_{i_k}(t) - \eta\Delta x_{i_k}(\infty)\right]}{dt} = {} & H_1\big(x_{i_k}^*\big)\left[\frac{F\big(x_{i_k}^*\big)}{H_1\big(x_{i_k}^*\big)}\right]'\left(\Delta x_{i_k}(t) - \eta\Delta x_{i_k}(\infty)\right) \\ & + H_1\big(x_{i_k}^*\big)\sum_{\substack{j=1 \\ j\neq i_{k-1}}}^{N} A_{i_k j}H_2'\big(x_j^*\big)\left(\Delta x_j(t) - \eta\Delta x_j(\infty)\right) \\ & + H_1\big(x_{i_k}^*\big)A_{i_k i_{k-1}}H_2'\big(x_{i_{k-1}}^*\big)\left(\Delta x_{i_{k-1}}(t) - \eta\Delta x_{i_{k-1}}(\infty)\right).\end{aligned} \tag{5.5}$$

We derive the second-order derivative equation

$$\begin{aligned}\frac{d^2\left[\Delta x_{i_k}(t)-\eta\Delta x_{i_k}(\infty)\right]}{dt^2} &= H_1\big(x^*_{i_k}\big)\left[\frac{F\big(x^*_{i_k}\big)}{H_1\big(x^*_{i_k}\big)}\right]^{'}\frac{d\big[\Delta x_{i_k}(t)-\eta\Delta x_{i_k}(\infty)\big]}{dt}\\ &\quad + H_1\big(x^*_{i_k}\big)\sum_{j=1}^{N}A_{i_kj}H_2^{'}\big(x^*_j\big)\frac{d\big[\Delta x_j(t)-\eta\Delta x_j(\infty)\big]}{dt}.\end{aligned} \tag{5.6}$$

The first specific value is

$$\begin{aligned}&\left(\big(\Delta x_{i_k}(t)-\eta\Delta x_{i_k}(\infty)\big)\left(\frac{d\left[\Delta x_{i_k}(t)-\eta\Delta x_{i_k}(\infty)\right]}{dt}\right)^{-1}\right)\Big|_{t=T(m\to i_{k-1})}\\ =&\frac{1-H_1\big(x^*_{i_k}\big)\sum_{\substack{j=1\\ j\neq i_{k-1}}}^{N}A_{i_kj}H_2^{'}\big(x^*_j\big)\frac{\Delta x_j(t)-\eta\Delta x_j(\infty)}{\frac{d\left[\Delta x_{i_k}(t)-\eta\Delta x_{i_k}(\infty)\right]}{dt}}\Big|_{t=T(m\to i_{k-1})}}{H_1\big(x^*_{i_k}\big)\left[\frac{F\left(x^*_{i_k}\right)}{H_1\left(x^*_{i_k}\right)}\right]^{'}},\\ =&\frac{1-U_{i_k}}{H_1\big(x^*_{i_k}\big)\left[\frac{F\left(x^*_{i_k}\right)}{H_1\left(x^*_{i_k}\right)}\right]^{'}},\end{aligned} \tag{5.7}$$

in which

$$\begin{aligned}U_{i_k} &= H_1\big(x^*_{i_k}\big)\sum_{\substack{j=1\\ j\neq i_{k-1}}}^{N}A_{i_kj}H_2^{'}\big(x^*_j\big)\frac{\Delta x_j(t)-\eta\Delta x_j(\infty)}{\Delta x_{i_k}(t)-\eta\Delta x_{i_k}(\infty)}\frac{\Delta x_{i_k}(t)-\eta\Delta x_{i_k}(\infty)}{\frac{d\left[\Delta x_{i_k}(t)-\eta\Delta x_{i_k}(\infty)\right]}{dt}}\Big|_{t=T(m\to i_{k-1})}\\ &\approx -H_1\big(x^*_{i_k}\big)\sum_{\substack{j=1\\ j\neq i_{k-1}}}^{N}A_{i_kj}H_2^{'}\big(x^*_j\big)\frac{\Delta x_j(\infty)}{\Delta x_{i_k}(\infty)}\frac{\delta_j(T(m\to i_{k-1}))-\eta}{\delta_{i_k}(T(m\to i_{k-1}))-\eta}\frac{1}{H_1\big(x^*_{i_k}\big)\left[\frac{F\left(x^*_{i_k}\right)}{H_1\left(x^*_{i_k}\right)}\right]^{'}}.\end{aligned} \tag{5.8}$$

$\delta_j(T(m\to i_{k-1}))$ approximates to $\delta_{i_k}(T(m\to i_{k-1}))$ as node j is i_k's neighbor and their values are comparatively small with $\eta-$fraction, hence the expression U_{i_k} holds

$$U_{i_k}\approx -\frac{H_1\big(x^*_{i_k}\big)\sum_{\substack{j=1\\ j\neq i_{k-1}}}^{N}A_{i_kj}H_2^{'}\big(x^*_j\big)\frac{\Delta x_j(\infty)}{\Delta x_{i_k}(\infty)}}{H_1\big(x^*_{i_k}\big)\left[\frac{F\left(x^*_{i_k}\right)}{H_1\left(x^*_{i_k}\right)}\right]^{'}} \tag{5.9}$$

if differentiating both sides of Eq. (2.3), this value is a bounded value. The second term is

$$\begin{aligned}&\left(\left(\frac{d^2\left[\Delta x_{i_k}(t)-\eta\Delta x_{i_k}(\infty)\right]}{dt^2}\right)\left(\frac{d\big[\Delta x_{i_k}(t)-\eta\Delta x_{i_k}(\infty)\big]}{dt}\right)^{-1}\right)\Big|_{t=T(m\to i_{k-1})}\\ &= H_1\big(x^*_{i_k}\big)\left[\frac{F\big(x^*_{i_k}\big)}{H_1\big(x^*_{i_k}\big)}\right]^{'}+H_1\big(x^*_{i_k}\big)\sum_{j=1}^{N}A_{i_kj}H_2^{'}\big(x^*_j\big)\frac{\frac{d\left[\Delta x_j(t)-\eta\Delta x_j(\infty)\right]}{dt}}{\frac{d\left[\Delta x_{i_k}(t)-\eta\Delta x_{i_k}(\infty)\right]}{dt}}\Big|_{t=T(m\to i_{k-1})}\\ &= H_1\big(x^*_{i_k}\big)\left[\frac{F\big(x^*_{i_k}\big)}{H_1\big(x^*_{i_k}\big)}\right]^{'}\big(1-V_{i_k}\big),\end{aligned} \tag{5.10}$$

in which

$$
\begin{aligned}
V_{i_k} &= -\frac{1}{H_1\left(x_{i_k}^*\right)\left[\frac{F\left(x_{i_k}^*\right)}{H_1\left(x_{i_k}^*\right)}\right]'} H_1\left(x_{i_k}^*\right) \sum_{j=1}^{N} A_{i_k j} H_2'\left(x_j^*\right) \frac{\frac{d\left[\Delta x_j(t)-\eta \Delta x_j(\infty)\right]}{dt}}{\frac{d\left[\Delta x_{i_k}(t)-\eta \Delta x_{i_k}(\infty)\right]}{dt}} \Big|_{t=T(m\to i_{k-1})}, \\
&= -\frac{1}{H_1\left(x_{i_k}^*\right)\left[\frac{F\left(x_{i_k}^*\right)}{H_1\left(x_{i_k}^*\right)}\right]'} H_1\left(x_{i_k}^*\right) \sum_{j=1}^{N} A_{i_k j} H_2'\left(x_j^*\right) \frac{\frac{d\left[\Delta x_j(t)-\eta \Delta x_j(\infty)\right]}{dt}}{\Delta x_j(t)-\eta \Delta x_j(\infty)} \\
&\quad \frac{\Delta x_{i_k}(t)-\eta \Delta x_{i_k}(\infty)}{\frac{d\left[\Delta x_{i_k}(t)-\eta \Delta x_{i_k}(\infty)\right]}{dt}} \frac{\Delta x_j(t)-\eta \Delta x_j(\infty)}{\Delta x_{i_k}(t)-\eta \Delta x_{i_k}(\infty)} \Big|_{t=T(m\to i_{k-1})} \\
&\approx -\frac{1}{H_1\left(x_{i_k}^*\right)\left[\frac{F\left(x_{i_k}^*\right)}{H_1\left(x_{i_k}^*\right)}\right]'} H_1\left(x_{i_k}^*\right) \sum_{j=1}^{N} A_{i_k j} H_2'\left(x_j^*\right) \frac{\Delta x_j(\infty)}{\Delta x_{i_k}(\infty)} \frac{H_1\left(x_j^*\right)\left[\frac{F\left(x_j^*\right)}{H_1\left(x_j^*\right)}\right]'}{H_1\left(x_{i_k}^*\right)\left[\frac{F\left(x_{i_k}^*\right)}{H_1\left(x_{i_k}^*\right)}\right]'} \frac{\delta_j(T(m\to i_{k-1}))-\eta}{\delta_{i_k}(T(m\to i_{k-1}))-\eta} \\
&\approx J_{i_k} H_1\left(x_{i_k}^*\right) \sum_{j=1}^{N} A_{i_k j} H_2'\left(x_j^*\right) \frac{\Delta x_j(\infty)}{\Delta x_{i_k}(\infty)} \frac{J_{i_k}}{J_j}
\end{aligned} \tag{5.11}
$$

for $\delta_j(T(m \to i_{k-1}))$ approximating to $\delta_{i_k}(T(m \to i_{k-1}))$. If the difference between J_{ik} and J_j is not so large, then using Eq. (3.3) there is $V_{i_k} \to 1$.

In summary,

$$
\begin{aligned}
&\frac{1}{2!}\left(\left(\Delta x_{i_k}(t)-\eta \Delta x_{i_k}(\infty)\right) \frac{d^2\left[\Delta x_{i_k}(t)-\eta \Delta x_{i_k}(\infty)\right]}{dt^2}\left(\frac{d\left[\Delta x_{i_k}(t)-\eta \Delta x_{i_k}(\infty)\right]}{dt}\right)^{-2}\right)\Big|_{t=T(m\to i_{k-1})} \\
=&\frac{1}{2!}\left(1-U_{i_k}\right)\left(1-V_{i_k}\right) \to 0.
\end{aligned} \tag{5.12}
$$

5.2 Quantification of Q_{im} by Iteration Method

We represent the explicit expression of propagation time τ_{im} but some parameters are hardly exhibited. Here we employ an iterative method. After perturbing node m's states and then quantifying the consequence of instant and distant node i's state, we solve the following parameters as

$$\begin{cases} \mathcal{E}_{\Delta_{im}} = \sum_{j\in\Delta_{im}} \frac{A_{ij}A_{jm}}{A_{im}} Q_{jm}, \\ \mathcal{E}_{L_{im}} = J_i H_1(x_i^*) H_2^{'}(x_i^*) \sum_{j\in L_{im}} A_{ij}^2 Q_{ji}, \end{cases} \tag{5.13}$$

in which

$$\begin{cases} Q_{jm} = \frac{J_j H_1(x_j^*) H_2^{'}(x_j^*)}{1-\mathcal{E}_{jm}}, \\ \mathcal{E}_{jm} = \frac{-1+J_j H_1(x_j^*) H_2^{'}(x_j^*) \sum_{k\in L_{jm}} A_{jk}^2 Q_{kj}}{1+\sum_{k\in\Delta_{jm}} \frac{A_{jk}A_{km}}{A_{jm}} Q_{km}} + 1. \end{cases} \tag{5.14}$$

If we take node i as perturbed, we calculate the variation of its adjacent nodes' states j and obtain that

$$\begin{cases} Q_{ji} = \frac{J_j H_1(x_j^*) H_2^{'}(x_j^*)}{1-\mathcal{E}_{ji}}, \\ \mathcal{E}_{ji} = \frac{-1+J_j H_1(x_j^*) H_2^{'}(x_j^*) \sum_{k\in L_{ji}} A_{jk}^2 Q_{kj}}{1+\sum_{k\in\Delta_{ji}} \frac{A_{jk}A_{ki}}{A_{ji}} Q_{ki}} + 1. \end{cases} \tag{5.15}$$

Here we find that Q_{ji} is similar with Q_{jm}, and calculate their difference

$$\begin{aligned} \Big|Q_{jm} - Q_{ji}\Big| &= \Big|J_j H_1(x_j^*) H_2^{'}(x_j^*)\Big| \Bigg| \frac{\mathcal{E}_{jm} - \mathcal{E}_{ji}}{\big(1-\mathcal{E}_{ji}\big)\big(1-\mathcal{E}_{jm}\big)} \Bigg| \\ &= \Big|J_j H_1(x_j^*) H_2^{'}(x_j^*)\Big| \Bigg| \frac{1+\sum_{k\in\Delta_{jm}} \frac{A_{jk}A_{km}}{A_{jm}} Q_{km}}{1 - J_j H_1(x_j^*) H_2^{'}(x_j^*) \sum_{k\in L_{jm}} A_{jk}^2 Q_{kj}} - \frac{1+\sum_{k\in\Delta_{ji}} \frac{A_{jk}A_{ki}}{A_{ji}} Q_{ki}}{1 - J_j H_1(x_j^*) H_2^{'}(x_j^*) \sum_{k\in L_{ji}} A_{jk}^2 Q_{kj}} \Bigg| \\ &\leq \Big|J_j H_1(x_j^*) H_2^{'}(x_j^*)\Big| \frac{2\Delta_{max}}{\Bigg|1 - J_j H_1(x_j^*) H_2^{'}(x_j^*) \sum_{k\in L_{jm}} A_{jk}^2 Q_{kj}\Bigg|}, \end{aligned} \tag{5.16}$$

in which

$$\Delta_{max} = \max\left(\Big| \sum_{k\in\Delta_{jm}} \frac{A_{jk}A_{km}}{A_{jm}} Q_{km} \Big|, \Big| \sum_{k\in\Delta_{ji}} \frac{A_{jk}A_{ki}}{A_{ji}} Q_{ki} \Big| \right). \tag{5.17}$$

If $H_1(x_j^*)H_2^{'}(x_j^*)$ tends to zero, Q_{jm} is quite near Q_{ji}. The difference becomes smaller along with each iteration and this indicates that the iteration process is reasonable.

Next is the expression of iterative formula. The right-upper side of variable (ℓ) indicates the number of iteration. We set $Q_{im}^{(-1)} = 0$, then $\mathcal{E}_{im}^{(0)} = \mathcal{E}_{L_{im}}^{(-1)} = 0$. The rule of iteration is set as

$$\begin{cases} Q_{im}^{(\ell)} = \frac{J_i H_1(x_i^*) H_2^{'}(x_i^*)}{1-\mathcal{E}_{im}^{(\ell)}}, \\ \mathcal{E}_{im}^{(\ell)} = \frac{-1+J_i H_1(x_i^*) H_2^{'}(x_i^*) \sum_{j\in L_{im}} A_{ij}^2 Q_{jm}^{(\ell-1)}}{1+\sum_{j\in\Delta_{im}} \frac{A_{ij}A_{jm}}{A_{im}} Q_{jm}^{(\ell-1)}} + 1. \end{cases} \tag{5.18}$$

Then

$$\begin{cases} \mathcal{E}_{\Delta_{im}}^{(\ell)} = \sum_{j\in\Delta_{im}} \frac{A_{ij}A_{jm}}{A_{im}} Q_{jm}^{(\ell)}, \\ \mathcal{E}_{L_{im}}^{(\ell)} = Q_{im}^{(0)} \sum_{j\in L_{im}} A_{ij}^2 Q_{jm}^{(\ell)}. \end{cases} \tag{5.19}$$

Combining two effects into β_{im}^{ℓ} yields $\beta_{im}^{(\ell)} = J_i \left(\frac{1-\mathcal{E}_{L_i}^{(\ell-1)}}{1+\mathcal{E}_{\Delta_i}^{\ell-1}} - 1 \right)$. The iterative formula of $Q_{im}^{(\ell)}$ is

$$\begin{cases} Q_{im}^{(\ell)} = Q_{im}^{(0)} \frac{1+\mathcal{E}_{\Delta_{im}}^{\ell-1}}{1-\mathcal{E}_{L_{im}}^{(\ell-1)}}, \\ Q_{im}^{(0)} = J_i H_1(x_i^*) H_2^{'}(x_i^*) = -\frac{H_2^{'}(x_i^*)}{\left[\frac{F(x_i^*)}{H_1(x_i^*)}\right]^{'}}. \end{cases} \tag{5.20}$$

5.3 Another Way to Quantify Global Propagation Time

Here, we provide another way to quantify global propagation time based on different assumptions. After employing amended Gauss iteration method, we derive

$$T(m \to i_k) = T(m \to i_{k-1}) - \frac{\Delta x_{i_k}\big(T(m \to i_{k-1})\big) - \eta \Delta x_{i_k}(\infty)}{\Delta \dot{x}_{i_k}\big(T(m \to i_{k-1})\big) - \eta \Delta \dot{x}_{i_k}(\infty)}. \tag{5.21}$$

As $\Delta \dot{x}_{i_k}(\infty) = 0$, the equation is simplified as

$$T(m \to i_k) = T(m \to i_{k-1}) - \frac{\Delta x_{i_k}\big(T(m \to i_{k-1})\big) - \eta \Delta x_{i_k}(\infty)}{\Delta \dot{x}_{i_k}\big(T(m \to i_{k-1})\big)}. \tag{5.22}$$

After integrating the iterative formula, we obtain the response $\Delta \dot{x}_{i_k}\big(T(m \to i_{k-1})\big)$. Set $t = T(m \to i_{k-1})$ and derive

$$\begin{aligned}\Delta \dot{x}_{i_k}(T(m \to i_{k-1})) = &-\frac{1}{J_{i_k}} \Delta x_{i_k}(T(m \to i_{k-1})) + H_1(x^*_{i_k}) \sum_{\substack{j=1 \\ j \neq i_{k-1}}}^{N} A_{j i_k} H_2'(x^*_j) \Delta x_j(T(m \to i_{k-1})) \\ &+ A_{i_k i_{k-1}} H_1(x^*_{i_k}) H_2'(x^*_{i_{k-1}}) \eta \Delta x_{i_{k-1}}(\infty).\end{aligned} \tag{5.23}$$

Similarly, we set $t \to \infty$ and obtain

$$0 = -\frac{1}{J_{i_k}} \Delta x_{i_k}(\infty) + H_1(x^*_{i_k}) \sum_{\substack{j=1 \\ j \neq i_{k-1}}}^{N} A_{j i_k} H_2'(x^*_j) \Delta x_j(\infty) + A_{i_k i_{k-1}} H_1(x_{i^*_k}) H_2'(x_{i^*_{k-1}}) \Delta x_{i_{k-1}}(\infty). \tag{5.24}$$

Combining Eq. (5.23) with Eq. (5.24), we obtain that

$$\Delta \dot{x}_{i_k}(T(m \to i_{k-1})) = \frac{1}{J_{i_k}} \left(-1 + \mathcal{E}_{i_k i_{k-1}}(T(m \to i_{k-1}))\right) \left(\Delta x_{i_k}(T(m \to i_{k-1})) - \eta \Delta x_{i_k}(\infty)\right). \tag{5.25}$$

Effects towards node i_{k-1} is rewritten as

$$\mathcal{E}_{i_k i_{k-1}}(T(m \to i_{k-1})) = J_{i_k} H_1(x^*_{i_k}) \sum_{\substack{j=1 \\ j \neq i_{k-1}}}^{N} A_{i_k j} H_2'(x^*_j) \delta_{i_k j}(T(m \to i_{k-1})), \tag{5.26}$$

where

$$\delta_{ij}(T(m \to i_{k-1})) = \frac{\Delta x_j(T(m \to i_{k-1})) - \eta \Delta x_j(\infty)}{\Delta x_i(T(m \to i_{k-1})) - \eta \Delta x_i(\infty)}. \tag{5.27}$$

The quantification of propagation time $T(m \to i_k)$ becomes

$$T(m \to i_k) = T(m \to i_{k-1}) + J_{i_k} \frac{1}{1 - \mathcal{E}_{i_k i_{k-1}}(T(m \to i_{k-1}))}. \tag{5.28}$$

Similar with the local case, nodes i_{k-1}, $i_{k,1}$ and $i_{k,2}$ exhibit a triangle structure, and nodes i_{k-1}, $i_{k,1}$ and i_{k+1} form an independent edge.

Before the classification, $\delta_{ij}(T(m \to i_{k-1}))$ is simplified as

$$\begin{aligned}\delta_{ij}(T(m \to i_{k-1})) &= \frac{\frac{\Delta x_j(T(m \to i_{k-1})) - \eta \Delta x_j(\infty)}{\Delta \dot{x}_j(T(m \to i_{k-1}))}}{\frac{\Delta x_i(T(m \to i_{k-1})) - \eta \Delta x_i(\infty)}{\Delta \dot{x}_i(T(m \to i_{k-1}))}} \frac{\Delta \dot{x}_j(T(m \to i_{k-1}))}{\Delta \dot{x}_i(T(m \to i_{k-1}))}, \\ &= \frac{T(m \to j) - T(m \to i_{k-1})}{T(m \to i) - T(m \to i_{k-1})} \frac{\Delta \dot{x}_j(T(m \to i_{k-1}))}{\Delta \dot{x}_i(T(m \to i_{k-1}))}.\end{aligned} \tag{5.29}$$

We suppose that $\Delta\dot{x}_i(T(m\to i_{k-1}))$ and $\Delta\dot{x}_j(T(m\to i_{k-1}))$ keep the same linear proportions with their own variables $\Delta x_i(T(m\to i_{k-1}))$ and $\Delta x_j(T(m\to i_{k-1}))$ respectively, and $\delta_{ij}(T(m\to i_{k-1}))$ will be

$$\delta_{ij}(T(m\to i_{k-1}))=\frac{T(m\to j)-T(m\to i_{k-1})}{T(m\to i)-T(m\to i_{k-1})}\frac{\Delta x_j(T(m\to i_{k-1}))}{\Delta x_i(T(m\to i_{k-1}))}. \tag{5.30}$$

Eq. (5.30) is divided into two parts according to different topological elements exhibited by Fig. S2. Similar with the local case, structural effects are parted into sets of triangles and independent edges in global case,

$$\begin{aligned}\mathcal{E}_{i_k i_{k-1}}(T(m\to i_{k-1}))=&J_{i_k}H_1(x^*_{i_k})\sum_{j\in L_{i_k i_{k-1}}}A_{i_k j}H_2^{'}(x^*_j)\frac{T(m\to j)-T(m\to i_{k-1})}{T(m\to i_k)-T(m\to i_{k-1})}\frac{\Delta x_j(T(m\to i_{k-1}))}{\Delta x_{i_k}(T(m\to i_{k-1}))}\\&+J_{i_k}H_1(x^*_{i_k})\sum_{j\in \Delta_{i_k i_{k-1}}}A_{i_k j}H_2^{'}(x^*_j)\frac{T(m\to j)-T(m\to i_{k-1})}{T(m\to i_k)-T(m\to i_{k-1})}\frac{\Delta x_j(T(m\to i_{k-1}))}{\Delta x_{i_k}(T(m\to i_{k-1})).}\end{aligned} \tag{5.31}$$

We firstly investigate independent edges, express $\Delta x_{i_{k+1}}\big(T(m\to i_{k-1})\big)$, and obtain that

$$\begin{aligned}\Delta x_{i_{k+1}}(T(m\to i_{k-1}))T(m\to i_{k-1})^{-1}=&\frac{1}{J_{i_{k+1}}}\big(-1+\mathcal{F}_{i_{k+1}i_k}(T(m\to i_{k-1}))\big)\Delta x_{i_{k+1}}(T(m\to i_{k-1}))\\&+A_{i_{k+1}i_k}H_1\big(x^*_{i_{k+1}}\big)H_2^{'}\big(x^*_{i_k}\big)\Delta x_{i_k}(T(m\to i_{k-1})),\end{aligned} \tag{5.32}$$

in which

$$\mathcal{F}_{i_k i_{k-1}}(T(m\to i_{k-1}))=J_{i_k}H_1(x^*_{i_k})\sum_{\substack{j=1\\ j\neq i_{k-1}}}^{N}A_{i_k j}H_2^{'}(x^*_j)\frac{\Delta x_j(T(m\to i_{k-1}))}{\Delta x_{i_k}(T(m\to i_{k-1}))}. \tag{5.33}$$

Hence, $\Delta x_{i_{k+1}}\big(T(m\to i_{k-1})\big)$ is shown as

$$\frac{\Delta x_{i_{k+1}}(T(m\to i_{k-1}))}{\Delta x_{i_k}(T(m\to i_{k-1}))}=\frac{A_{i_{k+1}i_k}H_1\big(x^*_{i_{k+1}}\big)H_2^{'}\big(x^*_{i_k}\big)}{J^{-1}_{i_{k+1}}(1-\mathcal{F}_{i_{k+1}i_k}(T(m\to i_{k-1})))+T(m\to i_{k-1})^{-1}}. \tag{5.34}$$

Next, we consider nodes in the same layer. We denote $i_{k,1}$ as i_k and select another node $i_{k,2}$ in the k-th layer for distinction. This equation is influenced by so many different complicated cases, so we select the easiest case that is triangle, i.e., nodes $i_{k,2}$, $i_{k,1}$ and i_{k-1} are connected for any two.

$$\frac{\Delta x_{i_{k,2}}(T(m\to i_{k-1}))}{\Delta x_{i_{k,1}}(T(m\to i_{k-1}))}=\frac{\Delta x_{i_{k,2}}(T(m\to i_{k-1}))}{\Delta x_{i_{k-1}}(\infty)}\frac{\Delta x_{i_{k-1}}(\infty)}{\Delta x_{i_{k,1}}(T(m\to i_{k-1}))}. \tag{5.35}$$

We derive the first specific value through Eq. (5.34),

$$\begin{cases}\frac{\Delta x_{i_{k-1}}(\infty)}{\Delta x_{i_{k,1}}(T(m\to i_{k-1}))}=\frac{1}{\eta}\frac{J^{-1}_{i_{k,1}}(1-\mathcal{F}_{i_{k,1}i_{k-1}}(T(m\to i_{k-1})))+T(m\to i_{k-1})^{-1}}{A_{i_{k,1}i_{k-1}}H_1\big(x^*_{i_{k,1}}\big)H_2^{'}\big(x^*_{i_{k-1}}\big)},\\ \frac{\Delta x_{i_{k-1}}(\infty)}{\Delta x_{i_{k,2}}(T(m\to i_{k-1}))}=\frac{1}{\eta}\frac{J^{-1}_{i_{k,2}}(1-\mathcal{F}_{i_{k,2}i_{k-1}}(T(m\to i_{k-1})))+T(m\to i_{k-1})^{-1}}{A_{i_{k,2}i_{k-1}}H_1\big(x^*_{i_{k,2}}\big)H_2^{'}\big(x^*_{i_{k-1}}\big)}.\end{cases} \tag{5.36}$$

The specific value is

$$\frac{\Delta x_{i_{k,2}}(T(m\to i_{k-1}))}{\Delta x_{i_{k,1}}(T(m\to i_{k-1}))}=\frac{A_{i_{k,2}i_{k-1}}}{A_{i_{k,1}i_{k-1}}}\frac{H_1\big(x^*_{i_{k,2}}\big)}{H_1\big(x^*_{i_{k,1}}\big)}\frac{J^{-1}_{i_{k,1}}(1-\mathcal{F}_{i_{k,1}i_{k-1}}(T(m\to i_{k-1})))+T(m\to i_{k-1})^{-1}}{J^{-1}_{i_{k,2}}(1-\mathcal{F}_{i_{k,2}i_{k-1}}(T(m\to i_{k-1})))+T(m\to i_{k-1})^{-1}}. \tag{5.37}$$

As the node $i_{k,1}$ is mainly focused in the following part, we denote $i_{k,1}$ as i_k for convenience. Combining Eqs. (5.34) and (5.37), we derive

$$\mathcal{E}_{i_k i_{k-1}}(T(m \to i_{k-1})) = Q_{i_k} \sum_{j \in L_{i_k i_{k-1}}} A_{j i_k}^2 R_{j i_k}(T(m \to i_{k-1})) \frac{T(m \to j) - T(m \to i_{k-1})}{T(m \to i_k) - T(m \to i_{k-1})} + \frac{Q_{i_k}}{R_{i_k i_{k-1}}(T(m \to i_{k-1}))} \sum_{j \in \Delta_{i_k i_{k-1}}} \frac{A_{i_k j} A_{j i_{k-1}}}{A_{i_k i_{k-1}}} R_{j i_{k-1}}(T(m \to i_{k-1})) \frac{T(m \to j) - T(m \to i_{k-1})}{T(m \to i_k) - T(m \to i_{k-1})}, \tag{5.38}$$

in which

$$R_{j i_k}(T(m \to i_{k-1})) = \frac{Q_j}{1 - \mathcal{F}_{j i_k}(T(m \to i_{k-1})) + J_j T(m \to i_{k-1})^{-1}}. \tag{5.39}$$

After substituting the expression of $\mathcal{E}_{i_k i_{k-1}}(T(m \to i_{k-1}))$ into Eq. (5.28), the propagation time becomes

$$T(m \to i_k) = T(m \to i_{k-1}) + J_{i_k} + Q_{i_k} \sum_{j \in L_{i_k i_{k-1}}} A_{i_k j}^2 R_{j i_k}(T(m \to i_{k-1})) \left(T(m \to j) - T(m \to i_{k-1})\right) + \frac{Q_{i_k}}{R_{i_k i_{k-1}}(T(m \to i_{k-1}))} \sum_{j \in \Delta_{i_k i_{k-1}}} \frac{A_{i_k j} A_{j i_{k-1}}}{A_{i_k i_{k-1}}} R_{j i_{k-1}}(T(m \to i_{k-1})) \left(T(m \to j) - T(m \to i_{k-1})\right). \tag{5.40}$$

Further simplifying the expression, we obtain

$$T(m \to i_k) = T(m \to i_{k-1}) + J_{i_k} + \mathcal{E}_{L_{i_k i_{k-1}}}(T(m \to i_{k-1})) + \mathcal{E}_{\Delta_{i_k i_{k-1}}}(T(m \to i_{k-1})), \tag{5.41}$$

in which

$$\begin{cases} \mathcal{E}_{L_{i_k i_{k-1}}}(T(m \to i_{k-1})) = Q_{i_k} \sum_{j \in L_{i_k i_{k-1}}} A_{i_k j}^2 \left(T(m \to j) - T(m \to i_{k-1})\right) R_{j i_k}(T(m \to i_{k-1})), \\ \mathcal{E}_{\Delta_{i_k i_{k-1}}}(T(m \to i_{k-1})) = \frac{Q_{i_k}}{R_{i_k i_{k-1} T(m \to i_{k-1})}} \sum_{j \in \Delta_{i_k i_{k-1}}} \frac{A_{i_k j} A_{j i_{k-1}}}{A_{i_k i_{k-1}}} \left(T(m \to j) - T(m \to i_{k-1})\right) R_{j i_{k-1}}(T(m \to i_{k-1})). \end{cases} \tag{5.42}$$

Main terms generating the expression of propagation time are

$$\begin{cases} J_j = 1/\left(-H_1(x_j^*)\left[\frac{F(x_j^*)}{H_1(x_j^*)}\right]'\right), \\ Q_j = J_j H_1(x_j^*) H_2'(x_j^*), \\ R_{j i_k}(T(m \to i_{k-1})) = \frac{Q_j}{1 - \mathcal{F}_{j i_k}(T(m \to i_{k-1})) + J_j T(m \to i_{k-1})^{-1}}. \end{cases} \tag{5.43}$$

From a global perspective, we find that propagation time is influenced by four factors: intrinsic dynamic J_i, effect of independent edges $\mathcal{E}_{L_{i_k i_{k-1}}}$, effect of triangles $\mathcal{E}_{\Delta_{i_k i_{k-1}}}$ and length of chain k. We will discuss about how these factors in detail. For the effect of triangles, we have

$$\mathcal{E}_{\Delta_{i_k i_{k-1}}}(T(m \to i_{k-1})) = \frac{Q_{i_k}}{R_{i_k i_{k-1}}} \sum_{j \in \Delta_{i_k m}} \frac{A_{i_k j} A_{j i_{k-1}}}{A_{i_k i_{k-1}}} \left(T(m \to j) - T(m \to i_{k-1})\right) R_{j i_{k-1}}(T(m \to i_{k-1})). \tag{5.44}$$

Similar with the local case, we transform the coupling terms into the mean forms through mean−field theory,

$$\mathcal{R}_\Delta = \frac{1}{d_{i_{k-1}}} \sum_{i_k \in K_{i_{k-1}}} \frac{1}{t_{i_k i_{k-1}}} \sum_{j \in \Delta_{i_k i_{k-1}}} \frac{A_{i_k j} A_{j i_{k-1}}}{A_{i_k i_{k-1}}} \left(T(m \to j) - T(m \to i_{k-1})\right) R_{j i_{k-1}}(T(m \to i_{k-1})), \tag{5.45}$$

and Eq. (5.44) becomes

$$\mathcal{E}_{\Delta_{i_k i_{k-1}}}(T(m \to i_{k-1})) = \frac{Q_{i_k}}{R_{i_k i_{k-1}}} t_{i_k i_{k-1}} \mathcal{R}_{\Delta}. \tag{5.46}$$

Through Eq. (5.46), trends of $\mathcal{E}_{\Delta_{i_k i_{k-1}}}(T(m \to i_{k-1}))$ and $t_{i_k i_{k-1}}$ are the same. We expand the result to the whole network

$$\Gamma_{im} = \min_{\Pi(m \to i)} \left\{ \sum_{\substack{i_q \in \Pi(m \to i) \\ i_q \neq m}} \left(J_{i_q} + \mathcal{E}_{\Delta_{i_q i_{q-1}}} \right) \right\}, \tag{5.47}$$

in which $\Pi(m \to i)$ is the set of the shortest paths between nodes m and i. Hence, it holds $\Gamma_{im} \propto T(m \to i)$. Only concerning about effect of independent edges for a global perspective, we consider a network comprising a single chain. The iterative formula under this specific network is

$$T(m \to i_k) = T(m \to i_{k-1}) + J_{i_k} + \mathcal{E}_{L_{i_k i_{k-1}}}(T(m \to i_{k-1})). \tag{5.48}$$

Iterative equations are

$$\begin{cases} J_i = -H_1(x_i^*) \left[\frac{F(x_i^*)}{H_1(x_i^*)} \right]', \\ \mathcal{E}_{L_{i_k i_{k-1}}}(T(m \to i_{k-1})) = \mathcal{F}_{L_{i_k i_{k-1}}}(T(m \to i_{k-1})) - Q_{i_k} T(m \to i_{k-1}) s_{i_k i_{k-1}} \mathcal{R}_L, \end{cases} \tag{5.49}$$

in which

$$\begin{cases} \mathcal{F}_{L_{i_k i_{k-1}}}(T(m \to i_{k-1})) = Q_{i_k} \sum_{j \in L_{i_k i_{k-1}}} A_{i_k j}^2 T(m \to j) R_{j i_k}(T(m \to i_{k-1})), \\ \mathcal{R}_L = \frac{1}{d_{i_{k-1}}} \sum_{i_k \in K_{i_{k-1}}} \frac{1}{s_{i_k i_{k-1}}} \sum_{j \in L_{i_k i_{k-1}}} A_{i_k j}^2 R_{j i_k}(T(m \to i_{k-1})). \end{cases} \tag{5.50}$$

$s_{i_k i_{k-1}}$ here denotes the number of independent edges. The difference between time reaching node i_k and i_{k-1} is expressed as

$$T(m \to i_k) = (1 - Q_{i_k} s_{i_k i_{k-1}} \mathcal{R}_L) T(m \to i_{k-1}) + J_{i_k} + \mathcal{F}_{L_{i_k i_{k-1}}}(T(m \to i_{k-1})). \tag{5.51}$$

Supplementary References

[1] Chittaranjan Hens, Uzi Harush, Simi Haber, Reuven Cohen, and Baruch Barzel. Spatiotemporal signal propagation in complex networks. *Nature Physics*, 15(4):403–412, 2019.

[2] Leopold Schmetterer and Karl Sigmund. *Hans Hahn Gesammelte Abhandlungen Band 1/Hans Hahn Collected Works Volume 1: Mit einem Geleitwort von Karl Popper/With a Foreword by Karl Popper*. Springer, 1995.

[3] Chittaranjan Hens, Uzi Harush, Simcha Haber, Reuven Cohen, and Baruch Barzel. Reply to: Asymptotic scaling describing signal propagation in complex networks. *Nature Physics*, 16(11):1084–1085, November 2020.

[4] Chittaranjan Hens, Uzi Harush, Simcha Haber, Reuven Cohen, and Baruch Barzel. Response times of nodes in a complex network environment–two potential derivation tracks. *arXiv preprint arXiv:2006.04738*, 2020.

[5] Peng Ji, Wei Lin, and Jürgen Kurths. Asymptotic scaling describing signal propagation in complex networks. *Nature Physics*, pages 1–3, November 2020.

[6] Marc Timme and Malte Schröder. Disentangling scaling arguments to empower complex systems analysis. *Nature Physics*, 16(11):1086–1088, November 2020.

[7] Stuart Kauffman. The ensemble approach to understand genetic regulatory networks. *Physica A: Statistical Mechanics and its Applications*, 340(4):733–740, 2004.

[8] Guy Karlebach and Ron Shamir. Modelling and analysis of gene regulatory networks. *Nature reviews Molecular cell biology*, 9(10):770–780, 2008.

[9] Hoi-To Wai, Anna Scaglione, Uzi Harush, Baruch Barzel, and Amir Leshem. Rids: Robust identification of sparse gene regulatory networks from perturbation experiments. *arXiv preprint arXiv:1612.06565*, 2016.

[10] Uri Alon. *An introduction to systems biology: design principles of biological circuits*. CRC press, 2019.

[11] Santhanam Balaji, M Madan Babu, Lakshminarayan M Iyer, Nicholas M Luscombe, and Lakshminarayan Aravind. Comprehensive analysis of combinatorial regulation using the transcriptional regulatory network of yeast. *Journal of molecular biology*, 360(1):213–227, 2006.

[12] Jean-François Rual, Kavitha Venkatesan, Tong Hao, Tomoko Hirozane-Kishikawa, Amélie Dricot, Ning Li, Gabriel F Berriz, Francis D Gibbons, Matija Dreze, Nono Ayivi-Guedehoussou, et al. Towards a proteome-scale map of the human protein–protein interaction network. *Nature*, 437(7062):1173–1178, 2005.

[13] Claudio Castellano, Santo Fortunato, and Vittorio Loreto. Statistical physics of social dynamics. *Reviews of modern physics*, 81(2):591, 2009.

[14] Peter Sheridan Dodds and Duncan J Watts. A generalized model of social and biological contagion. *Journal of theoretical biology*, 232(4):587–604, 2005.

[15] Robert M May. Simple mathematical models with very complicated dynamics. *The Theory of Chaotic Attractors*, pages 85–93, 2004.

[16] Eberhard O Voit. *Computational analysis of biochemical systems: a practical guide for biochemists and molecular biologists*. Cambridge University Press, 2000.

[17] Crispin Gardiner, Peter Zoller, and Peter Zoller. *Quantum noise: a handbook of Markovian and non-Markovian quantum stochastic methods with applications to quantum optics*. Springer Science & Business Media, 2004.

[18] Uzi Harush and Baruch Barzel. Dynamic patterns of information flow in complex networks. *Nature communications*, 8(1):1–11, 2017.

[19] Chandrakala Meena, Chittaranjan Hens, Suman Acharyya, Simcha Haber, Stefano Boccaletti, and Baruch Barzel. Emergent stability in complex network dynamics. *arXiv preprint arXiv:2007.04890*, 2020.
